# Synthesis and α-Glucosidase Inhibition Activity of 2-[3-(Benzoyl/4-bromobenzoyl)-4-hydroxy-1,1-dioxido-2*H*-benzo[*e*][1,2]thiazin-2-yl]-*N*-arylacetamides: An In Silico and Biochemical Approach

**DOI:** 10.3390/molecules26103043

**Published:** 2021-05-20

**Authors:** Furqan Ahmad Saddique, Sana Aslam, Matloob Ahmad, Usman Ali Ashfaq, Muhammad Muddassar, Sadia Sultan, Saman Taj, Muzammil Hussain, Dae Sung Lee, Magdi E. A. Zaki

**Affiliations:** 1 Department of Chemistry, Government College University, Faisalabad 38000, Pakistan; furqanas123@gmail.com; 2 Department of Chemistry, Government College Women University, Faisalabad 38000, Pakistan; Dr.Sana@gcwuf.edu.pk; 3 Department of Bioinformatics and Biotechnology, Government College University, Faisalabad 38000, Pakistan; saman20220@yahoo.com; 4 Department of Biosciences, COMSATS University Islamabad, Park Road, Islamabad 45500, Pakistan; mmuddassar@comsats.edu.pk; 5 Faculty of Pharmacy, Universiti Teknologi MARA, Puncak Alam Campus, Bandar Puncak Alam 42300, Selangor Darul Ehsan, Malaysia; drsadia@uitm.edu.my; 6 Atta-ur-Rahman Institute for Natural Products Discovery (AuRIns), Universiti Teknologi MARA, Puncak Alam Campus, Bandar Puncak Alam 42300, Selangor Darul Ehsan, Malaysia; 7 Department of Environmental Engineering, Kyungpook National University, Daegu 41566, Korea; muzammil@knu.ac.kr (M.H.); daesung@knu.ac.kr (D.S.L.); 8 Department of Chemistry, Faculty of Science, Imam Mohammad Ibn Saud Islamic University (IMSIU), Riyadh 11623, Saudi Arabia

**Keywords:** 1,2-Benzothiazines, synthesis, molecular docking, α-glucosidase inhibition, anti-diabetic

## Abstract

Diabetes mellitus (DM) is a chronic disorder and has affected a large number of people worldwide. Insufficient insulin production causes an increase in blood glucose level that results in DM. To lower the blood glucose level, various drugs are employed that block the activity of the α-glucosidase enzyme, which is considered responsible for the breakdown of polysaccharides into monosaccharides leading to an increase in the intestinal blood glucose level. We have synthesized novel 2-(3-(benzoyl/4-bromobenzoyl)-4-hydroxy-1,1-dioxido-2*H*-benzo[*e*][1,2]thiazin-2-yl)-*N*-arylacetamides and have screened them for their in silico and in vitro α-glucosidase inhibition activity. The derivatives **11c**, **12a**, **12d**, **12e**, and **12g** emerged as potent inhibitors of the α-glucosidase enzyme. These compounds exhibited good docking scores and excellent binding interactions with the selected residues (Asp203, Asp542, Asp327, His600, Arg526) during in silico screening. Similarly, these compounds also showed good in vitro α-glucosidase inhibitions with IC_50_ values of 30.65, 18.25, 20.76, 35.14, and 24.24 μM, respectively, which were better than the standard drug, acarbose (IC_50_ = 58.8 μM). Furthermore, a good agreement was observed between in silico and in vitro modes of study.

## 1. Introduction

Diabetes mellitus (DM) is an endocrine system illness and causes metabolic ailment that leads to hyperglycemia. DM is characterized by increased blood sugar levels either due to the body’s reduced production of insulin or ineffective utilization of available insulin by the body. Chronic hyperglycemia results in neuron oxidative damage while prolonged hyperglycemia causes the production of lower caloric content of sugar in different organs [1,2]. Although genetic predisposition can be a factor in the development of DM, other major causing factors may include sedentariness, smoking, and intake of high-saturated fatty meals [3,4]. Change in lifestyle can decrease the risk of type 2 diabetes by 58%, for example, overweight people can avoid it by losing weight, a fact explained experimentally by the Finnish prevention trial [5] and the US diabetes prevention program [6]. DM also enhances the chances of cardiovascular diseases [7]. People suffering with hypertension are already leading a risky life due to high blood pressure [8] but they often develop DM and this combination of risks increases cardiovascular transience. The rate of DM is growing rapidly worldwide especially in developing countries [9]. The International Diabetes Federation (IDF) has observed that 371 million humans are infected with T2DM (type 2 diabetes mellitus) while an increase rate of 2% annually has been estimated. α-Amylase and α-glucosidase are the two enzymes that have a key role in the breakdown of polysaccharides to monosaccharides [10]. α-Glucosidase enzyme, released from mucosal cells, usually converts polysaccharides into monosaccharides causing a gradual increase in the absorption of glucose and hence increases intestinal blood glucose level [11]. These enzymes have role in breakdown and absorption of polysaccharides, so by inhibiting the activity of these enzymes, glucose levels in diabetic patients can be controlled even after consumption of carbohydrate-rich diets [12].

Different α-glucosidase and α-amylase inhibitors are being used for delaying glucose absorption in the blood, such as acarbose, voglibose, miglitol, etc., but this medication has many side effects [13,14,15,16,17], such as diarrhea, gastrointestinal distress (flatulence, pain), and liver disorders (hepatic injury, hepatitis, and renal tumors) [18,19,20,21,22]. Hence, there is a need in the present era to develop more potent antidiabetic agents with minimum side effects.

**Figure 1:**
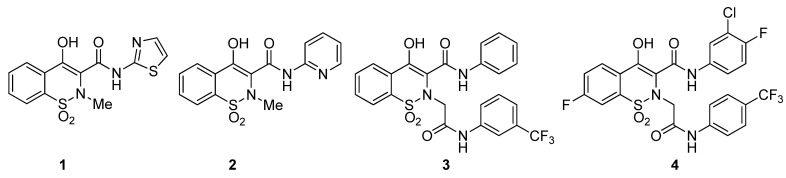
Structures of biologically important 1,2-benzothiazine 1,1-dioxides.

Benzothiazines are well-known heterocycles due to their wide spectrum of pharmacological activities. Among the various types of benzothiazines, 1,2-benzothiazine 1,1-dioxides and their derivatives have broadly been studied. Prominently, 1,2-benzothiazine 1,1-dioxide carboxamides have a major footprint in clinical therapy due to their anti-inflammatory activity. As revealed by the literature, sudoxicam **1** (Figure 1) is an important oxicam having an extended plasma half-life [23], piroxicam **2** (Figure 1), another potent oxicam, is used to cure rheumatoid arthritis, osteoarthritis, and other inflammations [24]. Similarly, various 1,2-benzothiazine 1,1-dioxides have been reported as antileukemic [25,26,27], 11*β*-HSD1 inhibitors [28], endotheline receptor antagonists [29], antimalarial agents [30], MAO inhibitors [31], antiallergic agents [32], antithrombotics [33], antiparasitic [34], antimicrobials [35,36], antioxidants [37], inhibitors of calpain I [38], and aldose reductase enzymes [39,40].

Our research group is involved in the search for novel and more effective antidiabetic agents [41,42,43,44,45], and in this context we have synthesized novel 1,2-benzothiazine-*N*-arylacetamides and screened them for their in silico and in vitro α-glucosidase inhibitory potential.

## 2. Results and Discussion

### 2.1. Synthetic Chemistry

**Scheme 1:**
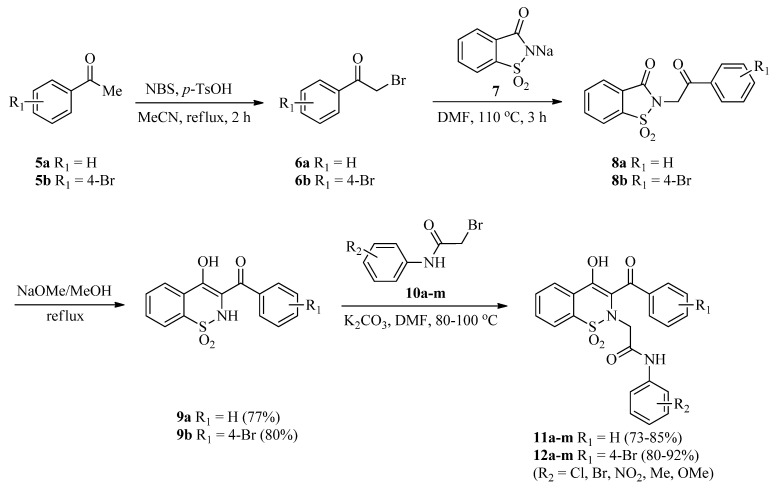
Synthesis of 1,2-benzothiazine-*N*-arylacetamides **11a**–**m** and **12a**–**m**.

The precursors, 3-(benzoyl/4-bromobenzoyl)-1,2-benzothazines **9a**,**b**, were synthesized via reported methods involving Gabriel–Colman rearrangement [46,47]. Later on, 3-(benzoyl/4-bromobenzoyl)-1,2-benzothazines **9a**,**b** were treated with a library of 2-bromo-*N*-arylacetamides **10a**–**m** (synthesized as described previously [48]) in the presence of a potassium carbonate base in a DMF solvent. Reactions were completed in 2–2.5 h (as indicated by TLC) and then dilution with distilled water followed by acidification with 20% HCl afforded the targeted 1,2-benzothiazine-*N*-arylacetamides **11a**–**m** and **12a**–**m** good to excellent yields (73–92%) (Scheme 1). Increase in reaction time was not helpful regarding the yields of products. Similarly, use of low boiling solvents like acetonitrile also resulted in good yields but in a longer reaction time (4 h).

### 2.2. Spectroscopic Characterization

All the synthesized derivatives were well characterized with ^1^H-NMR, ^13^C-NMR, and MS spectroscopic techniques. In the ^1^H-NMR spectra, the methylene (CH_2_) protons appeared as a singlet at 3.95–4.20 ppm and the observation was consistent with literature values of similar compounds [46,49]. The methyl protons (CH_3_) and methoxy protons appeared as singlets at 1.96–2.17 and 3.64–3.86 ppm, respectively. The NH and enolic–OH protons were observed as singlets at 9.16–10.54 and 14.92–15.18 ppm, respectively [37,49,50]. Peaks for enolic–OH protons were not observed in some spectra due to the exchangeable nature of OH protons [49]. All the peak values from 6.50–8.50 ppm were assigned to aromatic protons. In MS spectra, the observed molecular ion peaks were found in accordance with the calculated values.

### 2.3. Biological Activity

Diabetes mellitus is a severe chronic metabolic syndrome that is illustrated by hyperglycemia and escorted by different severe vascular complications. The main therapy of diabetes is to control the postprandial blood-glucose level, however, limited uptake of carbohydrates after meals is also considered as a therapy. *α*-Amylase and *α*-glucosidase enzymes are responsible for the breakdown of polysaccharides to monosaccharides. Consequently, inhibition of these enzymes leads to treatment postprandial hyperglycemia by interrupting glucose absorption levels. As described above, the 1,2-benzothiazine 1,1-dioxides are valuable heterocycles and possess various biological activities. In particular, 1,2-benzothiazine 1,1-dioxide based carboxamides are the most biologically active templates. In 2011, Kim and colleagues synthesized different 1,2-benzothiazine-*N*-arylacetamides and screened them for their in vitro inhibition toward human 11*β*-HSD1. The derivative **3** (Figure 1) showed the most promising activity with an IC_50_ value of 0.041 μM [49]. Recently, Shin and coworkers reported novel 1,2-benzothiazine-*N*-arylacetamides as SARS-CoV-2 inhibitors and among them compound **4** (Figure 1) showed robust activity with an IC_50_ value of 0.88 μM [51]. Furthermore, 2-(2-(4-bromo-2-fluorobenzyl)-1,1-dioxido-2*H*-benzo[*e*][1,2]thiazin-4(3*H*)-ylidene)acetic acid was proved to be a good aldose-reductase inhibitor with an IC_50_ value of 0.11 μM [39]. Similarly, 4-hydroxy-2-methyl-*N’*-(1-phenylethylidene)-2*H*-benzo[*e*][1,2]thiazine-3-carbohydrazide 1,1-dioxide and 4-hydroxy-*N’*-(1-phenylethylidene)-2*H*-benzo[*e*][1,2]thiazine-3-carbohydrazide 1,1-dioxide showed excellent inhibitions of α-glucosidase enzymes with IC_50_ values of 4.2 and 3.9, respectively [41,45]. Encouraged by these results and to explore further biological potentials, the present study highlighted the antidiabetic activity of novel 1,2-benzothiazine-*N*-arylacetamides **11a**–**m** and **12a**–**m**.

#### 2.3.1. Molecular Docking (In Silico Analysis)

In silico screening is an excellent approach for the screening of libraries of compounds in a short time and thus it minimizes the laborious synthetic work [52,53,54,55]. Molecular docking is a widely used method to predict binding interactions between the 3D conformations of various ligands and receptor proteins that helps in optimization and leads toward drug development [56,57,58].

Regarding the effectiveness of this tool, we executed molecular docking studies of all the synthesized 1,2-benzothiazine derivatives. Molecular docking was performed via MOE software to screen out the compounds having the best residue binding interactions with the receptor protein (α-glucosidase). These compounds were ranked on the basis of root-mean-square deviation (rmsd) values, bindings with the selected residues (Asp203, Asp542, Asp327, His600, Arg526) (Figure S1), and docking scores [59].

**Figure 2:**
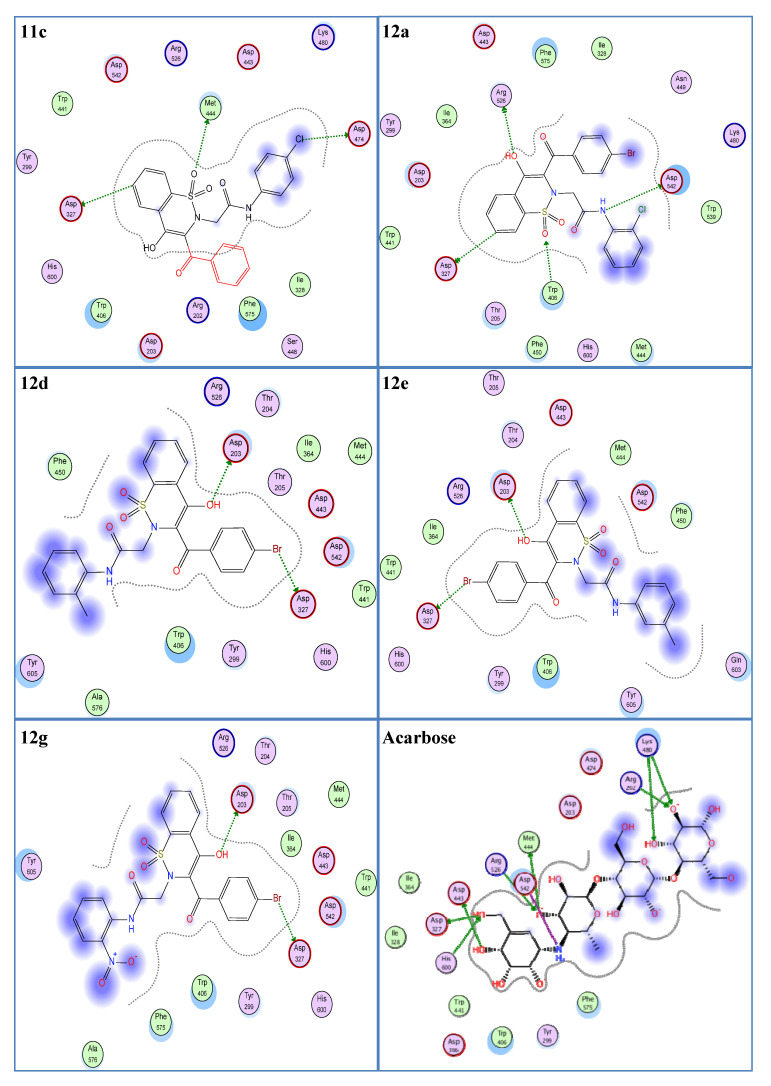
Two-dimensional interaction modes of the most active compounds (**11c**, **12a**, **12d**, **12e**, **12g**) and the standard drug, acarbose. Blue color indicates ligand exposure and a green color dotted line indicates the interactions of receptor enzymes with ligands.

**Figure 3:**
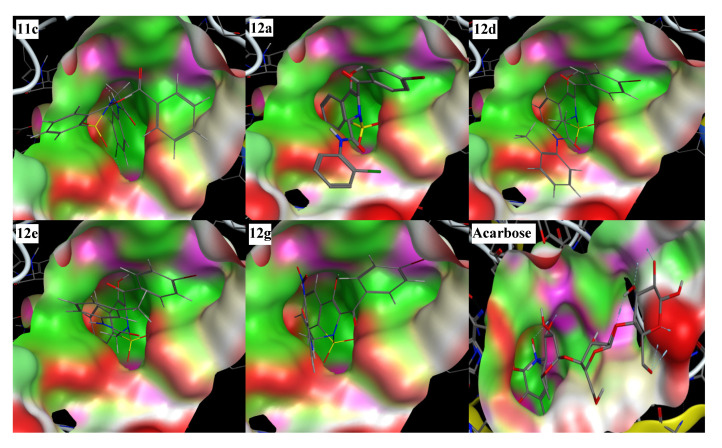
Three-dimensional interaction modes of the most active compounds (**11c**, **12a**, **12d**, **12e**, **12g**) and the standard drug, acarbose. Color portions show pocket selected and red portions indicate the ligand exposure points.

In the molecular docking experiment, most of the compounds showed good binding scores and binding interactions with the selected residues (Asp203, Asp542, Asp327, His600, Arg526) (Table 1, Figure 2). In silico studies of salacinol having a cyclic thiosugar sulfonium and a sulfate group in its structure and its derivatives found it blocked the α-glucosidase activity via bindings with the Asp203, Asp327, and Asp542 residues [60]. Recently, in silico analysis of anthocyanidins and anthocyanins was carried out that showed these scaffolds to be a good inhibitor of α-glucosidase enzymes. These compounds also showed their inhibitory action by interacting with the selected residues, Asp203, Asp327, and Asp542 [61]. Similarly, aglycone of curculigoside A and its derivatives also exhibited good in silico inhibition of α-glucosidase enzymes via interactions with Asp203, Asp327, and Asp542 residues [62]. The ligands **12a**, **12d**, **12e**, and **12g** having docking scores from −13.47 to −14.23 Kcal/mol were ranked the most effective antidiabetic scaffolds. Moreover, compounds **11c**, **11h**, **11i**, and **12m** were also considered important due to good binding energy values in the range of −12.13 to −12.80 Kcal/mol. These above-described ligands showed binding energy values close to the standard drug, acarbose (−16.18 Kcal/mol) and were tightly packed in the selected pocket of the receptor enzyme as shown in 3D maps (Figure 3). The 2D interaction modes revealed that most of the ligands bind with Asp203, Asp327, and Asp542 among the selected residues and were supposed to be responsible for the α-glucosidase inhibitory activity (Figure 2, Table S1). Acarbose (control) also showed good interactions with the selected residues, His600, Asp542, Arg526, and Asp327, in the re-docking experiment. The docking score and rmsd value for acarbose were found as −16.18 Kcal/mol and 2.00 Å, respectively.

As a whole, 1,2-Benzothiazine-*N*-arylacetamides **11a**–**m** were observed to be less effective compared to derivatives **12a**–**m** regarding their in silico α-glucosidase inhibitory potential (Table 1). However, compounds **11c**, **11h**, and **11i** showed comparatively better in silico enzyme inhibitions as revealed from their low binding energies and rmsd values (Table 1). The compound **11c** was observed as the most effective derivative in terms of binding energy and rmsd value, which were found to be −12.80 Kcal/mol and 1.08 Å, respectively. This ligand showed interactions with Asp327, Asp474, and Met444 residues. Asp327 interacted with the benzene ring of the benzothiazine nucleus. Asp474 formed a hydrogen bond with the chloro and Met444 with the oxygen atom of the SO_2_ group. The ligand **11h** exhibited a good docking score (−12.13 Kcal/mol) and low rmsd value (1.21 Å). A hydrogen bonding was observed between the NH group and the Asp542 residue while Thr205 interacted with the oxygen of the CO group. Similarly, a low docking score (−12.25 Kcal/mol) and rmsd value (1.11 Å) were found for the compound **11i**. This is explained by the strong hydrogen bonding of this compound with the selected residue, Asp542, via NH functionality.

1,2-Benzothiazine-*N*-arylacetamides **12a**–**m** preferentially exhibited good binding energies and better interactions with the selected residues compared to 1,2-benzothiazine-*N*-arylacetamides **11a**–**m** (Table 1). Compound **12a** was observed as the most potent compound as it showed a low docking score (−14.23 Kcal/mol), small rmsd value (1.21 Å), and good interactions with selected residues (Arg526, Asp542, Asp327). This ligand exhibited a hydrogen bond with Arg526 via the oxygen atom of the OH group. A second hydrogen interaction was found between the nitrogen atom of the NH group and the Asp542 residue. Similarly, Asp327 interacted with the benzene ring of the benzothiazine nucleus of ligand **12a**. In addition, the present ligand also formed an interaction with Trp406 using the oxygen atom of the SO_2_ group. These interactions and smaller rmsd value are responsible for the low binding energy value of ligand **12a**. The compounds **12d**, **12e**, and **12g** also showed good docking scores (−13.91, −13.47, and −13.52 Kcal/mol, respectively) due to dipole–dipole interactions with Asp203 and Asp327 via their OH and Br functionalities, respectively (Figure 2). These ligands (**12d**, **12e**, and **12g**) also exhibited low rmsd values of 1.13, 1.27, and 1.01 Å, respectively. In the ligand **12m**, the bromo group showed an interaction with the selected residue, Asp327. Due to this interaction and a low rmsd value of 1.19 Å, ligand **12m** showed a good binding energy value of −12.32 Kcal/mol.

The compound **12k** was found somewhat less effective as an inhibitor as revealed from its docking score and rmsd value (Table 1). The compound **12k** showed interactions with Asp327 and Tyr605 via both the bromo groups individually. The other members of this series showed high rmsd values and no interactions with the selected residues. In addition, the 1,2-benzothiazines **9a**,**b** were also docked into the receptor pocket but these proved to be noninhibitors of the α-glucosidase enzyme (Table 1 and Table S1).

It is concluded from the above discussion that not only the benzothiazine ring system but also the presence of the acetamide group was found indispensable for the in silico inhibition of α-glucosidase enzymes. Furthermore, it was noticed that the presence of polar as well as nonpolar groups in the structures of scaffolds played a crucial role toward the inhibition of enzymes by interacting with the selected residue. It is clear from the 2D diagrams of molecules that the interactions with the selected residues groups, such as CO, CONH, NH, SO_2_, Cl, Br, OH, phenyl, and aryl, all participated equally. Compounds with low rmsd values having good binding energies and interactions with the selected residues were of our interest.

#### 2.3.2. α-Glucosidase Inhibition (In Vitro Analysis)

In order to assess the in silico results, all the compounds were also subjected to the in vitro inhibition of α-glucosidase enzymes, which was investigated using the *p*-nitrophenyl-α-d-glucopyranoside (PNPG) substrate. Acarbose was employed as the reference drug. In vitro analysis of all the synthesized compounds was performed in order to justify the validity of in silico outcomes. Results were found in good agreement with the in silico study. Initially, percentage inhibition values were determined and then the compounds having 50% inhibition values or above were subjected to the micro dilution experiment (Table 1). As previously stated, 1,2-benzothiazine-*N*-arylacetamides **12a**–**m** were witnessed as the most effective antidiabetic agents during the in silico analysis. Herein, during in vitro screening these derivatives were also proved effective α-glucosidase inhibitors compared to 1,2-benzothiazine-*N*-arylacetamides **11a**–**m**. Among all the compounds, **11c**, **12a**, **12d**, **12e**, and **12g** showed the most promising α-glucosidase inhibition with IC_50_ values of 30.65, 18.25, 20.76, 35.14, and 24.24 μM, respectively (Table 1). These inhibitors have IC_50_ values even less than the standard drug, acarbose (58.8 μM [42,43]). These compounds showed more potent α-glucosidase inhibitions compared to the previously reported sulfonamides [63], dihydroxy pyrrolidines [64], and thiobarbituric acid enamine derivatives [65]. The compounds **11h**, **11i**, and **12m** having IC_50_ values 47.13, 40.41, and 58.45 μM, respectively, were witnessed as the moderate antidiabetic agents while other derivatives failed to show any promising inhibitory effects against α-glucosidase enzymes and were categorized as weak inhibitors or in-active compounds.

Derivatives **11a**–**m** with 3-benzoyl functionality exhibited low antidiabetic activity compared to derivatives **12a**–**m**, which was also observed during in silico studies. In this series, the presence of deactivating groups (like Cl and NO_2_) in the structure resulted in better enzyme inhibitions. However, herein, the presence of EWGs at the *para*-position of the *N*-phenyl ring was proved more effective compared to other sites as indicated by the IC_50_ values of compounds **11c** (30.65 μM), **11h** (47.13 μM), and **11i** (40.41 μM). Similarly, for the derivatives **12a**–**m**, the presence of electron withdrawing groups (weak or strong) and weak electron donating groups (like CH_3_) at the *ortho*-position of the *N*-phenyl group resulted in better antidiabetic activity as indicated by low IC_50_ values of derivatives **12a** (18.25 μM), **12d** (20.76 μM), and **12g** (24.24 μM). The derivatives **12a** and **12d** were found to be roughly three-fold more effective as inhibitors of α-glucosidase enzymes compared to acarbose. The increase in bioactivity with the introduction of EWGs in the benzoyl and *N*-phenyl moieties was also witnessed during the evaluation of previously reported 1,2-benzothiazine-*N*-arylacetamides as SARS-CoV-2 inhibitors [51]. In the series **12a**–**m**, the variation in the position of nitro, chloro, and bromo substituents failed to enhance the enzymatic inhibition potential of the compounds.

## 3. Materials and Methods

### 3.1. General

Different instruments were used for the identification of structural features of synthesized compounds. For the determination of melting points, Stuart apparatus (Open capillary *SMP3*) was employed. ^1^H and ^13^C-NMR spectra were recorded with the help of an NMR-spectrophotometer (Bruker/Hazimin) at 600 MHz using a deuterated DMSO solvent. Chemical shift values (δ) were given in ppm (parts per million) relative to tetramethylsilane (TMS) as an internal standard. Coupling constants (*J*) were given in Hertz. For the progress of chemical reactions, silica-coated TLC-plates (Merk, 0.2 mm 60 HF254) were used. Spots were visualized under a UV lamp (254 nm).

### 3.2. General Procedure for the Synthesis of 3-(Benzoyl/4-bromobenzoyl)-1,2-benzothazines (**9a**,**b**)

Acetophenones (**5a**,**b**) (1.0 mmol) and *p*-toluenesulfonic acid monohydrate (285 mg, 1.5 mmols) were dissolved in acetonitrile (10 mL). Subsequently, *N-*bromosuccinamide (196 mg, 1.10 mmols) was added in portions and the mixture was refluxed for 2 h. Reaction mixture was cooled to room temperature and solvent was evaporated under vacuum. The residue obtained was dissolved in dichloromethane (DCM) and washed with distilled water followed by the saturated solution of sodium bicarbonate. After separating the DCM layer from aqueous layer using a separating funnel, the DCM was evaporated with the help of a rotary evaporator to get α-bromoacetophenones (**6a**,**b**). α-Bromoacetophenones (**6a**,**b**) (1.0 mmol) were added to a sodium saccharin **7** (226 mg, 1.10 mmol) suspension made in DMF (5 mL) and the mixture was heated at 110 °C for 3 h to get the *N-*alkylated saccharin derivatives (**8a**,**b**). The dried *N-*alkylated saccharin derivatives (**8a**,**b**) (1.0 mmol) were refluxed with sodium methoxide (270 mg, 5.0 mmols) in dry methanol for 30 min. The mixture was cooled and treated with 15% cold HCl to get the 3-(benzoyl/4-bromobenzoyl)-1,2-benzothiazines (**9a**,**b**) [46,47] in good isolated yields (given below). The products were also re-crystallized with methanol.

*(4-Hydroxy-1,1-dioxido-2H-benzo[e][1,2]thiazin-3-yl)(phenyl)methanone* (**9a**): Yellow solid; yield: (232 mg, 77%); m.p. 156–157 °C; ^1^H-NMR (DMSO-*d_6_*, 600 MHz) δ: 7.61 (t, *J* = 7.2 Hz, 2H, Ar*H*), 7.68 (t, *J* =7.2 Hz, 1H, Ar*H*), 7.92–7.96 (m, 3H, Ar*H*), 8.01 (d, *J =* 7.2 Hz, 2H, Ar*H*), 8.19–8.21 (m, 1H, Ar*H*), 9.91 (s, 1H, N*H*). ^13^C-NMR (DMSO-*d_6_*, 150 MHz) δ: 128.2, 128.3, 128.4, 129.3, 130.2, 132.6, 132.8, 133.0, 133.4, 133.5, 134.1, 134.8, 135.2, 148.8, 164.7. MS (ESI^+^): *m*/*z* calcd. for C_15_H_11_NO_4_NaS [M + Na]^+^ 324.02; found 324.03.

*(4-Bromophenyl)(4-hydroxy-1,1-dioxido-2H-benzo[e][1,2]thiazin-3-yl)methanone* (**9b**): Light yellow solid; yield: (304 mg, 80%); m.p. 144–146 °C; ^1^H-NMR (DMSO-*d_6_*, 600 MHz) δ: 7.60 (d, *J* = 7.9 Hz, 3H, Ar*H*), 7.69 (t, *J* = 7.6 Hz, 1H, Ar*H*), 7.93 (d, *J* = 7.5 Hz, 2H, Ar*H*), 8.12–8.17 (m, 1H, Ar*H*), 8.23 (t, *J* = 7.8 Hz, 1H, Ar*H*), 9.93 (s, 1H, N*H*). ^13^C-NMR (DMSO-*d_6_*, 150 MHz) δ: 127.4, 127.7, 128.0, 128.3 (2C), 130.2, 130.6 (2C), 131.8, 132.6, 133.2, 134.7, 136.3, 149.2, 165.7. MS (ESI^+^): *m*/*z* calcd. for C_15_H_10_BrNO_4_NaS [M + Na]^+^ 401.93; found 401.93.

### 3.3. General Procedure for the Synthesis of 2-(3-Benzoyl-4-hydroxy-1,1-dioxido-2H-benzo[e][1,2]thiazin-2-yl)-N-arylacetamides (**11a**–**m**)

3-Benzoyl-1,2-benzothazine (**9a**) (301 mg, 1.0 mmol), 2-bromo-*N*-arylacetamides **10a**–**m** (1.0 mmol), and K_2_CO_3_ (207 mg, 1.5 mmols) were added in DMF (5 mL) and heated for 2-2.5 h at 80-100 °C. Afterward, the reaction mixture was cooled to room temperature and diluted with thrice amount of cold distilled water. Acidification with cold dilute HCl (20%) furnished the colored precipitates of 2-(3-benzoyl-4-hydroxy-1,1-dioxido-2*H*-benzo[*e*][1,2]thiazin-2-yl)-*N*-arylacetamides (**11a**–**m**) (Table 1). Precipitates were filtered, washed with excess distilled water, and finally dried. All the products were obtained in good isolated yields (given below) and were also re-crystallized with methanol.

*2-(3-Benzoyl-4-hydroxy-1,1-dioxido-2H-benzo[e][1,2]thiazin-2-yl)-N-(2-chlorophenyl)acetamide* (**11a**): Light yellow solid; yield: (347 mg, 74%); m.p. 141–142 °C. ^1^H-NMR (DMSO-*d_6_*, 600 MHz) δ: 4.43 (s, 2H, CH_2_), 7.01–7.13 (m, 2H, ArH), 7.19 (dd, *J* = 8.2, 2.0 Hz, 2H, ArH), 7.32–7.39 (m, 1H, ArH), 7.63 (t, *J* = 8.2 Hz, 1H,), 7.90–7.99 (m, 3H, ArH), 8.04 (d, *J* = 7.6 Hz, 2H, ArH), 8.23–8.28 (m, 2H, ArH), 10.03 (s, 1H, NH). ^13^C-NMR (DMSO-*d_6_*, 150 MHz) δ: 53.9, 116.9, 118.6, 119.4, 120.0, 121.7, 122.3, 126.0 (2C), 126.4 (2C), 127.6, 129.0 (2C), 132.6, 133.7, 134.9, 136.2, 138.0, 139.6, 164.9, 167.9, 188.9. MS (ESI^+^): *m*/*z* calcd. for C_23_H_17_ClN_2_O_5_NaS [M + Na]^+^ 491.03; found 491.02.

*2-(3-Benzoyl-4-hydroxy-1,1-dioxido-2H-benzo[e][1,2]thiazin-2-yl)-N-(3-chlorophenyl)acetamide* (**11b**): Light yellow solid; yield: (366 mg, 78%); m.p. 182–184 °C. ^1^H-NMR (DMSO-*d_6_*, 600 MHz) δ: 4.02 (s, 2H, CH_2_), 7.03 (dd, *J* = 7.9, 2.1 Hz, 1H, ArH), 7.12 (dd, *J* = 8.2, 2.0 Hz, 1H, ArH), 7.22 (t, *J* = 8.0 Hz, 1H, ArH), 7.36–7.37 (m, 1H, ArH), 7.63 (t, *J* = 7.5, 2H, ArH), 7.89–7.97 (m, 4H, ArH), 8.06 (d, *J* = 7.7 Hz, 2H, ArH), 8.20–8.22 (m, 1H, ArH), 10.08 (s, 1H, NH). ^13^C-NMR (DMSO-*d_6_*, 150 MHz) δ: 53.7, 117.0, 118.3, 118.8, 119.0, 119.8, 121.9, 123.8, 127.8, 128.7 (3C), 131.6 (2C), 134.2 (2C), 134.9, 135.2, 139.0, 139.9, 165.1, 168.1, 189.1. MS (ESI^+^): *m*/*z* calcd. for C_23_H_17_ClN_2_O_5_NaS [M + Na]^+^ 491.03; found 491.03.

*2-(3-Benzoyl-4-hydroxy-1,1-dioxido-2H-benzo[e][1,2]thiazin-2-yl)-N-(4-chlorophenyl)acetamide* (**11c**): Yellow solid; yield: (347 mg, 73%); m.p. 146-148 °C. ^1^H-NMR (DMSO-*d_6_*, 600 MHz) δ: 4.00 (s, 2H, CH_2_), 7.20–7.26 (m, 4H, ArH), 7.63 (t, 2H, *J* = 7.6 Hz, ArH), 7.70 (t, *J* = 7.3 Hz, 1H, ArH), 7.87–7.90 (m, 1H, ArH), 7.90–7.94 (m, 2H, ArH), 8.05 (d, *J* = 7.6 Hz, 2H, ArH), 8.18–8.19 (m, 1H, ArH), 10.01 (s, 1H, NH). ^13^C-NMR (DMSO-*d_6_*, 150 MHz) δ: 53.8, 116.2, 120.3, 121.8, 126.9, 127.0, 128.4, 128.5 (3C), 128.6 (2C), 128.7 (2C), 132.7, 132.9, 133.8, 135.2, 137.0, 138.5, 164.9, 168.0, 189.1. MS (ESI^+^): *m*/*z* calcd. for C_23_H_17_ClN_2_O_5_NaS [M + Na]^+^ 491.0397; found 491.0295.

*2-(3-Benzoyl-4-hydroxy-1,1-dioxido-2H-benzo[e][1,2]thiazin-2-yl)-N-(o-tolyl)acetamide* (**11d**): Light Brown powder; yield: (359 mg, 80%); m.p. 165-167 °C. ^1^H-NMR (DMSO-*d_6_*, 600 MHz) δ: 2.20 (s, 3H, CH_3_), 3.94 (s, 2H, CH_2_), 6.96 (t, *J* = 7.8 Hz, 2H, ArH), 7.05 (dd, *J* = 7.4, 2.4 Hz, 2H ArH), 7.65 (d, *J* = 7.8 Hz, 3H, ArH), 7.70–7.76 (m, 2H, ArH), 7.87 (d, 1H, ArH), 7.94–8.01 (m, 1H, ArH), 8.04 (t, *J* = 7.9 Hz, 1H, ArH), 8.16–8.21 (m, 1H, ArH), 9.73 (s, 1H, NH). ^13^C-NMR (DMSO-*d_6_*, 150 MHz) δ: 21.2, 54.0, 115.1, 116.6, 119.3, 121.4, 124.2 (2C), 127.6, 128.4 (2C), 128.6 (2C), 128.7, 128.8, 132.2, 132.8, 133.9, 134.4, 137.2, 139.0, 164.4, 167.0, 187.9. MS (ESI^+^): *m*/*z* calcd. for C_24_H_20_N_2_O_5_NaS [M + Na]^+^ 471.09; found 471.09.

*2-(3-Benzoyl-4-hydroxy-1,1-dioxido-2H-benzo[e][1,2]thiazin-2-yl)-N-(m-tolyl)acetamide* (**11e**): Light orange powder; yield: (350 mg, 78%); m.p. 177–178 °C. ^1^H-NMR (DMSO-*d_6_*, 600 MHz) δ: 2.16 (s, 3H, CH_3_), 4.00 (s, 2H, CH_2_), 6.78 (d, *J* = 7.2 Hz, 1H, ArH), 7.02 (d, *J* = 8.3 Hz, 2H, ArH), 7.05 (t, *J* = 7.7 Hz, 2H, ArH), 7.63 (t, *J* = 7.7 Hz, 2H, ArH), 7.70 (t, *J* = 7.4 Hz, 1H, ArH), 7.88–7.93 (m, 2H, ArH), 8.07 (d, *J* = 7.6 Hz, 2H, ArH), 8.19–8.21 (m, 1H, ArH), 9.78 (s, 1H, NH). ^13^C-NMR (DMSO-*d_6_*, 150 MHz) δ: 21.0, 53.7, 115.2, 116.2, 119.4, 121.8, 124.1, 127.1 (2C), 128.4, 128.5, 128.6 (2C), 128.7 (2C), 132.6, 132.9, 133.8, 134.1, 137.0, 138.9, 164.6, 166.3, 187.8. MS (ESI^+^): *m*/*z* calcd. for C_24_H_20_N_2_O_5_NaS [M + Na]^+^ 471.09; found 471.08.

*2-(3-Benzoyl-4-hydroxy-1,1-dioxido-2H-benzo[e][1,2]thiazin-2-yl)-N-(p-tolyl)acetamide* (**11f**): Yellow powder; yield: (341 mg, 76%); m.p. 136-137 °C. ^1^H-NMR (DMSO-*d_6_*, 600 MHz) δ: 2.16 (s, 3H, CH_3_), 3.97 (s, 2H, CH_2_), 6.97 (d, *J* = 8.2 Hz, 2H, ArH), 7.09 (d, *J* = 7.9 Hz, 2H, ArH), 7.63 (t, *J* = 7.2 Hz, 2H, ArH), 7.70 (t, *J* = 7.2 Hz, 1H, ArH), 7.86–7.89 (m, 1H, ArH), 7.90–7.93 (m, 2H, ArH), 8.05 (d, *J* = 7.2 Hz, 2H, ArH), 8.17–8.19 (m, 1H, ArH), 9.77 (s, 1H, NH). ^13^C-NMR (DMSO-*d_6_*, 150 MHz) δ: 20.3, 53.7, 116.2, 118.8, 121.8, 127.0, 128.5, 128.5 (2C), 128.6 (2C), 128.7 (2C), 128.9, 132.3, 132.6, 132.9, 133.8, 135.2, 135.6, 138.6, 164.5, 168.2, 188.9. MS (ESI^+^): *m*/*z* calcd. for C_24_H_20_N_2_O_5_NaS [M + Na]^+^ 471.09; found 471.09.

*2-(3-Benzoyl-4-hydroxy-1,1-dioxido-2H-benzo[e][1,2]thiazin-2-yl)-N-(2-nitrophenyl)acetamide* (**11g**): Light brown solid; yield: (398 mg, 83%); m.p. 157-158 °C. ^1^H-NMR (DMSO-*d_6_*, 600 MHz) δ: 4.06 (s, 2H, CH_2_), 7.23–7.34 (m, 2H, ArH), 7.37 (d, *J* = 8.2 Hz, 2H, ArH), 7.55 (d, *J* = 8.2 Hz, 2H, ArH), 7.53–7.58 (m, 1H, ArH), 7.62 (d, *J* = 7.8 Hz, 1H, ArH), 7.84–7.87 (m, 2H, ArH), 7.88–7.91 (m, 2H, ArH), 8.14–8.12 (m, 1H, ArH), 9.87 (s, 1H, NH). ^13^C-NMR (DMSO-*d_6_*, 150 MHz) δ: 53.8, 113.1, 116.1, 118.0, 121.8, 124.8, 127.2 (2C), 128.3 (2C), 128.7, 128.8, 130.1, 132.6, 132.7, 133.9, 135.3, 138.3, 139.3, 146.9, 165.5, 168.2, 188.9. MS (ESI^+^): *m*/*z* calcd. for C_23_H_17_N_3_O_7_NaS [M + Na]^+^ 502.06; found 502.06.

*2-(3-Benzoyl-4-hydroxy-1,1-dioxido-2H-benzo[e][1,2]thiazin-2-yl)-N-(3-nitrophenyl)acetamide* (**11h**): Light yellow powder; yield: (403 mg, 84%); m.p. 170-172 °C. ^1^H-NMR (DMSO-*d_6_*, 600 MHz) δ: 4.04 (s, 2H, CH_2_), 7.49 (t, *J* = 8.1 Hz, 1H, ArH), 7.56 (d, *J* = 8.1 Hz, 1H, ArH), 7.63 (t, *J* = 7.5 Hz, 2H, ArH), 7.69 (t, *J* = 7.3 Hz, 1H, ArH), 7.84 (dd, *J* = 8.3, 4.2 Hz, 1H, ArH), 7.89–7.97 (m, 3H, ArH), 8.05 (d, *J* = 7.6 Hz, 2H, ArH), 8.09 (s, 1H, ArH), 8.15–8.22 (m, 1H, ArH), 10.38 (s, 1H, NH). ^13^C-NMR (DMSO-*d_6_*, 150 MHz) δ: 53.9, 113.0, 116.2, 117.9, 121.9, 124.9, 127.1, 128.4, 128.6 (2C), 128.7 (2C), 130.1, 132.8, 132.9, 133.9, 135.1, 138.4, 139.1, 147.7, 165.5, 167.9, 189.1. MS (ESI^+^): *m*/*z* calcd. for C_23_H_17_N_3_O_7_NaS [M + Na]^+^ 502.0697; found 502.0531.

*2-(3-Benzoyl-4-hydroxy-1,1-dioxido-2H-benzo[e][1,2]thiazin-2-yl)-N-(4-nitrophenyl)acetamide* (**11i**): Light brown solid; yield: (369 mg, 77%); m.p. 194–195 °C. ^1^H-NMR (DMSO-*d_6_*, 600 MHz) δ: 4.08 (s, 2H, CH_2_), 7.44–7.47 (m, 2H, ArH), 7.63 (td, *J* = 7.2, 1.6 Hz, 2H, ArH), 7.68–7.71 (m, 1H, ArH), 7.89–7.95 (m, 3H, ArH), 8.05 (d, *J* = 7.2 Hz, 2H, ArH), 8.09–8.11 (m, 2H, ArH), 8.20–8.21 (m, 1H, ArH), 10.48 (s, 1H, NH). ^13^C-NMR (DMSO-*d_6_*, 150 MHz) δ: 53.9, 116.1, 118.6 (2C), 121.8, 124.8 (2C), 127.1, 128.3, 128.6 (2C), 128.7 (2C), 132.8, 132.9, 133.9, 135.2, 138.5, 142.2, 144.2, 165.9, 167.7, 189.4. MS (ESI^+^): *m*/*z* calcd. for C_23_H_17_N_3_O_7_NaS [M + Na]^+^ 502.0697; found 502.0675.

*2-(3-Benzoyl-4-hydroxy-1,1-dioxido-2H-benzo[e][1,2]thiazin-2-yl)-N-(3-bromophenyl)acetamide* (**11j**): Yellow solid; yield: (416 mg, 81%); m.p. 163-166 °C. ^1^H-NMR (DMSO-*d_6_*, 600 MHz) δ: 4.04 (s, 2H, CH_2_), 7.04 (d, *J* = 8.5 Hz, 2H, ArH), 7.21 (t, *J* = 7.6 Hz, 2H, ArH), 7.56–7.59 (m, 3H, ArH), 7.68 (t, *J* = 7.5 Hz, 1H, ArH), 7.88–7.90 (m, 3H, ArH), 7.98 (d, *J* = 7.2 Hz, 1H, ArH), 8.12 (s, 1H, ArH), 9.46 (s, 1H, NH). ^13^C-NMR (DMSO-*d_6_*, 150 MHz) δ: 53.9, 116.4, 119.3, 119.7, 122.0, 126.4, 127.3 (2C), 128.3 (3C), 128.5, 130.6, 132.0, 134.5, 136.6 (2C), 138.6, 139.2, 140.5, 165.2, 167.2, 189.2. MS (ESI^+^): *m*/*z* calcd. for C_23_H_17_BrN_2_O_5_NaS [M + Na]^+^ 534.98; found 534.98.

*2-(3-Benzoyl-4-hydroxy-1,1-dioxido-2H-benzo[e][1,2]thiazin-2-yl)-N-(4-bromophenyl)acetamide* (**11k**): Light yellow solid; yield: (421 mg, 82%); m.p. 150-153 °C. ^1^H-NMR (DMSO-*d_6_*, 600 MHz) δ: 4.09 (s, 2H, CH_2_), 7.05 (d, *J* = 7.9 Hz, 2H, ArH), 7.22–7.35 (m, 2H, ArH), 7.56 (d, *J* = 7.9 Hz, 3H, ArH), 7.69–7.74 (m, 2H, ArH), 7.95 (t, *J* = 7.9 Hz, 2H, ArH), 8.09–8.11 (m, 1H, ArH), 8.12–8.14(m, 1H, ArH), 9.48 (s, 1H, NH). ^13^C-NMR (DMSO-*d_6_*, 150 MHz) δ: 54.0, 116.9, 119.4, 119.8, 120.2, 126.4, 126.9 (2C), 127.2 (2C), 128.3 (2C), 128.6, 130.5, 132.1, 134.4, 136.4 (2C), 138.5, 139.9, 140.2, 165.3, 167.4, 188.9. MS (ESI^+^): *m*/*z* calcd. for C_23_H_17_BrN_2_O_5_NaS [M + Na]^+^ 534.98; found 534.97.

*2-(3-Benzoyl-4-hydroxy-1,1-dioxido-2H-benzo[e][1,2]thiazin-2-yl)-N-(2-methoxyphenyl)acetamide* (**11l**): Light brown solid; yield: (372 mg, 80%); m.p. 199-200 °C. ^1^H-NMR (DMSO-*d_6_*, 600 MHz) δ: 3.72 (s, 3H, CH_3_), 4.09 (s, 2H, CH_2_), 6.74 (t, *J* = 7.6 Hz, 1H, ArH), 6.93 (d, *J* = 8.0 Hz, 1H, ArH), 6.98 (t, *J* = 8.1 Hz, 1H, ArH), 7.49 (d, *J* = 7.9 Hz, 1H, ArH), 7.63 (t, *J* = 7.6 Hz, 2H, ArH), 7.70 (t, *J* = 7.4 Hz, 1H, ArH), 7.86–7.91 (m, 3H, ArH), 8.06 (d, *J* = 7.5 Hz, 2H, ArH), 8.16–8.18 (m, 1H, ArH), 9.17 (s, 1H, NH). ^13^C-NMR (DMSO-*d_6_*, 150 MHz) δ: 53.8, 55.5, 111.0, 116.4, 120.0, 121.4, 121.8, 124.4, 126.3, 127.0, 128.5, 128.6 (2C), 128.9 (2C), 132.6, 132.9, 133.7, 135.1, 138.4, 149.2, 165.0, 168.2, 188.8. MS (ESI^+^): *m*/*z* calcd. for C_24_H_20_N_2_O_6_NaS [M + Na]^+^ 487.08; found 487.07.

*2-(3-Benzoyl-4-hydroxy-1,1-dioxido-2H-benzo[e][1,2]thiazin-2-yl)-N-(4-methoxyphenyl)acetamide* (**11m**): Yellow solid; yield: (367 mg, 79%); m.p. 164-165 °C. ^1^H-NMR (DMSO-*d_6_*, 600 MHz) δ: 3.64 (s, 3H, OCH_3_), 3.95 (s, 2H, CH_2_), 6.75 (d, *J* = 7.8 Hz, 2H, ArH), 7.11 (d, *J* = 7.8 Hz, 2H, ArH), 7.63 (t, *J* = 7.2 Hz, 2H, ArH), 7.70 (t, *J* = 7.2 Hz, 1H, ArH), 7.86–7.92 (m, 3H, ArH), 8.04 (d, *J* = 7.2 Hz, 2H, ArH), 8.17 (d, *J* = 7.8 Hz, 1H, ArH), 9.71 (s, 1H, NH). ^13^C-NMR (DMSO-*d_6_*, 150 MHz) δ: 53.7, 55.0, 113.7 (3C), 116.2, 120.4 (3C), 121.8, 127.0, 128.6, 128.7 (2C), 131.2, 132.6, 132.9, 133.7, 135.1, 138.6, 155.2, 164.1, 168.3, 188.8. MS (ESI^+^): *m*/*z* calcd. for C_24_H_20_N_2_O_6_NaS [M + Na]^+^ 487.08; found 487.07.

### 3.4. General Procedure for the Synthesis of 2-(3-(4-Bromobenzoyl)-4-hydroxy-1,1-dioxido-2H-benzo[e][1,2]thiazin-2-yl)-N-arylacetamides (**12a**–**m**)

3-(4-Bromobenzoyl)-1,2-benzothazine (**9b**) (380 mg, 1.0 mmol), 2-bromo-*N*-arylacetamides **10a**–**m** (1.0 mmol), and K_2_CO_3_ (207 mg, 1.5 mmols) were added in DMF (5 mL) and heated for 2-2.5 h at 80–100 °C. Afterward, the reaction mixture was cooled to room temperature and diluted with thrice amount of cold distilled water. Acidification with cold dilute HCl (20%) furnished the colored precipitates of 2-(3**-**(4-bromobenzoyl)-4-hydroxy-1,1-dioxido-2*H*-benzo[*e*][1,2]thiazin-2-yl)-*N*-aryl acetamides (**12a**–**m**) (Table 1). Precipitates were filtered, washed with excess distilled water and then dried. The products were re-crystallized from methanol and were dried.

*2-(3-(4-Bromobenzoyl)-4-hydroxy-1,1-dioxido-2H-benzo[e][1,2]thiazin-2-yl)-N-(2-chlorophenyl)acetamide* (**12a**): Light yellow solid; yield: (438 mg, 80%); m.p. 180-181 °C. ^1^H-NMR (DMSO-*d_6_*, 600 MHz) δ: 4.12 (s, 2H, CH_2_), 7.12 (t, *J* = 7.2 Hz, 1H, ArH), 7.19 (t, *J* = 7.5 Hz, 1H, ArH), 7.34 (d, *J* = 7.8 Hz, 1H, ArH), 7.39 (d, *J* = 7.9 Hz, 1H, ArH), 7.85–7.90 (m, 5H, ArH), 7.96 (d, *J* = 8.1 Hz, 2H, ArH), 8.13–8.15 (m, 1H, ArH), 9.53 (s, 1H, NH), 14.99 (s, 1H, OH_enolic_). ^13^C-NMR (DMSO-*d_6_*, 150 MHz) δ: 53.5, 116.3, 122.0, 125.4, 126.0, 126.3, 126.9, 127.1, 127.3, 128.5, 129.4, 130.7 (2C), 131.8 (2C), 132.7, 133.8, 133.9, 134.3, 138.3, 165.4, 168.1, 187.8. MS (ESI^+^): *m*/*z* calcd. for C_23_H_16_BrClN_2_O_5_NaS [M(^81^Br) + Na]^+^ 570.9497; found 570.9286.

*2-(3-(4-Bromobenzoyl)-4-hydroxy-1,1-dioxido-2H-benzo[e][1,2]thiazin-2-yl)-N-(3-chlorophenyl)acetamide* (**12b**): Yellow powder; yield: (460 mg, 84%); m.p. 148-150 °C. ^1^H-NMR (DMSO-*d_6_*, 600 MHz) δ: 4.00 (s, 2H, CH_2_), 7.12–7.17 (m, 3H, ArH), 7.27 (dd, *J* = 7.8, 2.7 Hz, 1H, ArH), 7.59–7.60 (m, 2H, ArH), 7.74 (d, *J* = 7.6 Hz, 2H, ArH), 7.88–7.95 (m, 3H, ArH), 8.17–8.23 (m, 1H, ArH), 9.55 (s, 1H, NH). ^13^C-NMR (DMSO-*d_6_*, 150 MHz) δ: 53.8, 116.3, 121.9, 126.0, 126.2, 126.2, 126.9, 127.1, 127.4 (2C), 128.3 (2C), 129.8, 130.6 (2C), 132.8, 133.6, 133.9, 134.3, 138.4, 165.1, 168.7, 188.0. MS (ESI^+^): *m*/*z* calcd. for C_23_H_16_BrClN_2_O_5_NaS [M + Na]^+^ 568.94; found 568.94.

*2-(3-(4-Bromobenzoyl)-4-hydroxy-1,1-dioxido-2H-benzo[e][1,2]thiazin-2-yl)-N-(4-chlorophenyl)acetamide* (**12c**): Yellow solid; yield: (477 mg, 87%); m.p. 181-184 °C. ^1^H-NMR (DMSO-*d_6_*, 600 MHz) δ: 4.08 (s, 2H, CH_2_), 7.24–7.30 (m, 1H, ArH), 7.44–7.48 (m, 2H, ArH), 7.54–7.57 (m, 2H, ArH), 7.74 (d, *J* = 7.6 Hz, 1H, ArH), 7.88–7.95 (m, 3H, ArH), 8.08–8.13 (m, 2H, ArH), 8.19–8.21 (m, 1H, ArH), 9.59 (s, 1H, NH). ^13^C-NMR (DMSO-*d_6_*, 150 MHz) δ: 54.0, 116.8, 116.1, 118.6 (2C), 124.9 (2C), 126.3 (2C), 126.8, 127.1 (2C), 128.2 (2C), 129.8, 132.8, 133.7, 133.9, 136.4, 138.5, 165.0, 167.4, 188.3. MS (ESI^+^): *m*/*z* calcd. for C_23_H_16_BrClN_2_O_5_NaS [M + Na]^+^ 568.94; found 568.93.

*2-(3-(4-Bromobenzoyl)-4-hydroxy-1,1-dioxido-2H-benzo[e][1,2]thiazin-2-yl)-N-(o-tolyl)acetamide* (**12d**): Light orange powder; yield: (464 mg, 88%); m.p. 193-194 °C. ^1^H-NMR (DMSO-*d_6_*, 600 MHz) δ: 1.96 (s, 3H, CH_3_), 4.07 (s, 2H, CH_2_), 6.98–7.02 (m, 3H, ArH), 7.10 (d, *J* = 6.9 Hz, 1H, ArH), 7.86–7.92 (m, 5H, ArH), 7.98 (d, *J* = 8.2 Hz, 2H, ArH), 8.13–8.15 (m, 1H, ArH), 9.29 (s, 1H, NH). ^13^C-NMR (DMSO-*d_6_*, 150 MHz) δ: 17.4, 53.5, 116.3, 121.9, 124.5, 125.3, 125.8, 127.0, 127.5, 128.5, 130.2, 130.7 (2C), 131.5, 131.8 (2C), 132.7, 133.8, 134.2, 135.2, 138.4, 164.9, 168.3, 187.2. MS (ESI^+^): *m*/*z* calcd. for C_24_H_19_BrN_2_O_5_NaS [M + Na]^+^ 551.0097; found 550.9858.

*2-(3-(4-Bromobenzoyl)-4-hydroxy-1,1-dioxido-2H-benzo[e][1,2]thiazin-2-yl)-N-(m-tolyl)acetamide* (**12e**): Light yellow solid; yield: (427 mg, 81%); m.p. 143-144 °C. ^1^H-NMR (DMSO-*d_6_*, 600 MHz) δ: 2.17 (s, 3H, CH_3_), 3.99 (s, 2H, CH_2_), 6.79 (d, *J* = 7.3 Hz, 1H, ArH), 7.01 (d, *J* = 8.9 Hz, 2H, ArH), 7.06 (t, *J* = 7.6 Hz, 1H, ArH), 7.83–7.95 (m, 7H, ArH), 8.16–8.19 (m, 1H, ArH), 9.37 (s, 1H, NH), 14.92 (s, 1H, O*H*enolic). ^13^C-NMR (DMSO-*d_6_*, 150 MHz) δ: 21.0, 53.9, 116.1, 116.3, 119.3, 122.0, 124.1, 126.9, 127.1, 128.4 (2C), 130.7 (2C), 131.7 (2C), 132.8, 133.9, 134.3, 137.8, 138.0, 138.4, 164.6, 168.0, 187.6. MS (ESI^+^): *m*/*z* calcd. for C_24_H_19_BrN_2_O_5_NaS [M + Na]^+^ 549.0097; found 550.0000.

*2-(3-(4-Bromobenzoyl)-4-hydroxy-1,1-dioxido-2H-benzo[e][1,2]thiazin-2-yl)-N-(p-tolyl)acetamide* (**12f**): Light brown solid; yield: (454 mg, 86%); m.p. 190-192 °C. ^1^H-NMR (DMSO-*d_6_*, 600 MHz) δ: 2.43 (s, 3H, CH_3_), 3.98 (s, 2H, CH_2_), 6.98 (d, *J* = 8.3 Hz, 2H, ArH), 7.09 (d, *J* = 7.9 Hz, 1H, ArH), 7.70 (t, *J* = 7.2 Hz, 1H, ArH), 7.74 (t, *J* = 7.2 Hz, 2H, ArH), 7.87–7.90 (m, 1H, ArH), 7.91–7.94 (m, 2H, ArH), 8.05 (d, *J* = 7.2 Hz, 2H, ArH), 8.17–8.19 (m, 1H, ArH), 9.48 (s, 1H, NH). ^13^C-NMR (DMSO-*d_6_*, 150 MHz) δ: 21.0, 53.8, 116.2, 118.8, 121.8, 127.0, 128.3 (2C), 128.5, 128.6 (2C), 128.7 (2C), 128.9, 132.3, 132.6, 132.9, 133.8, 135.2, 135.6, 138.6, 164.4, 168.1, 188.0. MS (ESI^+^): *m*/*z* calcd. for C_24_H_19_BrN_2_O_5_NaS [M + Na]^+^ 549.00; found 549.01.

*2-(3-(4-Bromobenzoyl)-4-hydroxy-1,1-dioxido-2H-benzo[e][1,2]thiazin-2-yl)-N-(2-nitrophenyl)acetamide* (**12g**): Light brown powder; yield: (497 mg, 89%); m.p. 177–178 °C. ^1^H-NMR (DMSO-*d_6_*, 600 MHz) δ: 4.08 (s, 2H, CH_2_)_,_ 7.29 (q, *J* = 7.2, 1H, ArH), 7.40 (t, *J* = 8.4, 1H, ArH), 7.58 (q, *J* = 7.2 Hz, 1H, ArH), 7.83–7.90 (m, 6H, ArH), 7.98 (t, *J* = 8.4 Hz, 2H, ArH), 8.11–8.15 (m, 1H, ArH), 10.17 (s, 1H, NH). ^13^C-NMR (DMSO-*d_6_*, 150 MHz) δ: 53.7, 116.2, 122.0, 124.8, 125.1, 125.4, 126.9, 127.2 (2C), 128.4, 130.2, 130.6 (2C), 131.8 (2C), 132.8, 133.9, 134.0, 134.5, 138.2, 165.5, 168.3, 187.7. MS (ESI^+^): *m*/*z* calcd. for C_23_H_16_BrN_3_O_7_NaS [M + Na]^+^ 579.97; found 579.98.

*2-(3-(4-Bromobenzoyl)-4-hydroxy-1,1-dioxido-2H-benzo[e][1,2]thiazin-2-yl)-N-(3-nitrophenyl)acetamide* (**12h**): Light yellow solid; yield: (469 mg, 84%); m.p. 208-209 °C. ^1^H-NMR (DMSO-*d_6_*, 600 MHz) δ: 4.05 (s, 2H, CH_2_), 7.50 (t, *J* = 7.8 Hz, 1H, ArH), 7.58 (dd, *J* = 6.0, 2.0 Hz, 1H, ArH), 7.82–7.87 (m, 3H, ArH), 7.91–8.07 (m, 5H, ArH), 8.16–8.17 (m, 1H, ArH), 8.19–8.21 (m, 1H, ArH), 10.39 (s, 1H, NH). ^13^C-NMR (DMSO-*d_6_*, 150 MHz) δ: 54.1, 112.9, 116.3, 118.0, 126.2, 126.5, 127.4 (2C), 127.9 (2C), 128.4 (2C), 130.0, 131.7, 132.9, 134.0, 134.2, 138.2, 139.1, 147.7, 165.5, 167.9, 188.1. MS (ESI^+^): *m*/*z* calcd. for C_23_H_16_BrN_3_O_7_NaS [M + Na]^+^ 579.97; found 579.98.

*2-(3-(4-Bromobenzoyl)-4-hydroxy-1,1-dioxido-2H-benzo[e][1,2]thiazin-2-yl)-N-(4-nitrophenyl)acetamide* (**12i**): Yellow powder; yield: (491 mg, 88%); m.p. 210-212 °C. ^1^H-NMR (DMSO-*d_6_*, 600 MHz) δ: 4.08 (s, 2H, CH_2_), 7.47 (d, *J* = 7.8 Hz, 1H, ArH), 7.84 (d, *J* = 7.8 Hz, 2H, ArH), 7.88–7.95 (m, 5H, ArH), 8.11 (d, *J* = 7.8 Hz, 3H, ArH), 8.19 (d, *J* = 7.0 Hz, 1H, ArH), 10.04 (s, 1H, NH), 15.18 (s, 1H, OH_enolic_). ^13^C-NMR (DMSO-*d_6_*, 150 MHz), δ: 53.8, 114.1, 116.2, 118.5, 122.0, 124.9, 127.2 (2C), 130.0 (2C), 130.2, 131.5, 131.7, 132.9, 133.9, 136.9, 138.4, 142.2, 144.2, 148.2, 165.9, 167.9, 188.7. MS (ESI^+^): *m*/*z* calcd. for C_23_H_16_BrN_3_O_7_NaS [M + Na]^+^ 579.97; found 579.97.

*2-(3-(4-Bromobenzoyl)-4-hydroxy-1,1-dioxido-2H-benzo[e][1,2]thiazin-2-yl)-N-(3-bromophenyl)acetamide* (**12j**): Light orange solid; yield: (527 mg, 89%); m.p. 145–147 °C. ^1^H-NMR (DMSO-*d_6_*, 600 MHz) δ: 4.00 (s, 2H, CH_2_), 7.13–7.17 (m, 3H, ArH), 7.48 (s, 1H, ArH), 7.55–7.59 (m, 3H, ArH), 7.84 (d, *J* = 7.8 Hz, 2H, ArH), 7.88–7.94 (m, 2H, ArH), 8.17–8.19 (m, 1H, ArH), 10.05 (s, 1H, NH), 14.97 (s, 1H, OH_enolic_). ^13^C-NMR (DMSO-*d_6_*, 150 MHz) δ: 54.0, 116.3, 117.6, 121.1, 121.3 (2C), 122.0, 126.0, 127.2 (2C), 130.7 (2C), 130.7 (3C), 131.7 (2C), 132.8, 133.9, 138.3, 139.6, 165.4, 168.7, 186.9. MS (ESI^+^): *m*/*z* calcd. for C_23_H_16_Br_2_N_2_O_5_NaS [M + Na]^+^ 612.89; found 612.89.

*2-(3-(4-Bromobenzoyl)-4-hydroxy-1,1-dioxido-2H-benzo[e][1,2]thiazin-2-yl)-N-(4-bromophenyl)acetamide* (**12k**): Yellow solid; yield: (545 mg, 92%); m.p. 200-203 °C. ^1^H-NMR (DMSO-*d_6_*, 600 MHz) δ: 4.00 (s, 2H, CH_2_), 7.19 (d, *J* = 8.0 Hz, 3H, ArH), 7.37 (dt, *J* = 9.0, 3.0 Hz, 2H, ArH), 7.83–7.94 (m, 3H, ArH), 8.16–8.18 (m, 3H, ArH), 10.12 (s, 1H, NH). ^13^C-NMR (DMSO-*d_6_*, 150 MHz) δ: 54.0, 114.9, 116.2, 120.7 (3C), 121.1, 127.9, 130.7 (3C), 131.4 (3C), 131.7 (2C), 132.8, 133.9, 137.4, 138.0, 165.2, 167.9, 187.0. MS (ESI^+^): *m*/*z* calcd. for C_23_H_16_Br_2_N_2_O_5_NaS [M + Na]^+^ 612.89; found 612.88.

*2-(3-(4-Bromobenzoyl)-4-hydroxy-1,1-dioxido-2H-benzo[e][1,2]thiazin-2-yl)-N-(2-methoxyphenyl)acetamide* (**12l**): Yellow solid; yield: (484 mg, 89%); m.p. 186–187 °C. ^1^H-NMR (DMSO-*d_6_*, 600 MHz) δ: 3.73 (s, 3H, OCH_3_), 4.11 (s, 2H, CH_2_), 6.75 (t, *J* = 7.8 Hz, 1H, ArH), 6.94 (dd, *J* = 7.8, 1.4 Hz, 1H, ArH), 6.98 (t, *J* = 7.8 Hz, 1H, ArH), 7.51 (d, *J* = 7.8 Hz, 1H, ArH), 7.85 (d, *J* = 7.8 Hz, 1H, ArH), 7.86–7.91 (m, 4H, ArH), 7.95 (d, *J* = 7.8 Hz, 2H, ArH), 8.15–8.18 (m, 1H, ArH), 9.17 (s, 1H, NH), 14.92 (s, 1H, OH_enolic_). ^13^C-NMR (DMSO-*d_6_*, 150 MHz) δ: 53.7, 55.1, 111.7, 116.2, 120.4 (2C), 121.3, 121.8, 124.6, 126.5, 127.0, 127.6, 128.7 (2C), 131.2, 132.6, 132.9, 133.7, 135.1, 138.6, 149.2, 164.9, 167.6, 187.8. MS (ESI^+^): *m*/*z* calcd. for C_24_H_19_BrN_2_O_6_NaS [M + Na]^+^ 564.99; found 564.98.

*2-(3-(4-Bromobenzoyl)-4-hydroxy-1,1-dioxido-2H-benzo[e][1,2]thiazin-2-yl)-N-(4-methoxyphenyl)acetamide* (**12m**): Yellow powder; yield: (489 mg, 90%); m.p. 198-199 °C. ^1^H NMR (DMSO-*d_6_*, 600 MHz) δ: 3.73 (s, 3H, OCH_3_), 4.01 (s, 2H, CH_2_), 6.75 (t, *J* = 7.8 Hz, 1H, ArH), 6.93 (dd, *J* = 7.8, 1.4 Hz, 1H, ArH), 6.98 (t, *J* = 7.8 Hz, 1H, ArH), 7.50 (d, *J* = 7.8 Hz, 1H, ArH), 7.84 (d, *J* = 7.8 Hz, 1H, ArH), 7.86–7.91 (m, 4H, ArH), 7.96 (d, *J* = 7.8 Hz, 2H, ArH), 8.15–8.18 (m, 1H, ArH), 9.17 (s, 1H, NH), 14.92 (s, 1H, OH_enolic_). ^13^C-NMR (DMSO-*d_6_*, 150 MHz) δ: 54.0, 55.5, 111.0, 116.4, 120.0, 121.2, 121.9, 124.4, 126.4, 126.9, 127.1, 128.4, 130.7 (2C), 131.7 (2C), 132.7, 133.8, 134.3, 138.4, 149.1, 165.0, 168.0, 187.8. MS (ESI^+^): *m*/*z* calcd. for C_24_H_19_BrN_2_O_6_NaS [M + Na]^+^ 566.99; found 564.98.

### 3.5. In Silico α-Glucosidase Inhibition

The Molecular Operating Environment (MOE) Ver.2014.0901 software [66,67,68] was utilized for the in silico analysis of all the 1,2-benzothiazine derivatives along with the reference acarbose as follows.

#### 3.5.1. Ligand Preparation

Structures of all these compounds were designed with the help of ChemDraw Ultra 12.02. In order to display these files in the MOE, structures of these compounds were saved in an MDL file (“.sdf”). The 2D structure of the reference acarbose in the SDF file was retrieved from NCBI Pubchem. After this, 3D protonation and energy minimizations of all these compounds along with the reference acarbose were executed under default parameters in the MOE.

#### 3.5.2. Protein Preparation

The 3D structure of *Saccharomyces cerevisiae* α-glucosidase (PDB ID: 2QMJ) was downloaded from the Protein Data Bank (http://www.rcsb.org/pdb; accessed on 10 October 2020). Binding interactions of the ligand (acarbose) with the protein residues were checked (Asp203, Asp542, Asp327, His600, Arg526) and then the ligand, solvent molecules, and unknown atoms from the receptor protein were removed. With the help of the MOE site finder module, the active site from the 3D structure of the protein was computed. The ligand site was found surrounded by Asp203, Tyr299, Asp327, Ile328, Asp366, Trp406, Trp441, Asp443, Met444, Arg526, Trp539, Gly541, Asp542, Asp571, Phe575, Arg598, and His600 residues. Later on, the site was occupied by dummy atoms and the protein was 3D-protonated and energy minimized with default parameters set in the MOE software (Ver.2014.0901).

#### 3.5.3. Molecular Docking

To access the binding modes of all the ligands with α-glucosidase enzymes, ten conformations of all the ligands along with acarbose were docked into the selected site of enzymes with the help of the MOE dock module under the parameters fixed as, placement: triangle matcher, rescoring: London dG, refinement: forcefield and retain: 10. Finally, 2D and 3D maps of ligands were obtained. Binding modes of ligands were studied using rmsd values and docking scores of their top-ranked conformations. Results of molecular docking are depicted in Table 1, Table S1, and Figure 2, Figure 3, and Figure S1.

### 3.6. In Vitro α-Glucosidase Inhibition

A general spectrophotometric technique was used to perform the α-glucosidase inhibition assay with slight modifications [69]. All the compounds were dissolved in a dimethyl sulfoxide (DMSO) solvent while α-glucosidase enzymes (obtained from *Saccharomyces cerevisiae* (Sigma-Aldrich, Munich, Germany) were dissolved in a phosphate buffer (pH 6.8, 100 mM). Twelve and a half microliters of each test compound (dissolved in DMSO), 40 μL of enzymes (0.5 U/mL), and 120 μL of phosphate buffer (100 mM) were added in 96-well microliter plates. The plates were incubated for 5 min at 37 °C. After incubation, 40 μL of 5 mM *p*-nitrophenyl-α-D-glucopyranoside (Sigma Aldrich, Munich, Germany) was added in each sample solution and incubated for a further 30 min. Afterward, absorbance of released 4-nitrophenol was measured at 405 nm and 37 °C using a microplate reader. DMSO and acarbose (Sigma-Aldrich, Munich, Germany) were used as a negative control and reference inhibitor, respectively. The experiment was repeated thrice and inhibition percentages were calculated using the following formula.

% Inhibition = [(A _control_ − A _sample_) / A _control_] × 100 (1)

where ‘A’ stands for absorbance.

Finally, IC_50_ values were also determined for the compounds having 50% or above inhibition values.

## 4. Conclusions

Novel 1,2-benzothiazine-*N*-arylacetamides have been discovered as inhibitors of α-glucosidase enzymes for the first time. The 1,2-benzothiazine-*N*-arylacetamides **12a**–**m** proved better inhibitors compared to derivatives **11a**–**m**. The compounds **11c**, **11h**, **11i**, **12a**, **12d**, **12e**, **12g**, and **12m** exhibited low IC_50_ values compared to acarbose (IC_50_ = 58.8 μM). However, the derivatives **11c**, **12a**, **12d**, **12e**, and **12g** were envisioned as the most potent inhibitors as these templates showed good docking scores, low rmsd values and significant interactions with the targeted receptor pockets during in silico experiments. Theses derivatives also showed low IC_50_ values of 30.65, 18.25, 20.76, 35.14, and 24.24 μM, respectively, during in vitro α-glucosidase inhibition. We hope the present work will benefit researchers to develop more active antidiabetic agents with no side effects.

## Figures and Tables

**Table 1:** Structural parameters, in silico (docking scores, binding residues, and rmsd values) and in vitro (IC_50_ values) inhibitory potential of 1,2-benzothiazines **9a**,**b**, 1,2-benzothiazine-*N*-arylacetamides **11a**–**m**, and **12a**–**m** against α-glucosidase enzymes.

Compound	R_2_	Docking Scores (Kcal/mol)	Binding Residues	Rmsd Values (Å)	Inhibitions (%)	IC_50_ (μM)
**9a**	-	−9.12	-	2.54	20	ND
**9b**	-	−10.11	-	2.14	55	> 100
**11a**	2-Cl	−11.36	Met444	2.03	39	ND
**11b**	3-Cl	−10.33	-	1.50	32	ND
**11c**	4-Cl	−12.80	*Asp327*, Asp474, Met444	1.08	79	30.65
**11d**	2-Me	−11.02	Thr205	2.33	10	ND
**11e**	3-Me	−11.42	-	1.82	15	ND
**11f**	4-Me	−10.76	-	1.72	37	ND
**11g**	2-NO_2_	−11.85	*Asp203*	1.32	58	88.12
**11h**	3-NO_2_	−12.13	*Asp542*, Thr205	1.21	63	47.13
**11i**	4-NO_2_	−12.25	*Asp542*	1.11	77	40.41
**11j**	3-Br	−11.83	Thr205	1.32	50	> 100
**11k**	4-Br	−10.23	-	1.44	13	ND
**11l**	2-OMe	−11.77	-	1.39	54	> 100
**11m**	4-OMe	−11.85	-	1.32	69	88.12
**12a**	2-Cl	−14.23	*Arg526*, *Asp542*, *Asp327*, Trp406	1.21	79	18.25
**12b**	3-Cl	−10.18	Phe575	1.48	47	> 100
**12c**	4-Cl	−10.95	-	1.81	61	> 100
**12d**	2-Me	−13.91	*Asp203*, *Asp327*	1.13	76	20.76
**12e**	3-Me	−13.47	*Asp203*, *Asp327*	1.27	71	35.14
**12f**	4-Me	−10.86	Thr544	1.09	20	ND
**12g**	2-NO_2_	−13.52	*Asp203*, *Asp327*	1.01	76	24.24
**12h**	3-NO_2_	−12.25	-	1.41	58	> 100
**12i**	4-NO_2_	−10.82	-	1.27	33	ND
**12j**	3-Br	−11.94	-	1.71	12	ND
**12k**	4-Br	−11.92	*Asp327*, Tyr605	1.93	48	96.25
**12l**	2-OMe	−11.60	Met444, Phe575	1.23	34	ND
**12m**	4-OMe	−12.32	*Asp327*	1.19	65	58.45
**Acarbose**	-	−16.18	*His600*, *Asp542*, *Arg526*, *Asp327*, Met444, Lys480	2.00	45	58.8

ND = not determined; acarbose was used as reference drug; inhibition percentages were determined at 200 μM concentration; reference (selected) residues are in bold and *italic* form.

## Data Availability

he data presented in this study are available in the Supplementary Materials.

## Supplementary Materials

The following are available online: ^1^H-NMR, ^13^C-NMR, and MS spectra of potent α-glucosidase inhibitors. Molecular docking 2D/3D maps (Figure S1, Table S1) of compounds **9a**, **9b**, **11**(**a**,**b**,**d**–**m**)**,** and **12**(**b**,**c**,**f**,**h**–**m**).

## Sample Availability

Samples of the compounds are available from the authors.
