# Supplementary material for: Synthesis and α-Glucosidase Inhibition Activity of 2-[3-(Benzoyl/4-bromobenzoyl)-4-hydroxy-1,1-dioxido-2H-benzo[e][1,2]thiazin-2-yl]-N-arylacetamides: An In Silico and Biochemical Approach
Source: Molecules. 2021 May 20;26(10):3043. doi: 10.3390/molecules26103043 (PMC8161055; doi:10.3390/molecules26103043)
Supplement: Supplementary file 1 [file molecules-26-03043-s001.zip › molecules-1190984-SI.pdf]

molecules

Supplementary Materials

Synthesis and α-Glucosidase Inhibition Activity of 2-[3-(Benzoyl/4-bromobenzoyl)-4-hydroxy-1,1-dioxido-2*H*-benzo[*e*][1,2]thiazin-2-yl]-*N*-arylacetamides: An *In Silico* and Biochemical Approach

Furqan Ahmad Saddique, Sana Aslam, Matloob Ahmad *, Usman Ali Ashfaq, Muhammad Muddassar, Sadia Sultan, Saman Taj, Muzammil Hussain, Dae Sung Lee and Magdi E. A. Zaki *

(Supplementary Data File)

[1]*H* NMR, [13]*C* NMR and MS Spectra of potent compounds

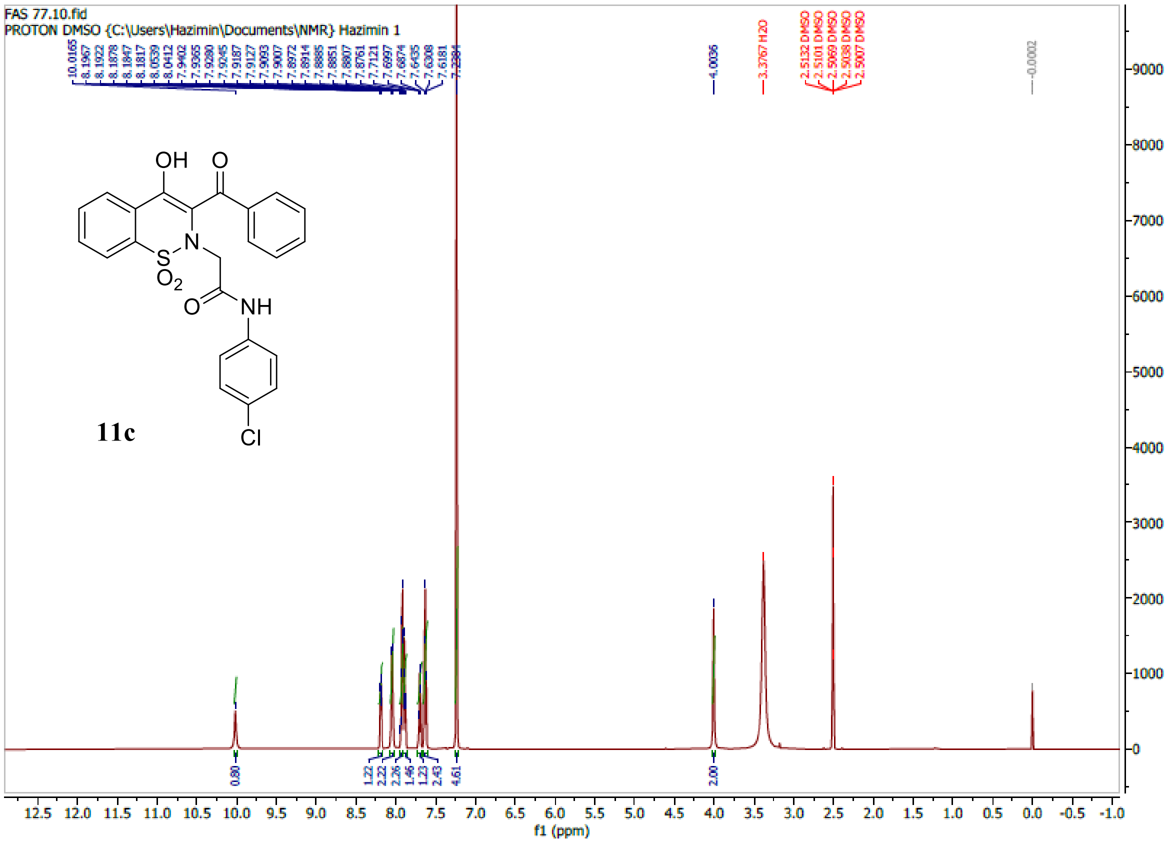

[1]*H* NMR Spectrum of compound **11c**.

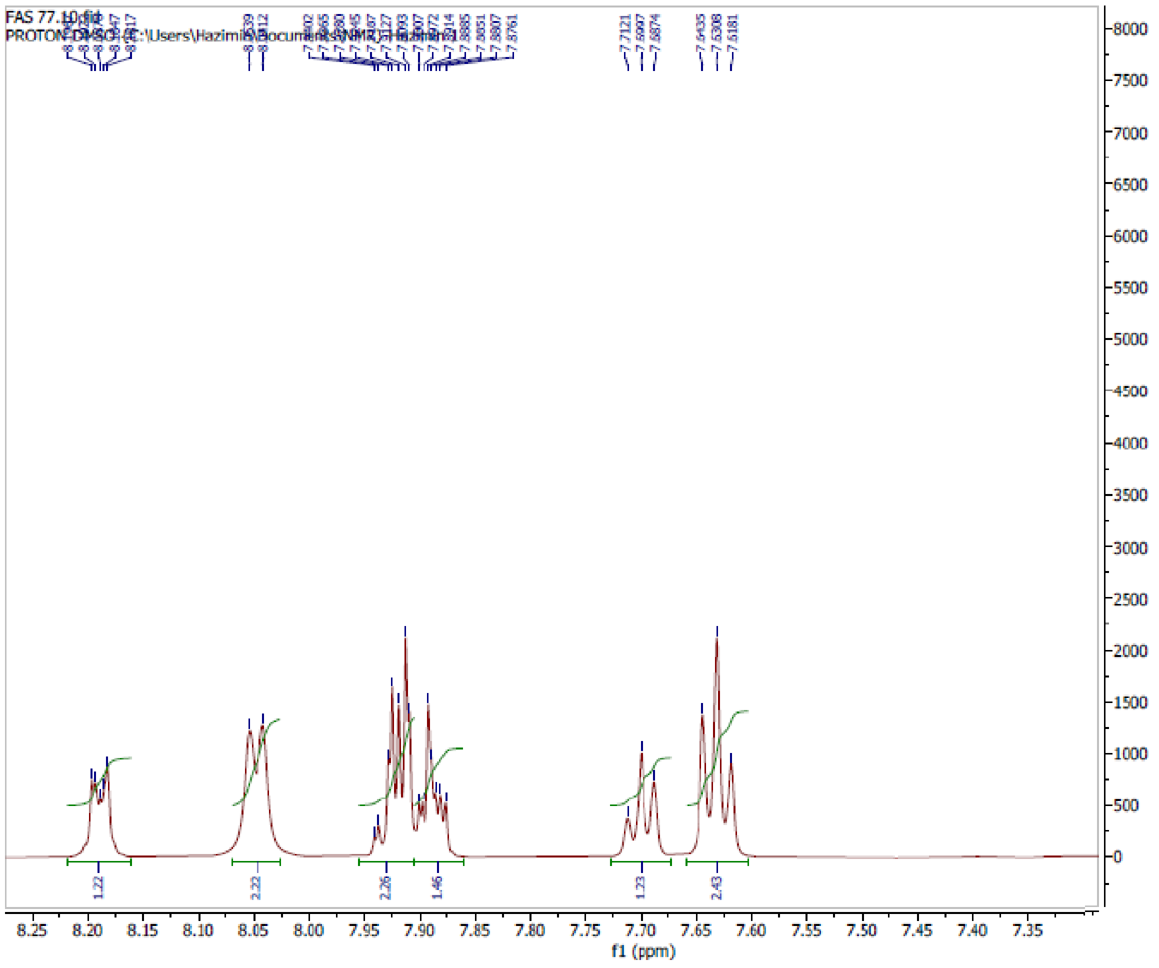

Expanded form of ^{1}H NMR Spectrum of compound **11c** (region 7.60-8.25 ppm).

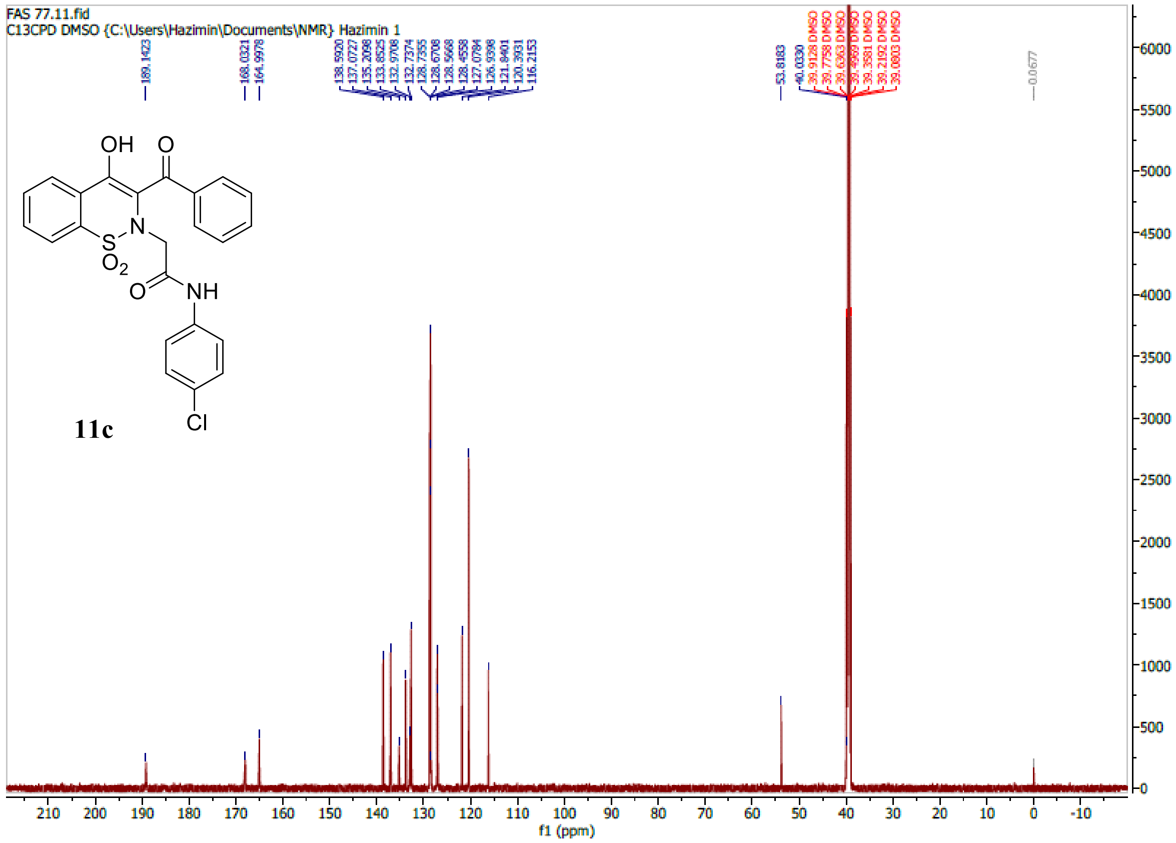

^{13}C NMR Spectrum of compound **11c.**

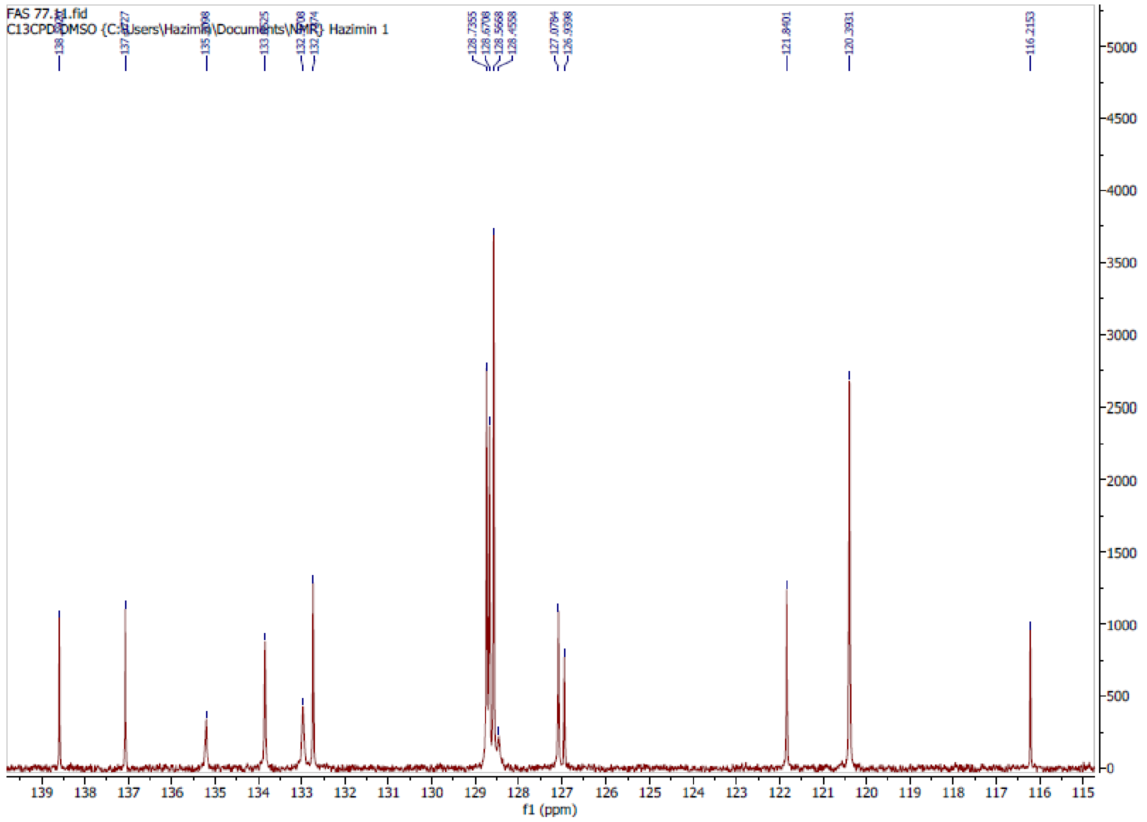

Expanded form of ^{13}C NMR Spectrum of compound **11c** (region 116-139 ppm).

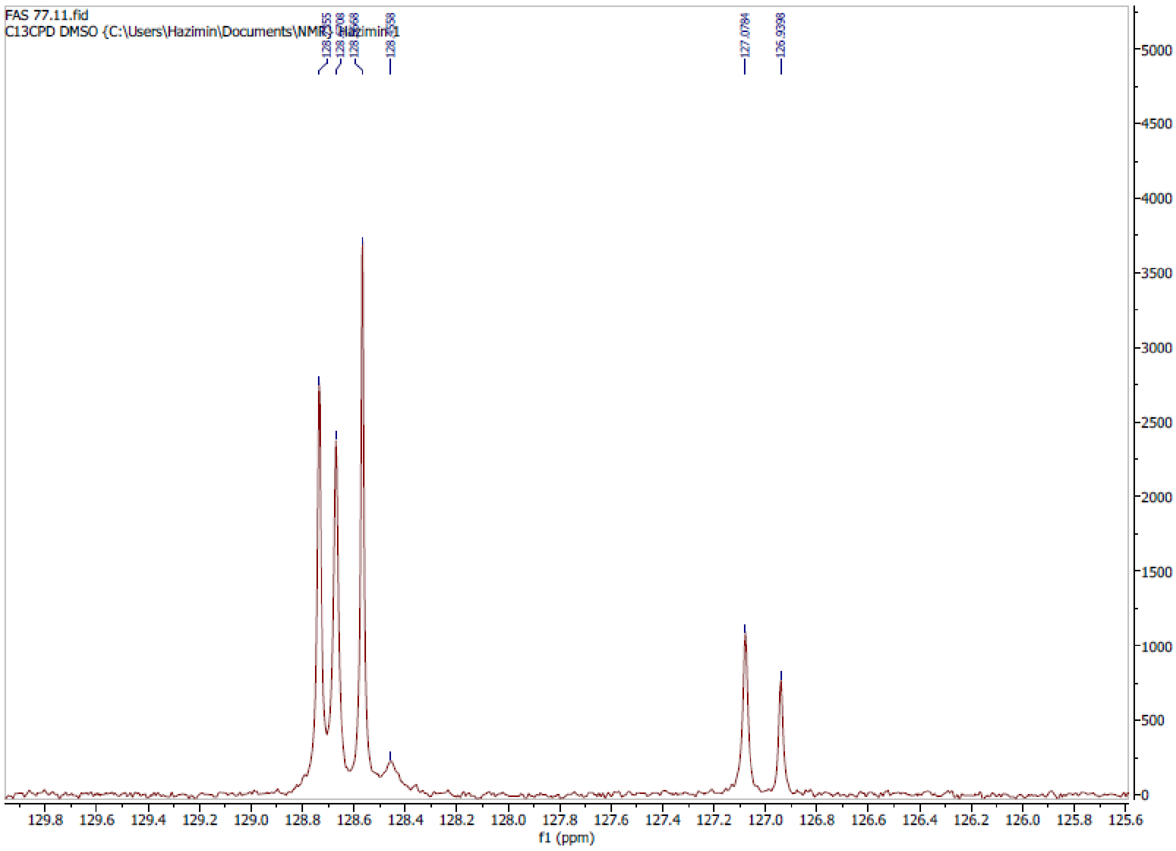

Expanded form of ^{13}C NMR Spectrum of compound **11c** (region 126.8-129.0 ppm).

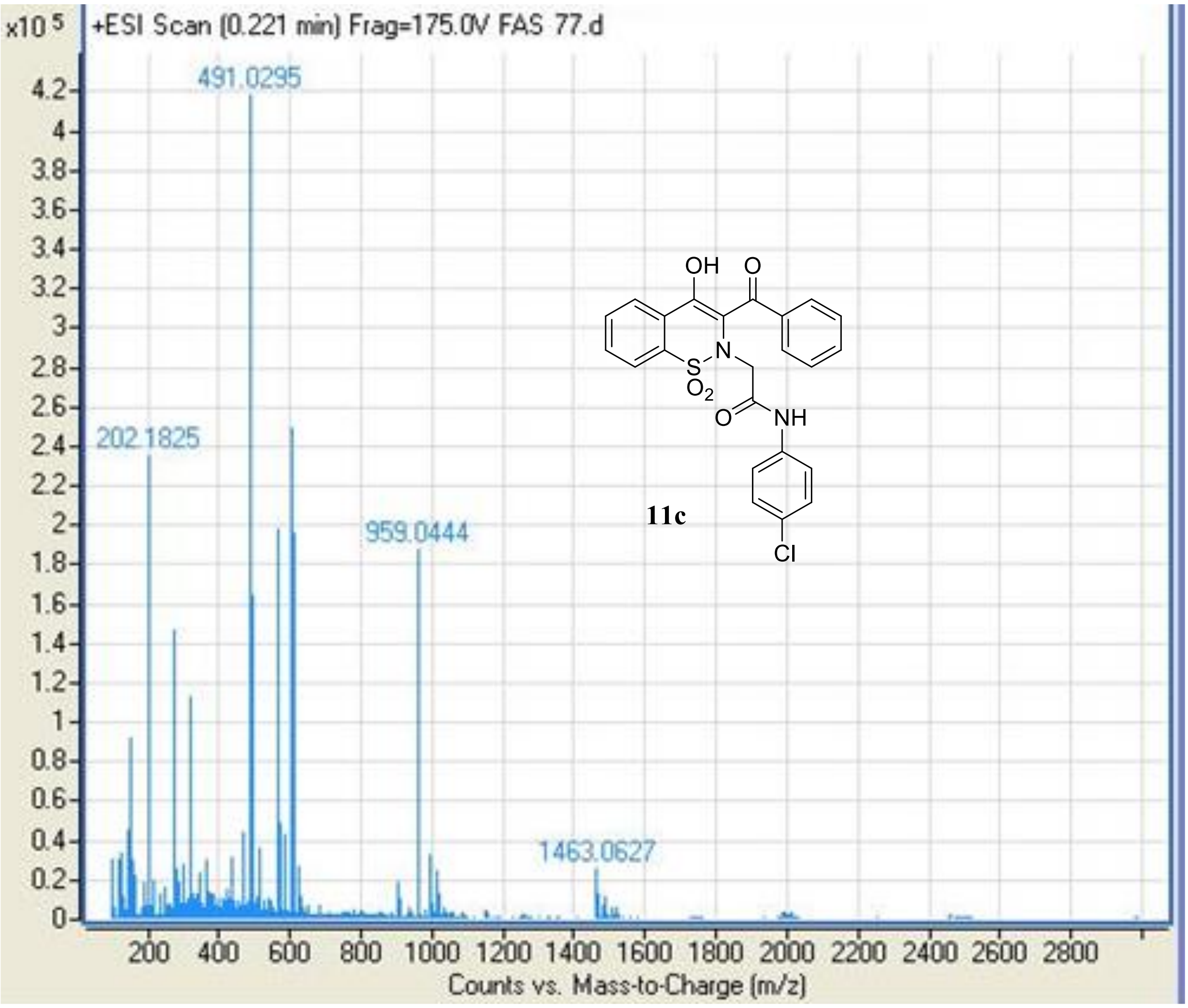

MS Spectrum of compound **11c.**

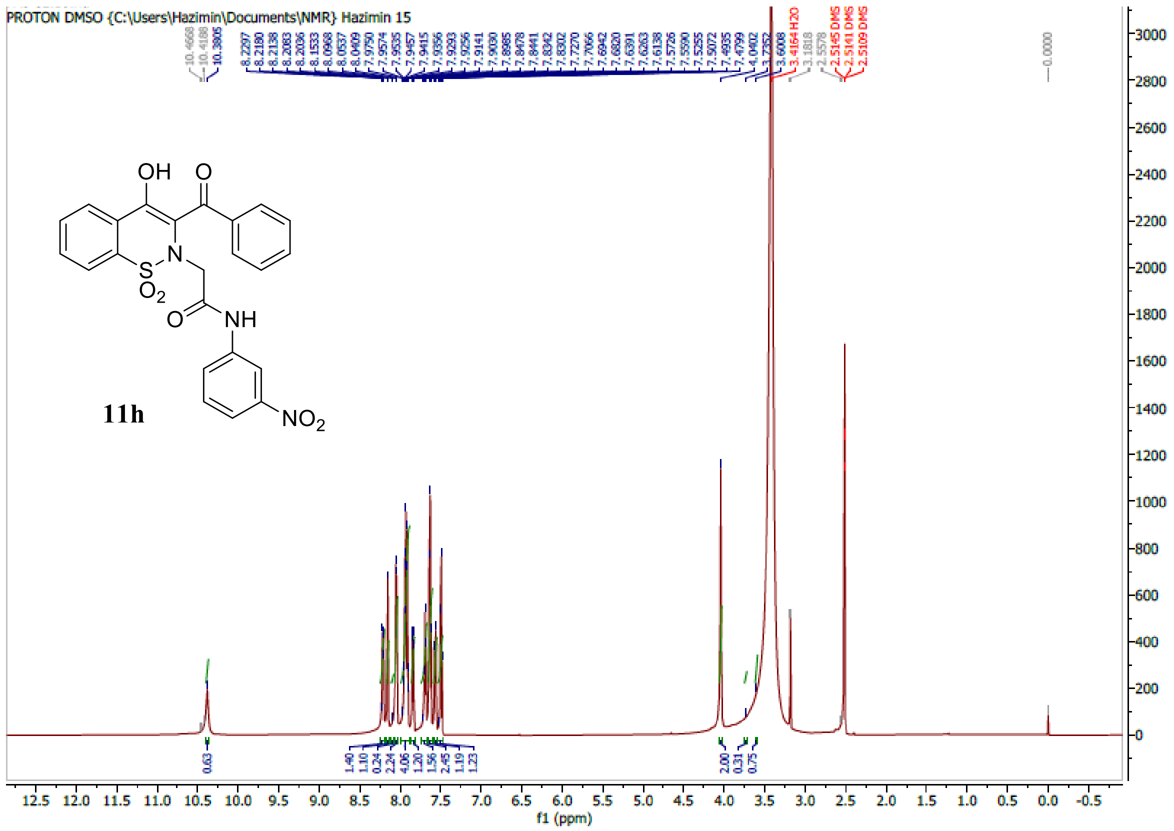

[1]*H* NMR Spectrum of compound **11h**.

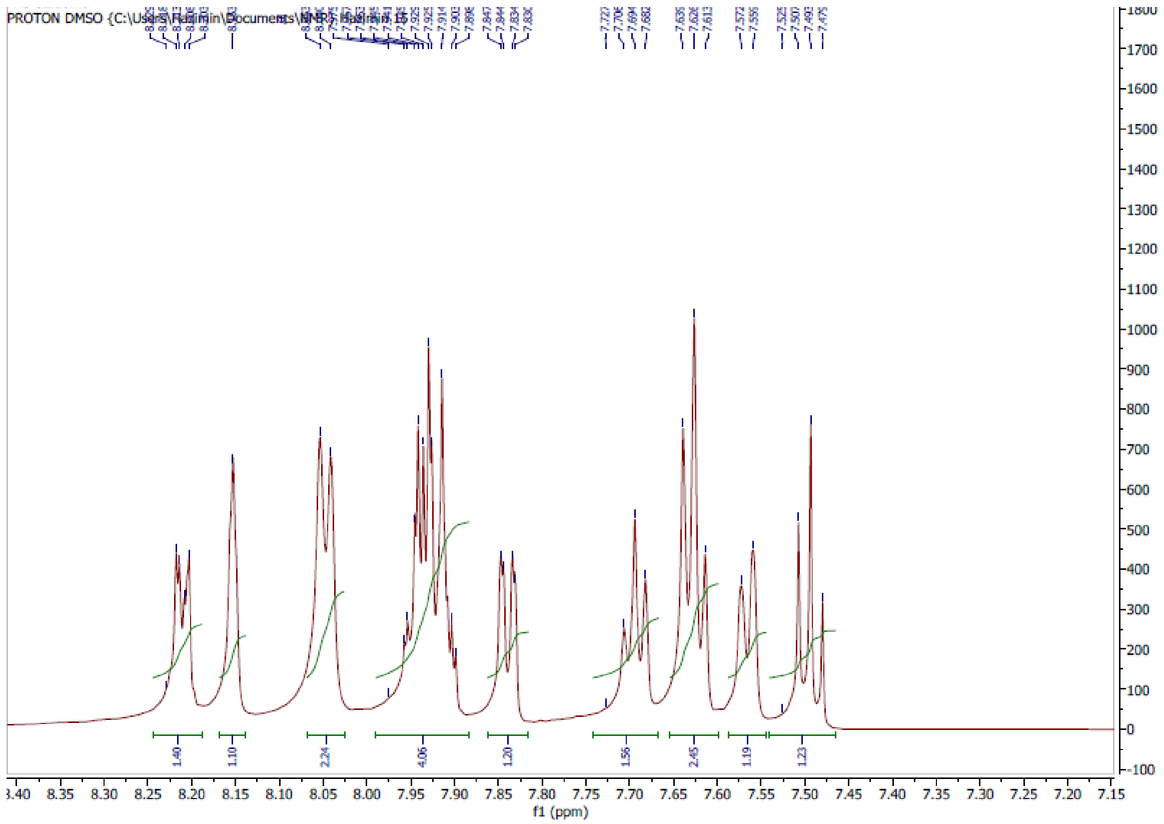

Expanded form of [1]*H* NMR Spectrum of compound **11h** (region 7.45-8.25 ppm).

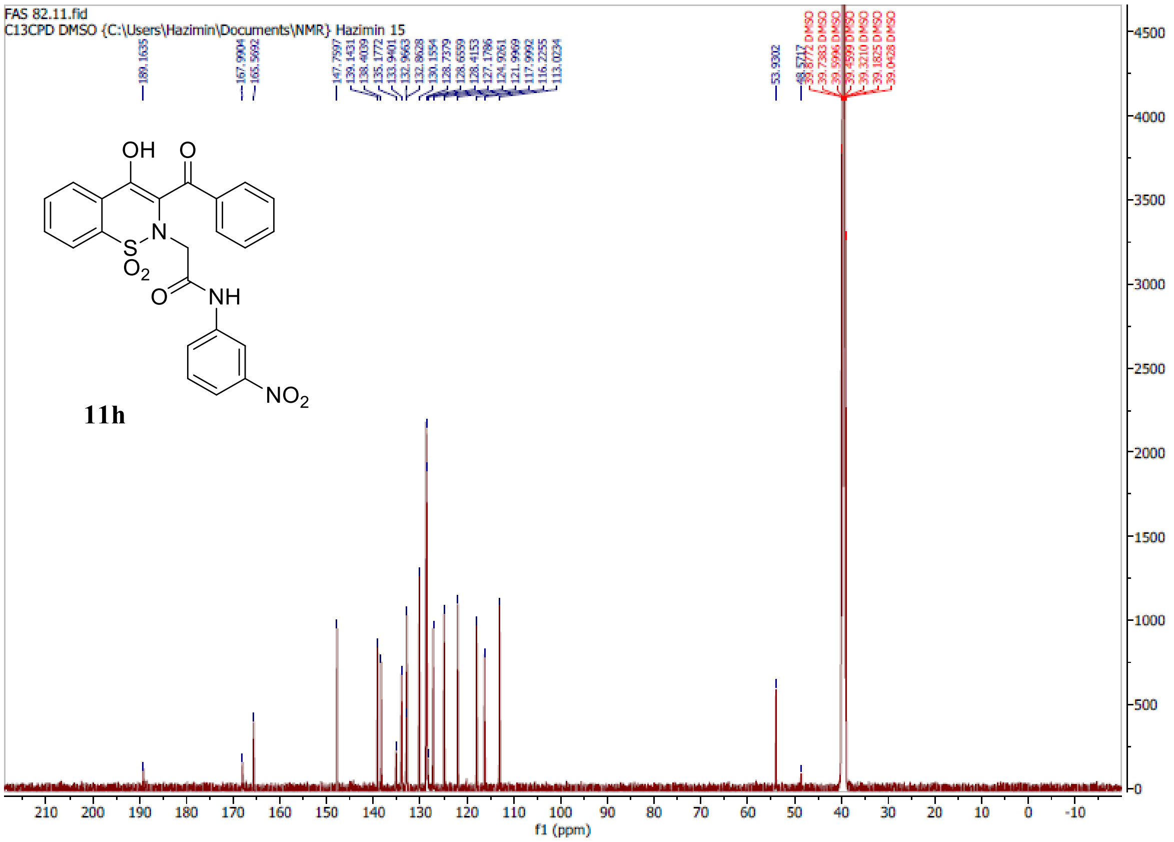

^{13}C NMR Spectrum of compound **11h.**

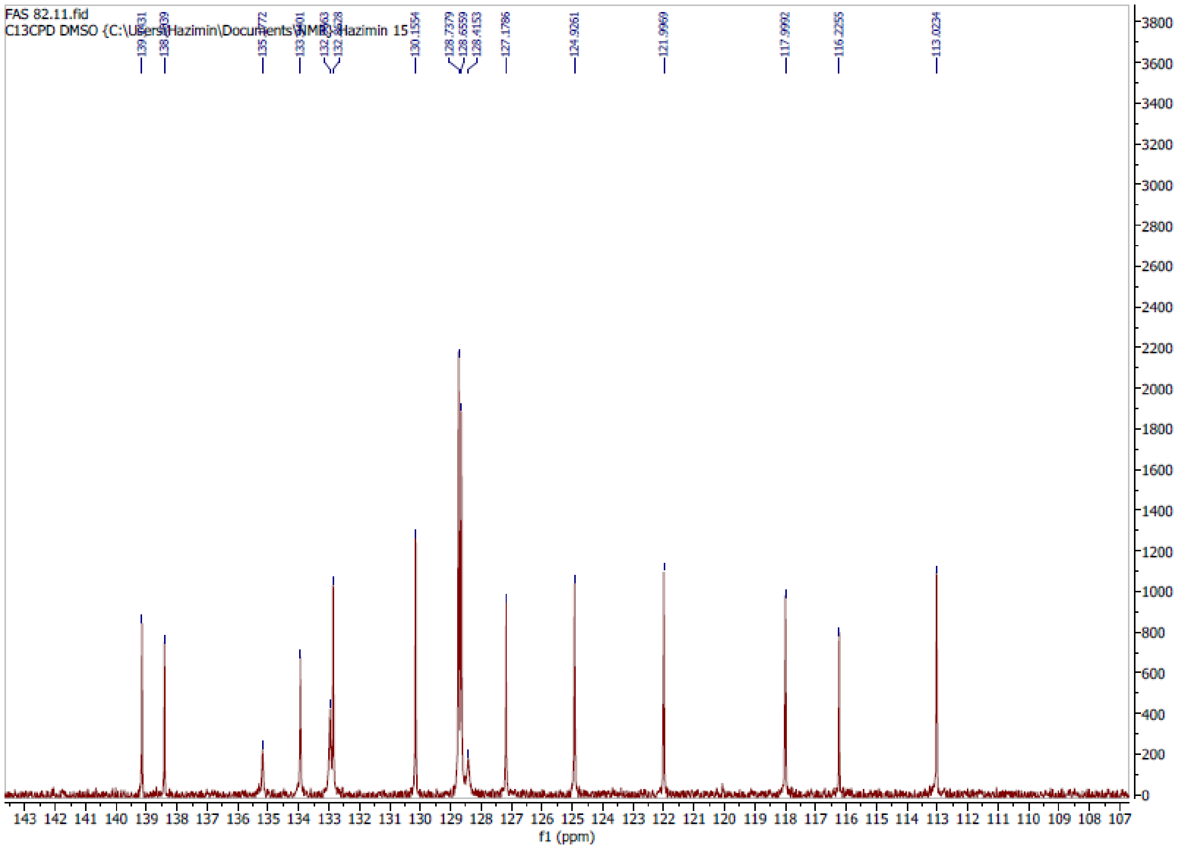

Expanded form of ^{13}C NMR Spectrum of compound **11h** (region 113-140 ppm).

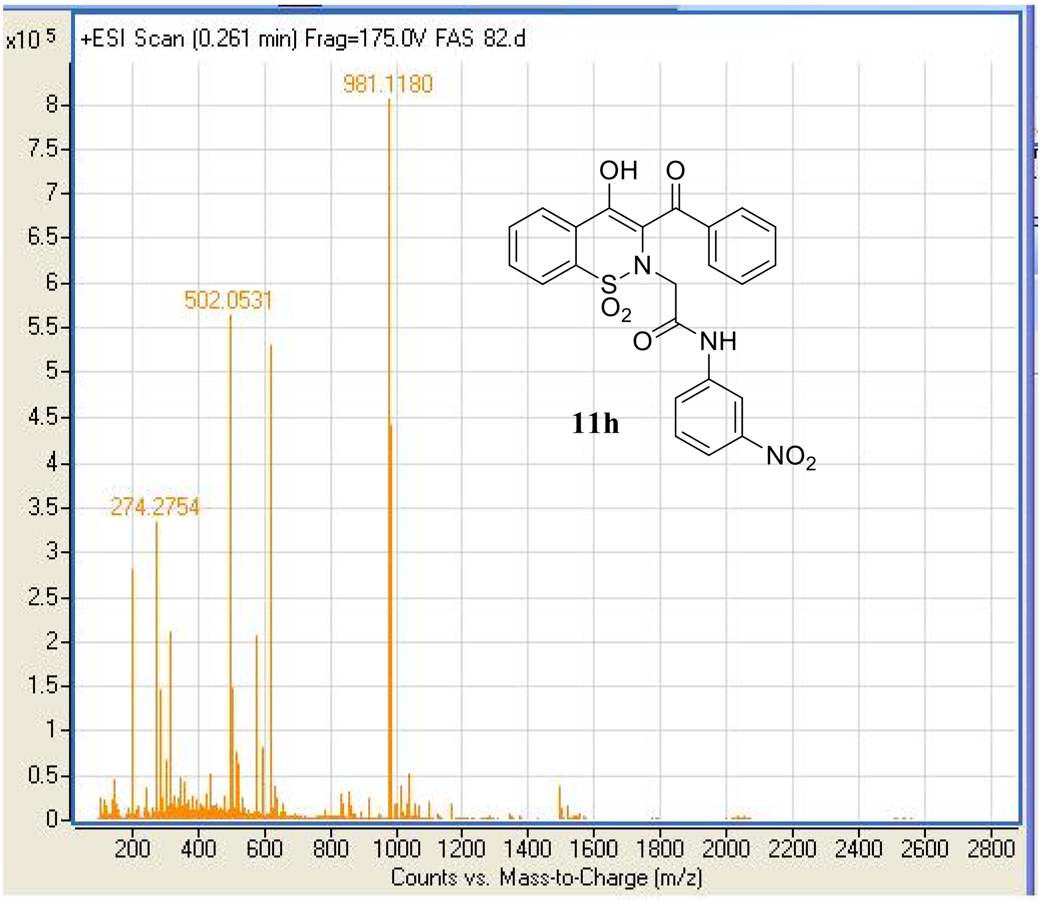

MS Spectrum of compound **11h.**

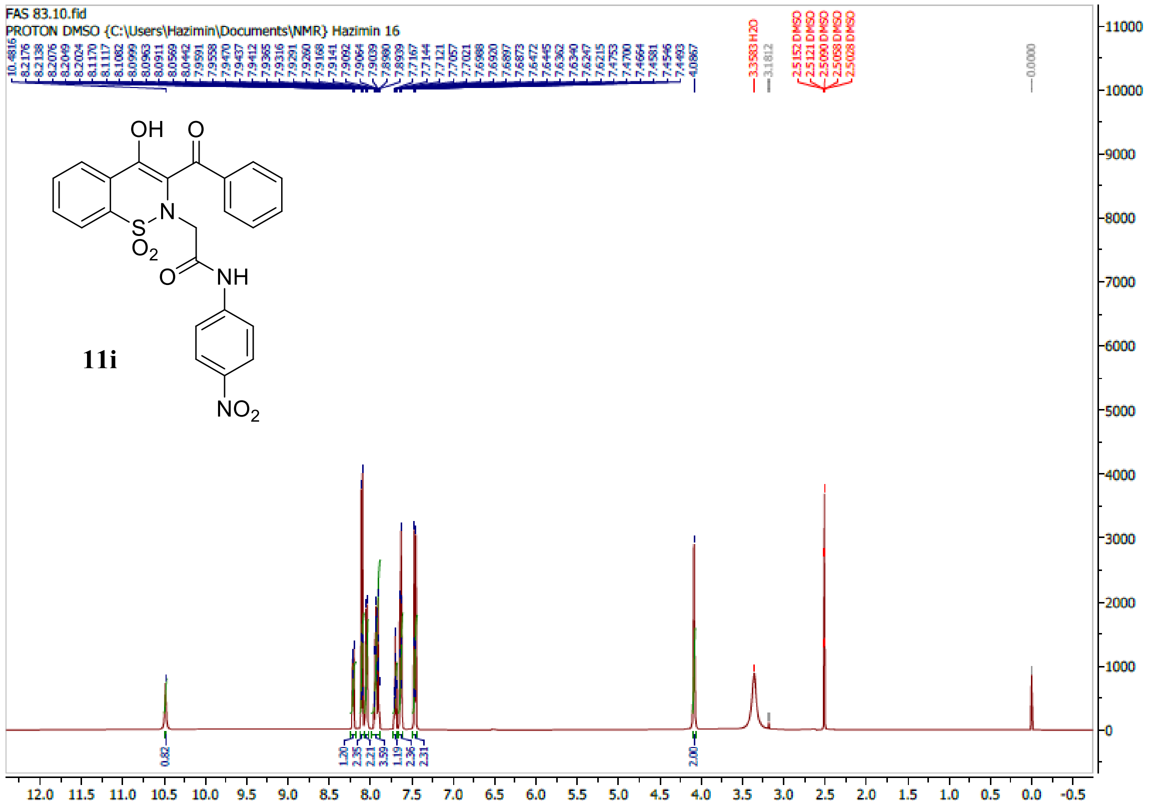

[1]*H* NMR Spectrum of compound **11i**.

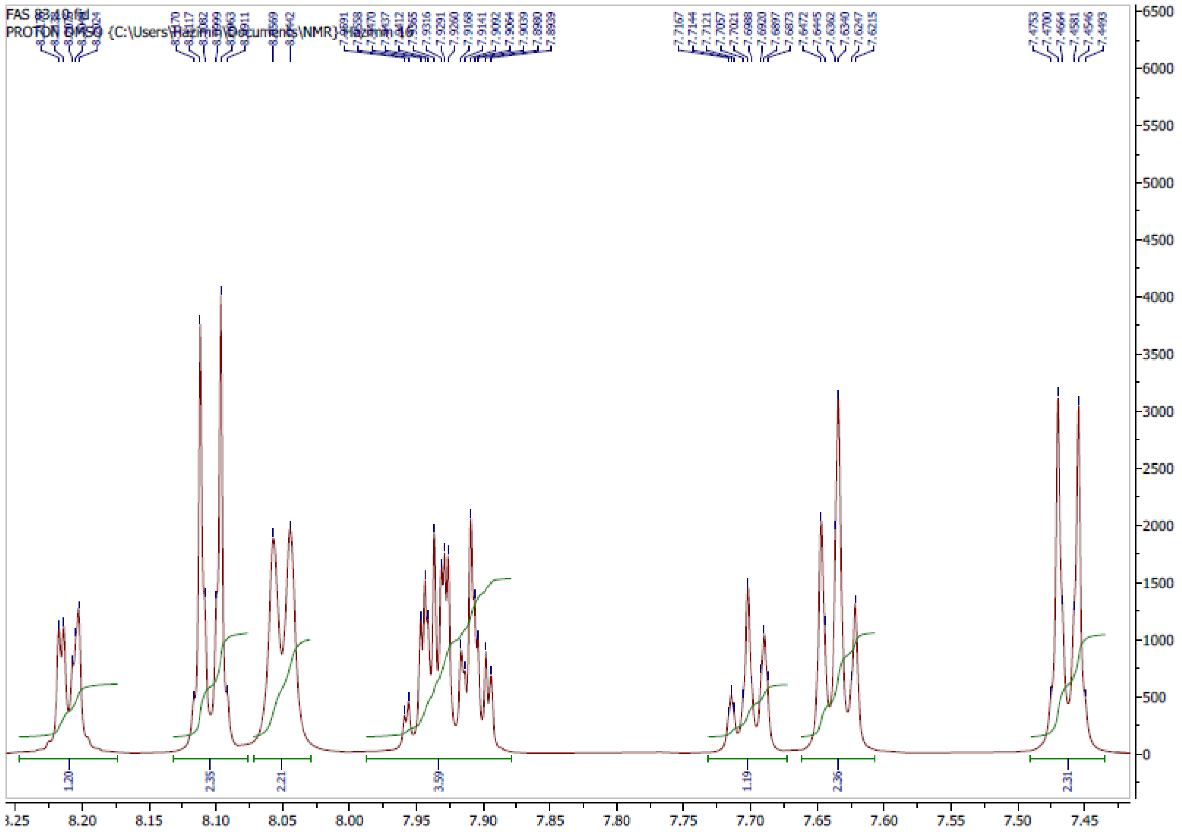

Expanded form of [1]*H* NMR Spectrum of compound **11i** (region 7.43-8.23 ppm).

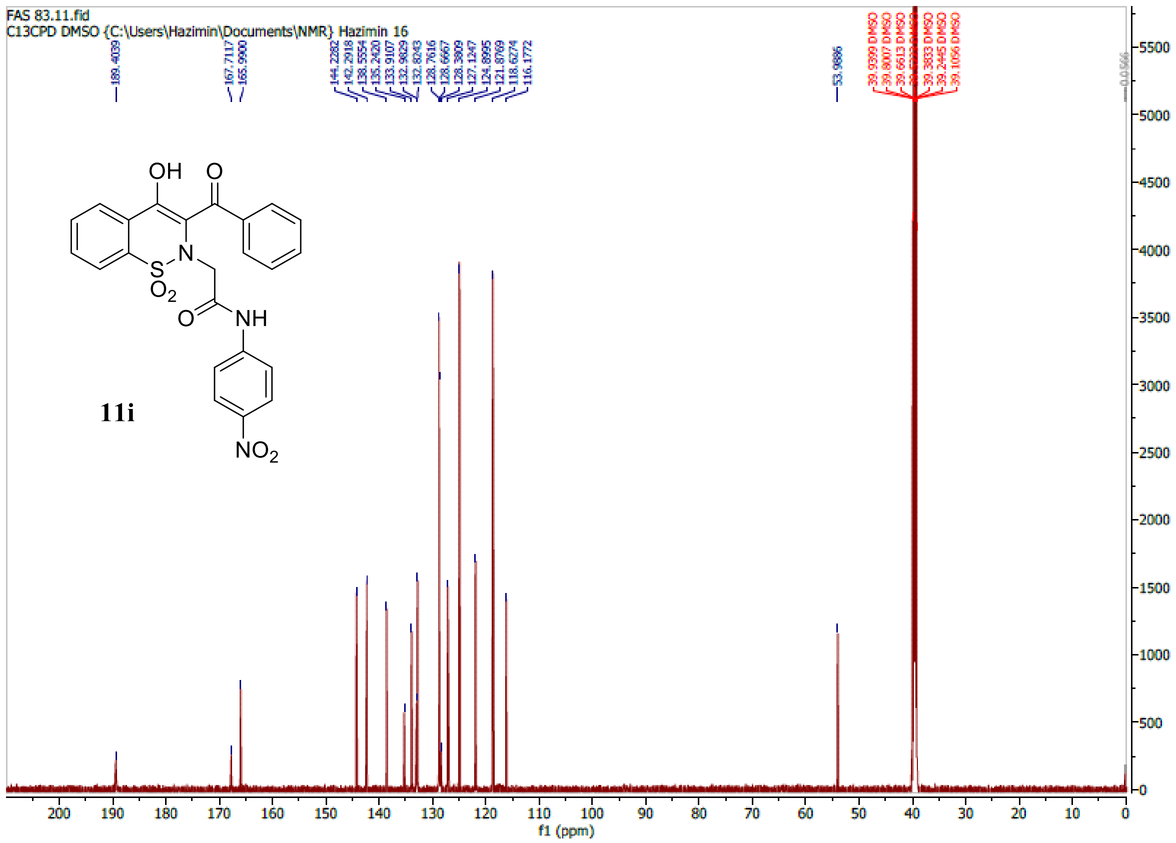

^{13}C NMR Spectrum of compound **11i.**

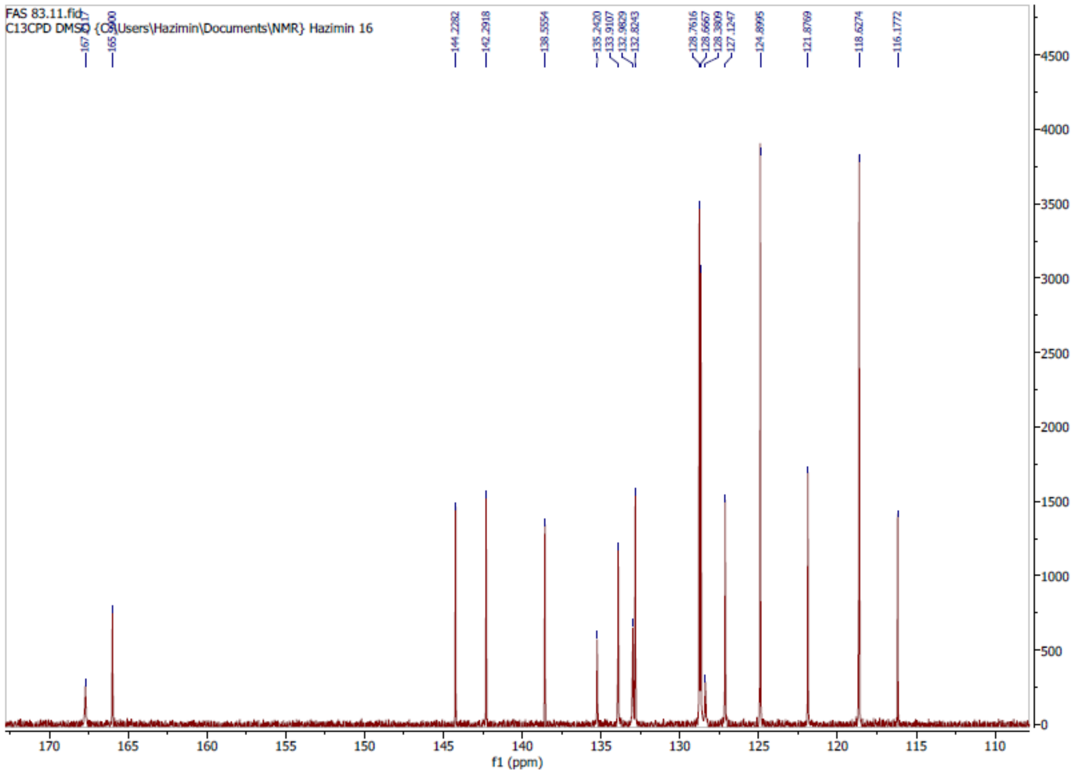

Expanded form of ^{13}C NMR Spectrum of compound **11i** (region 115-145 ppm).

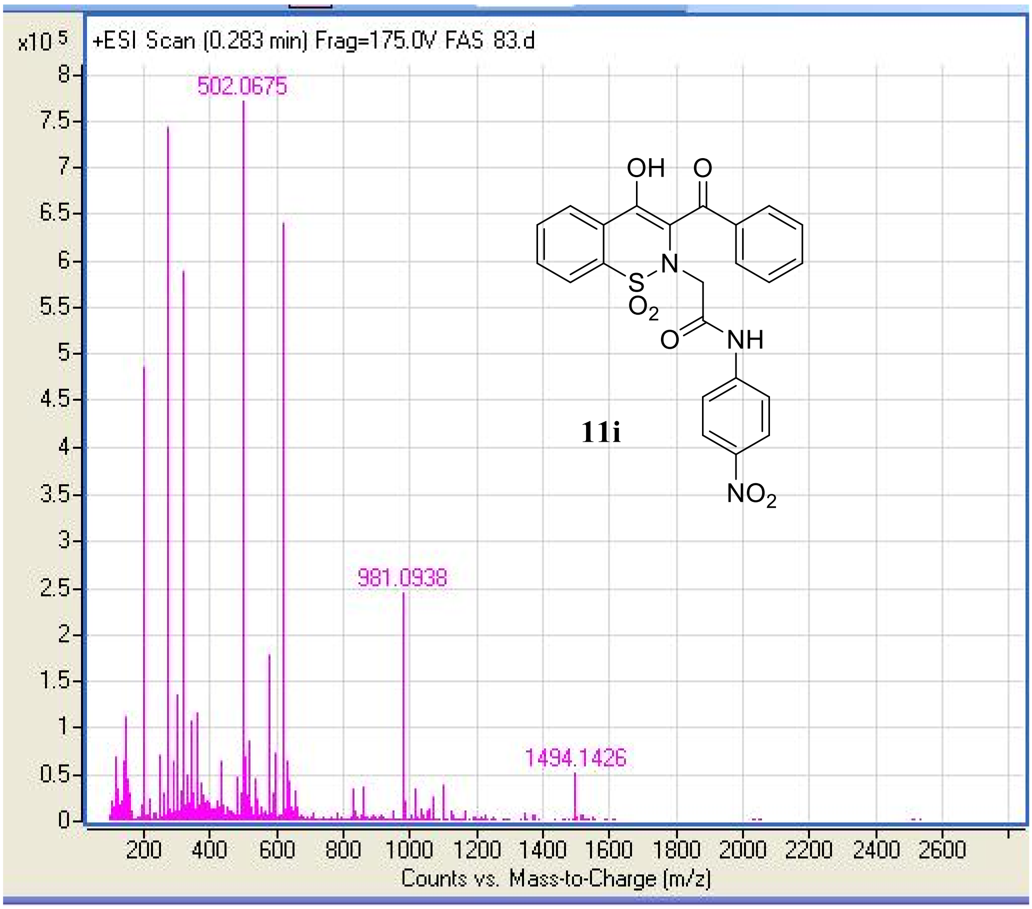

MS Spectrum of compound **11i.**

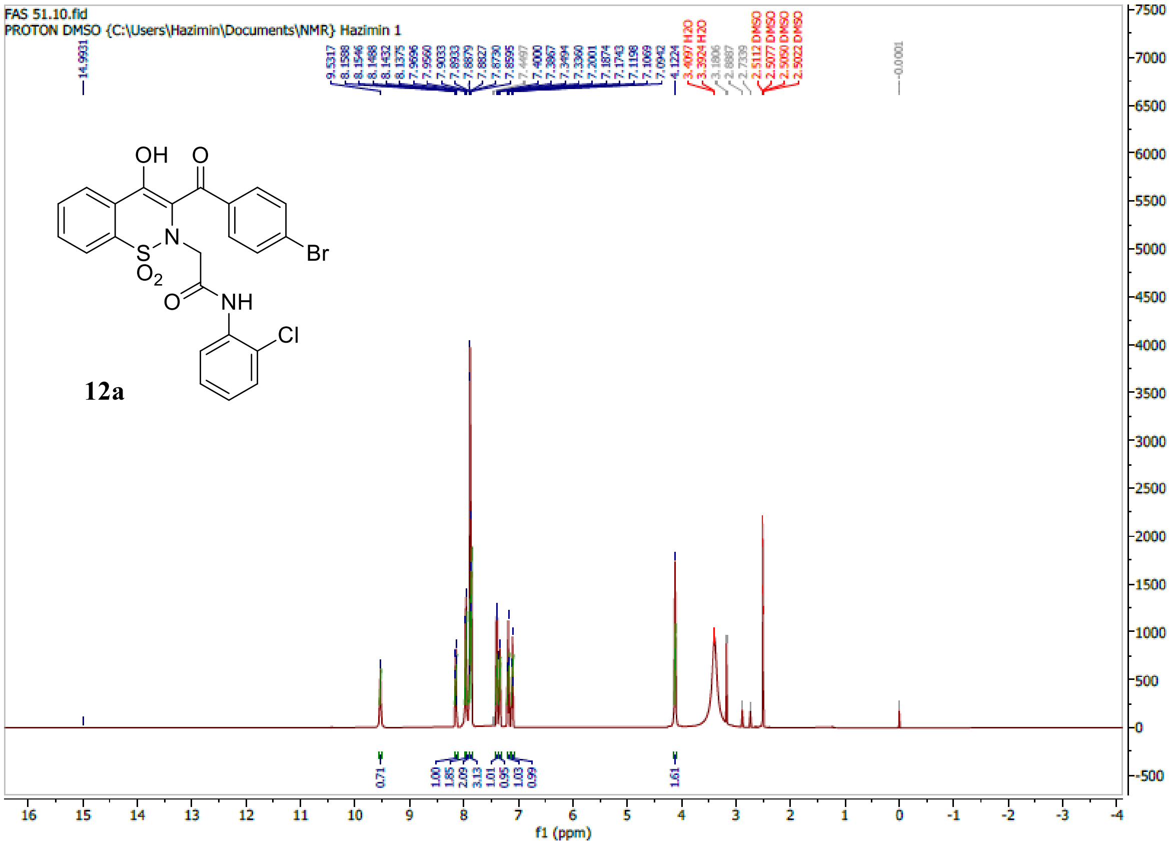

[1]*H* NMR Spectrum of compound **12a**.

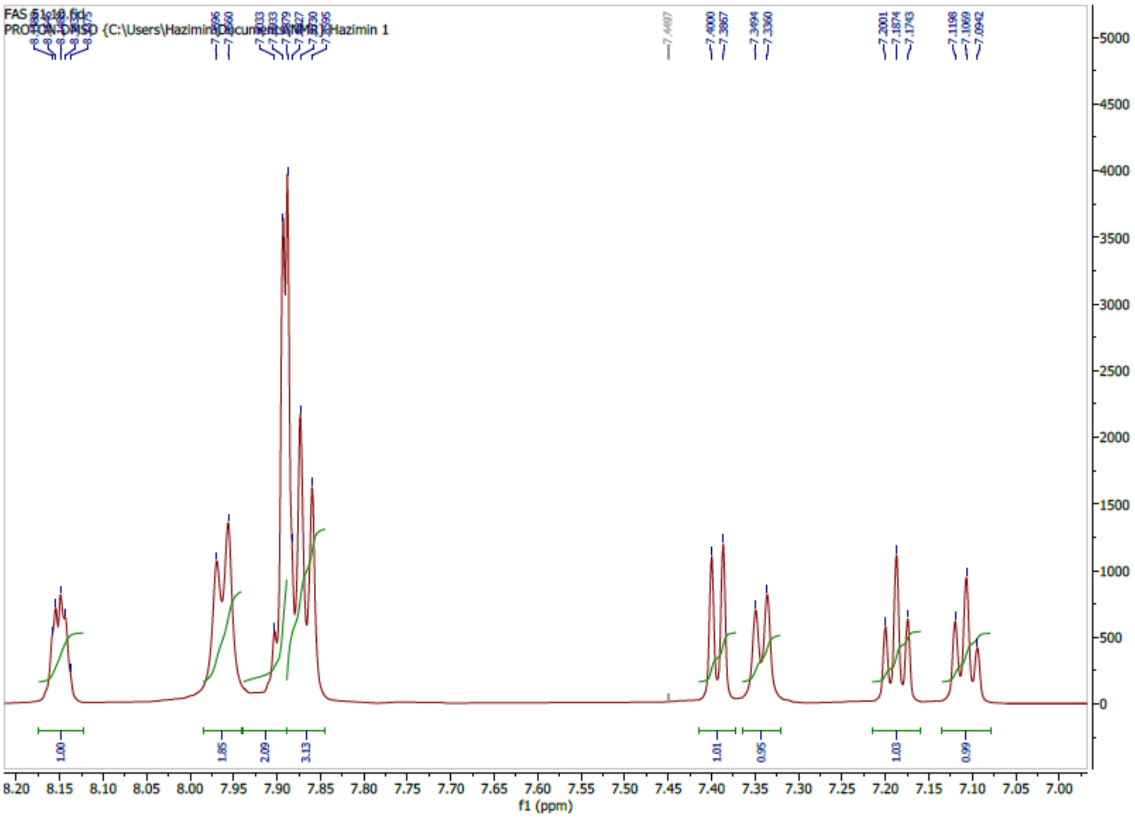

Expanded form of [1]*H* NMR Spectrum of compound **12a** (region 7.05-8.20 ppm).

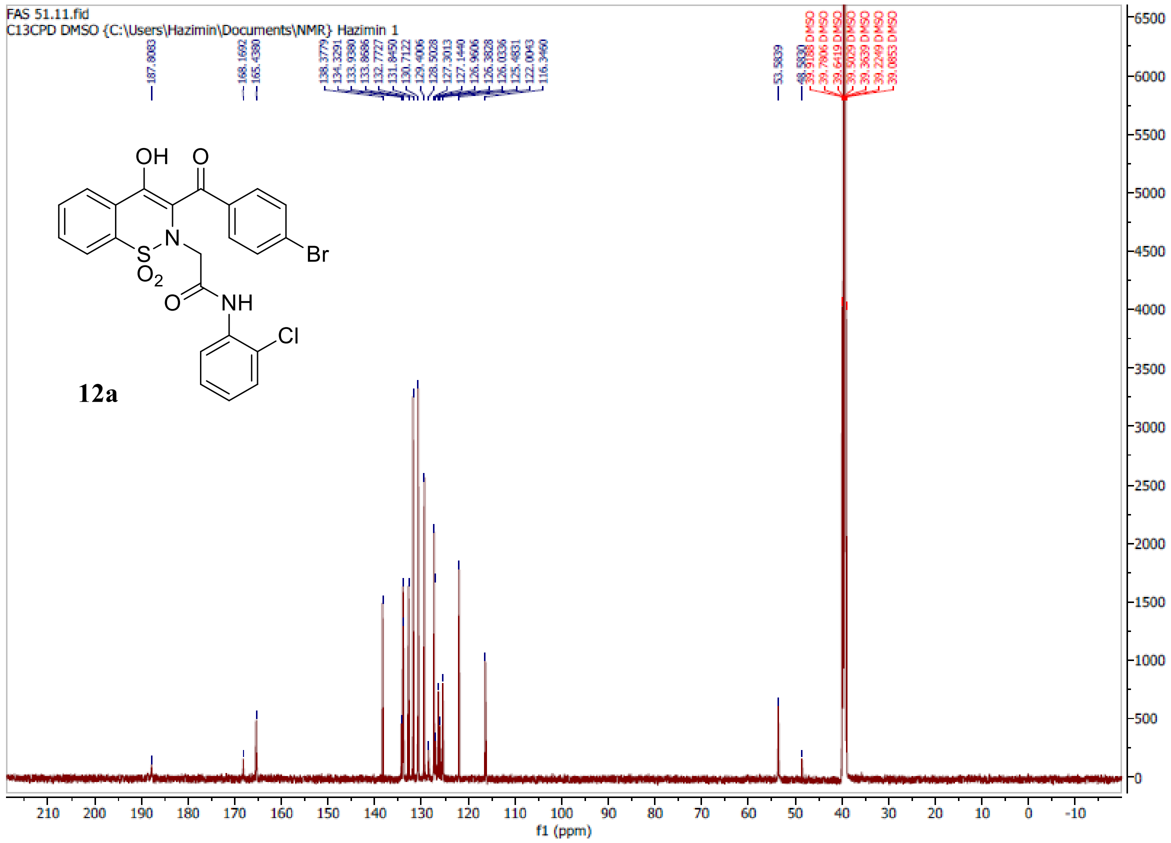

^{13}C NMR Spectrum of compound **12a.**

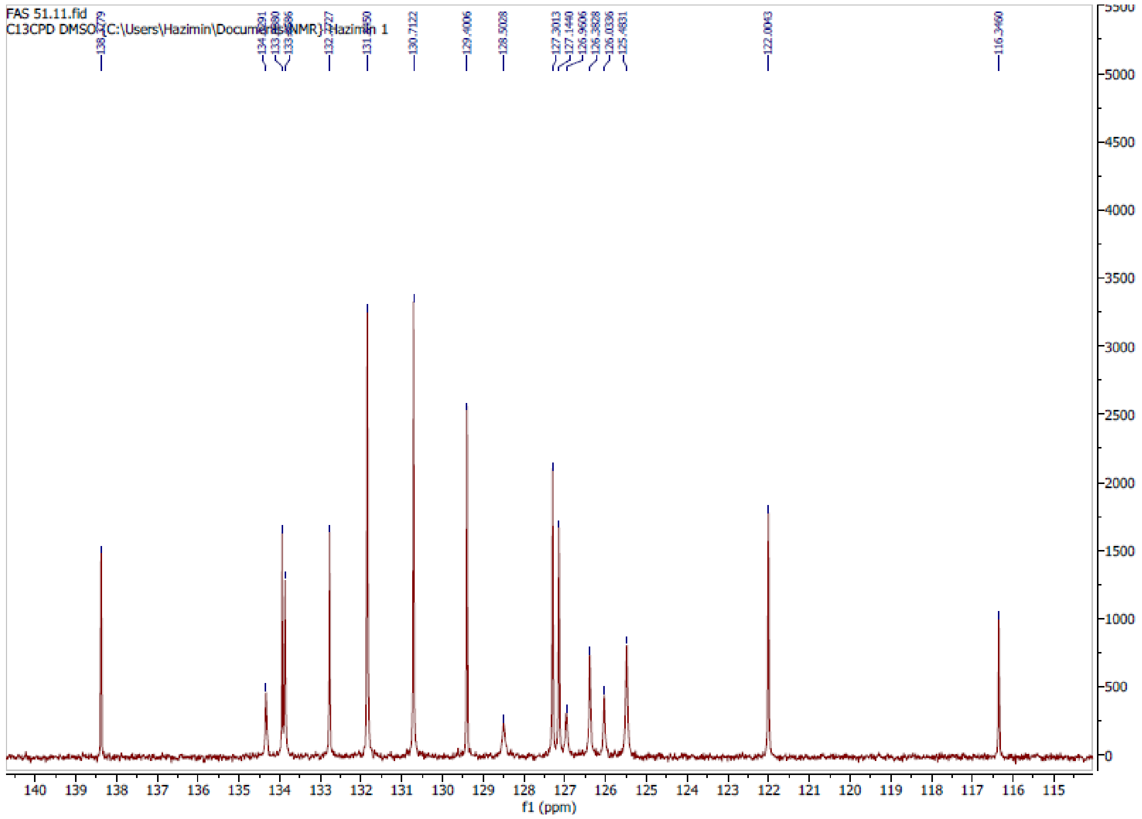

Expanded form of ^{13}C NMR Spectrum of compound **12a** (region 116-139 ppm).

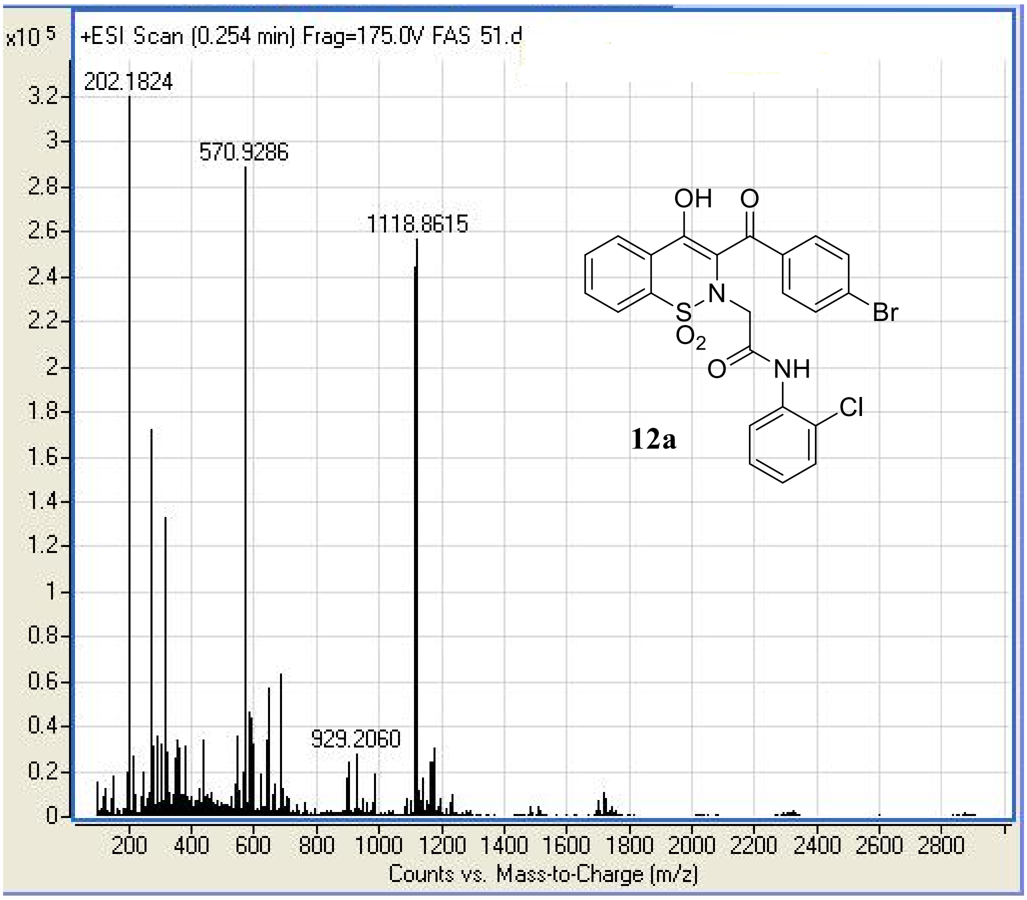

MS Spectrum of compound **12a.**

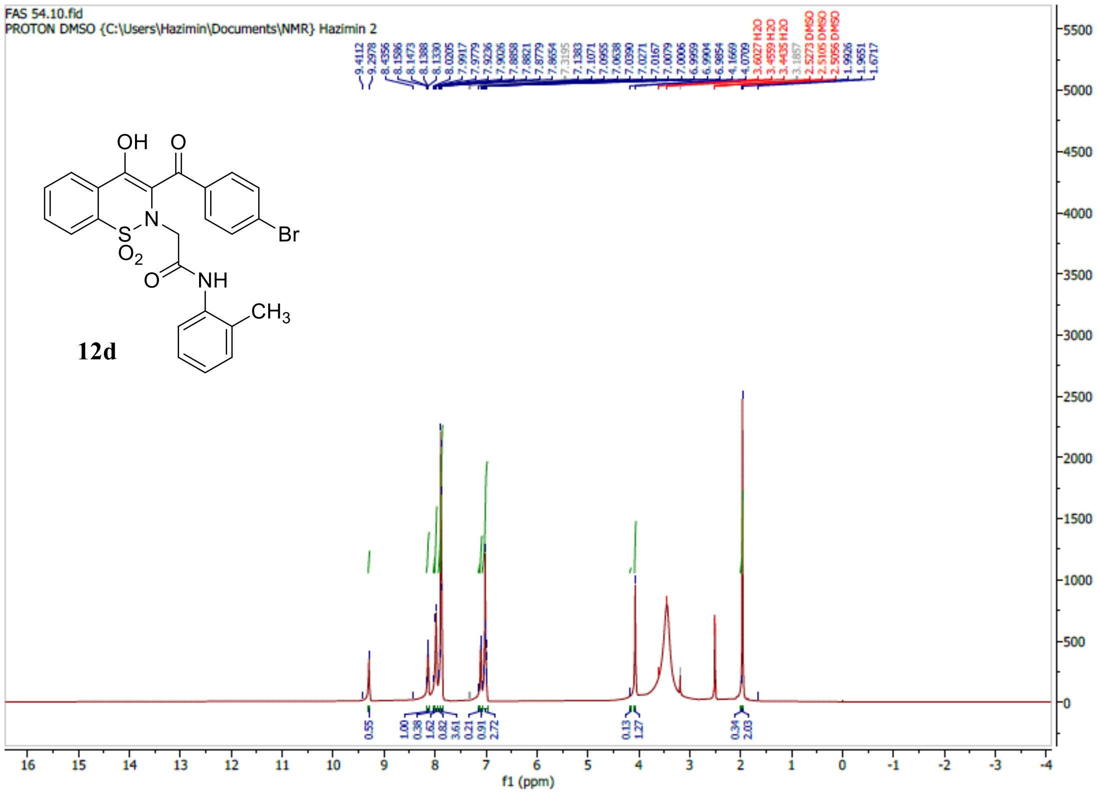

[1]*H* NMR Spectrum of compound **12d**.

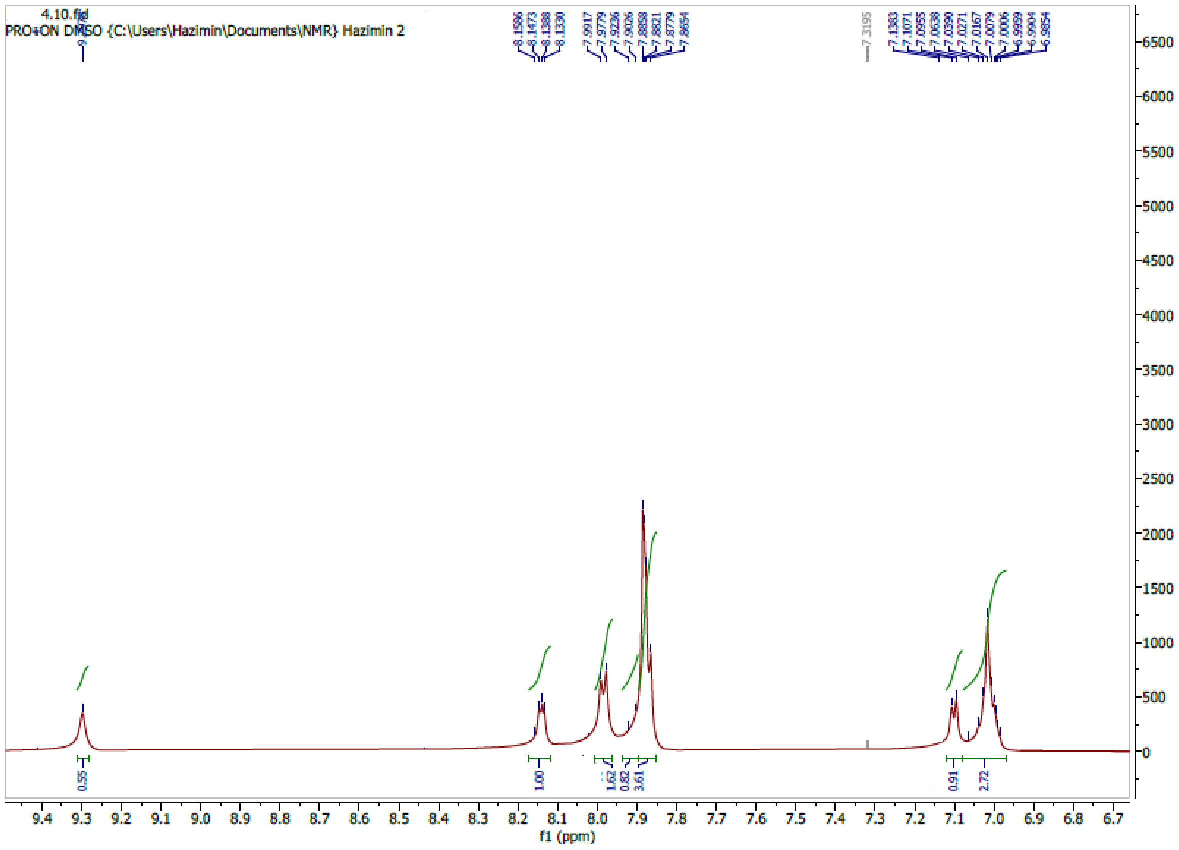

Expanded form of ^{1}H NMR Spectrum of compound **12d** (region 6.90-8.20 ppm).

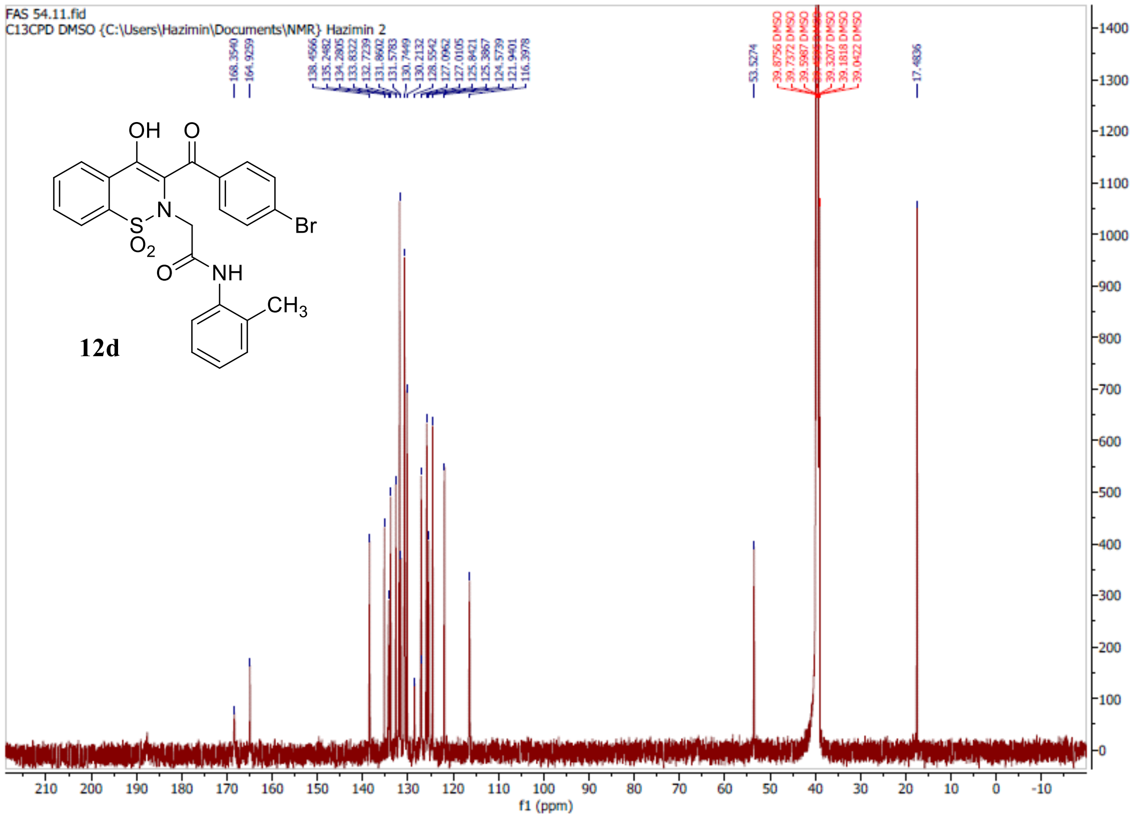

^{13}C NMR Spectrum of compound **12d.**

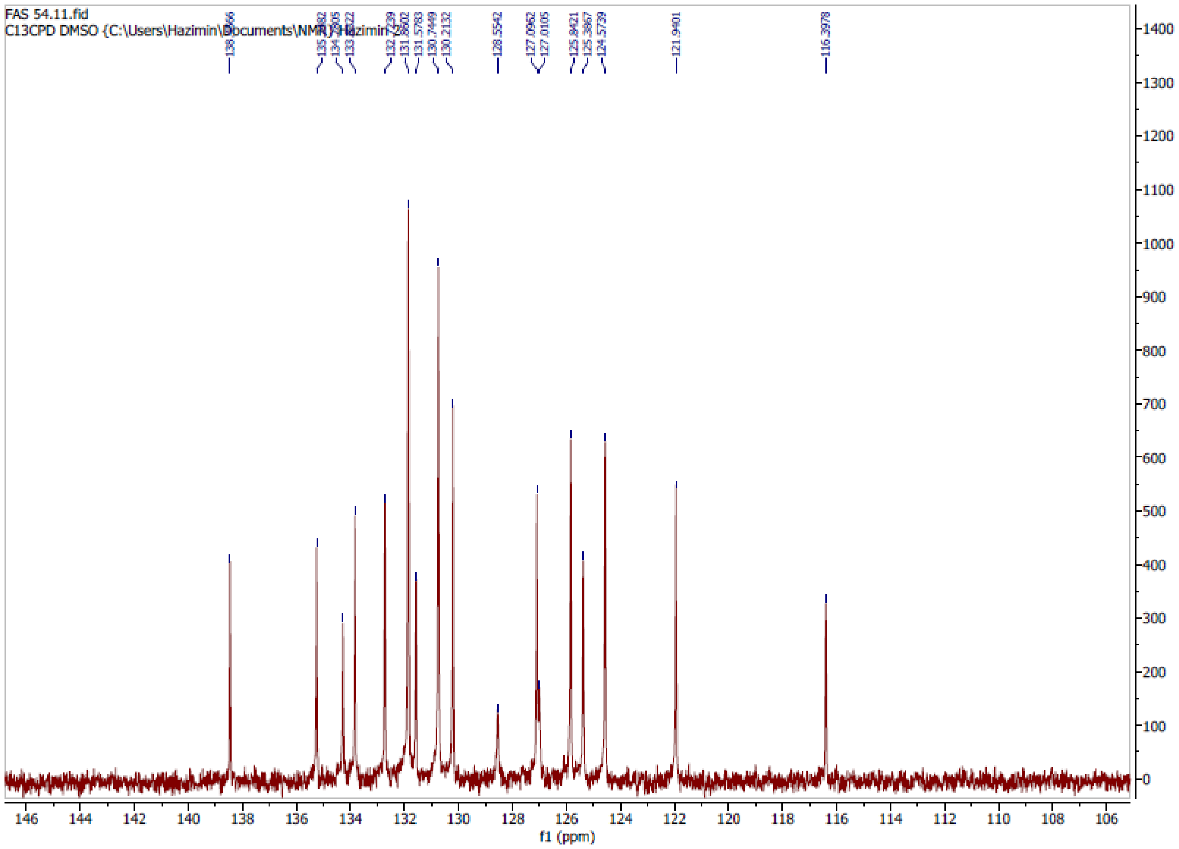

Expanded form of ^{13}C NMR Spectrum of compound **12d** (region 116-140 ppm).

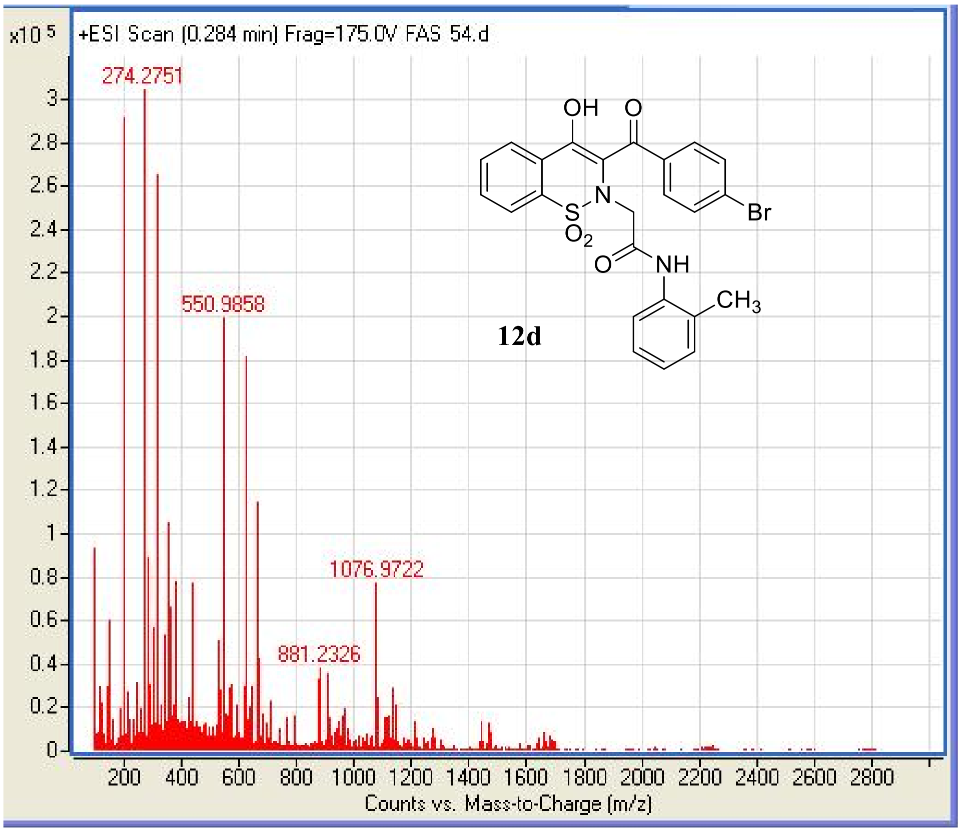

MS Spectrum of compound **12d.**

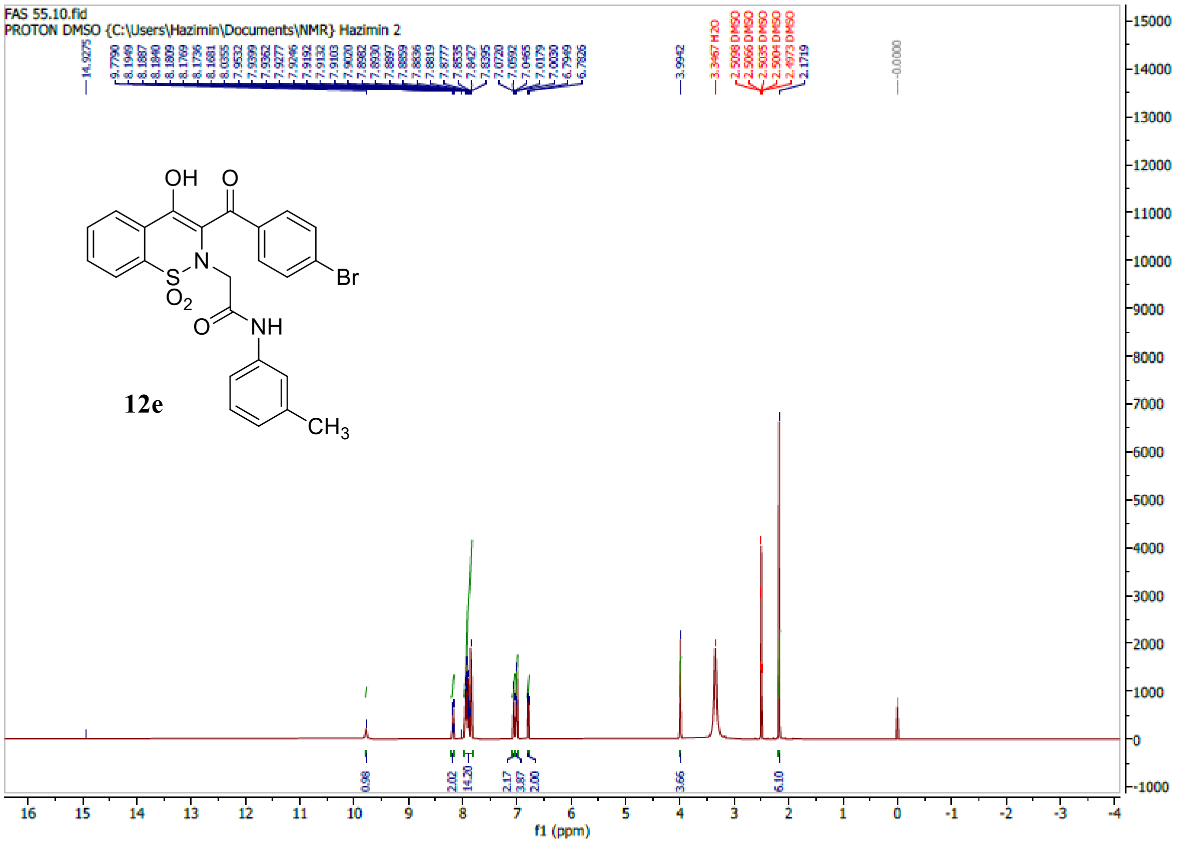

[1]*H* NMR Spectrum of compound **12e**.

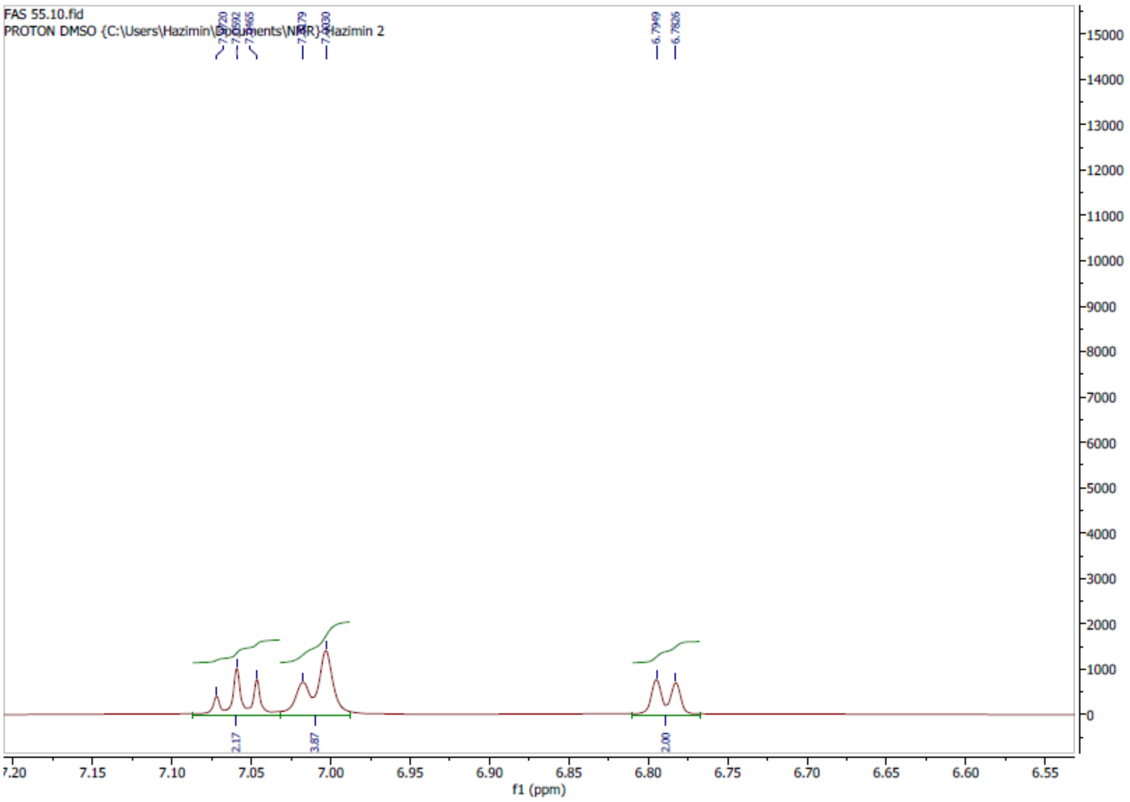

Expanded form of [1]*H* NMR Spectrum of compound **12e** (region 6.75-7.10 ppm).

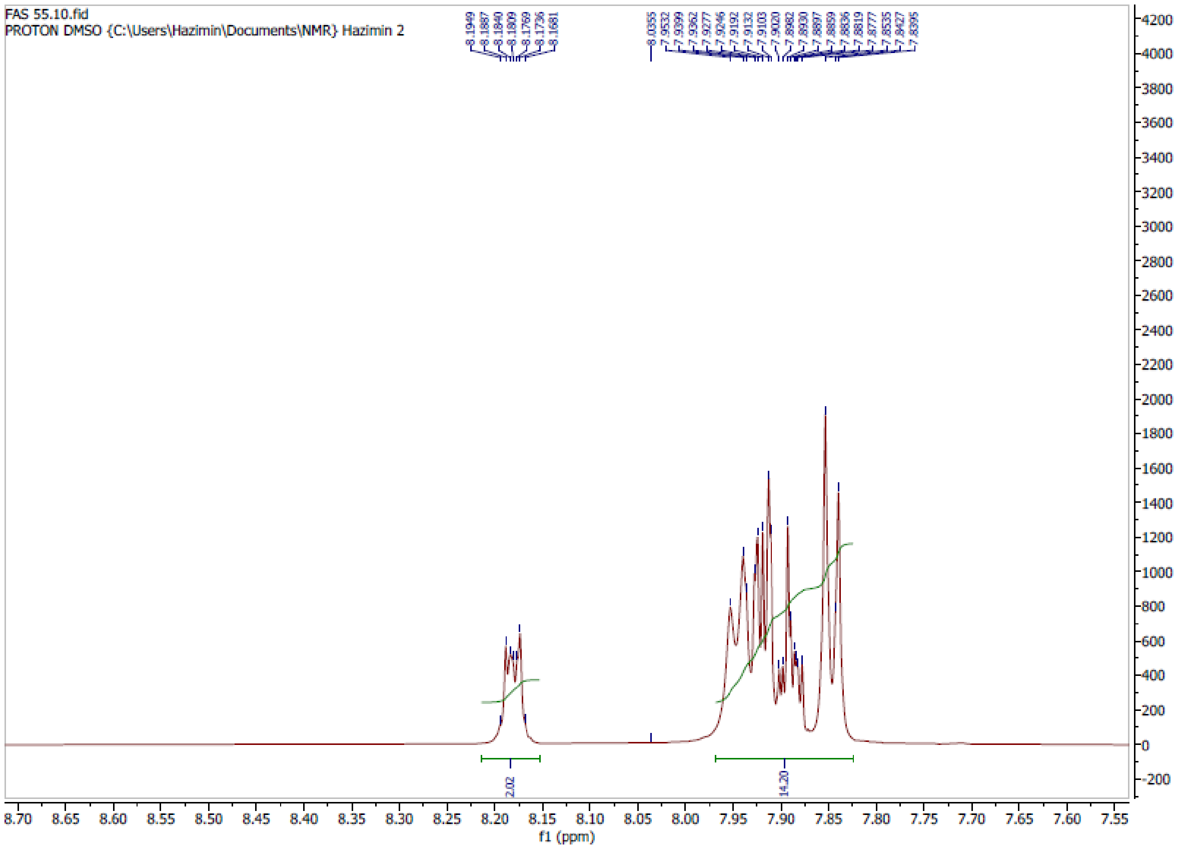

Expanded form of ¹*H* NMR Spectrum of compound **12e** (region 7.80-8.25 ppm).

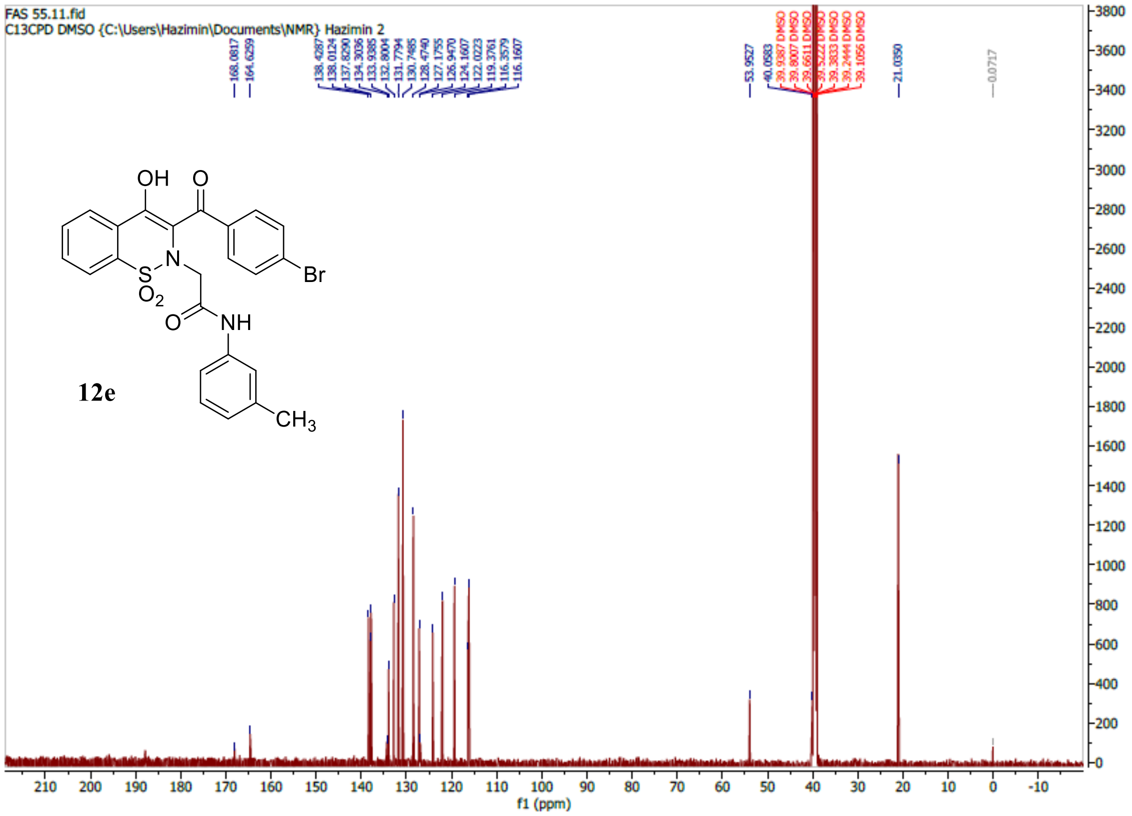

^{13}*C* NMR Spectrum of compound **12e.**

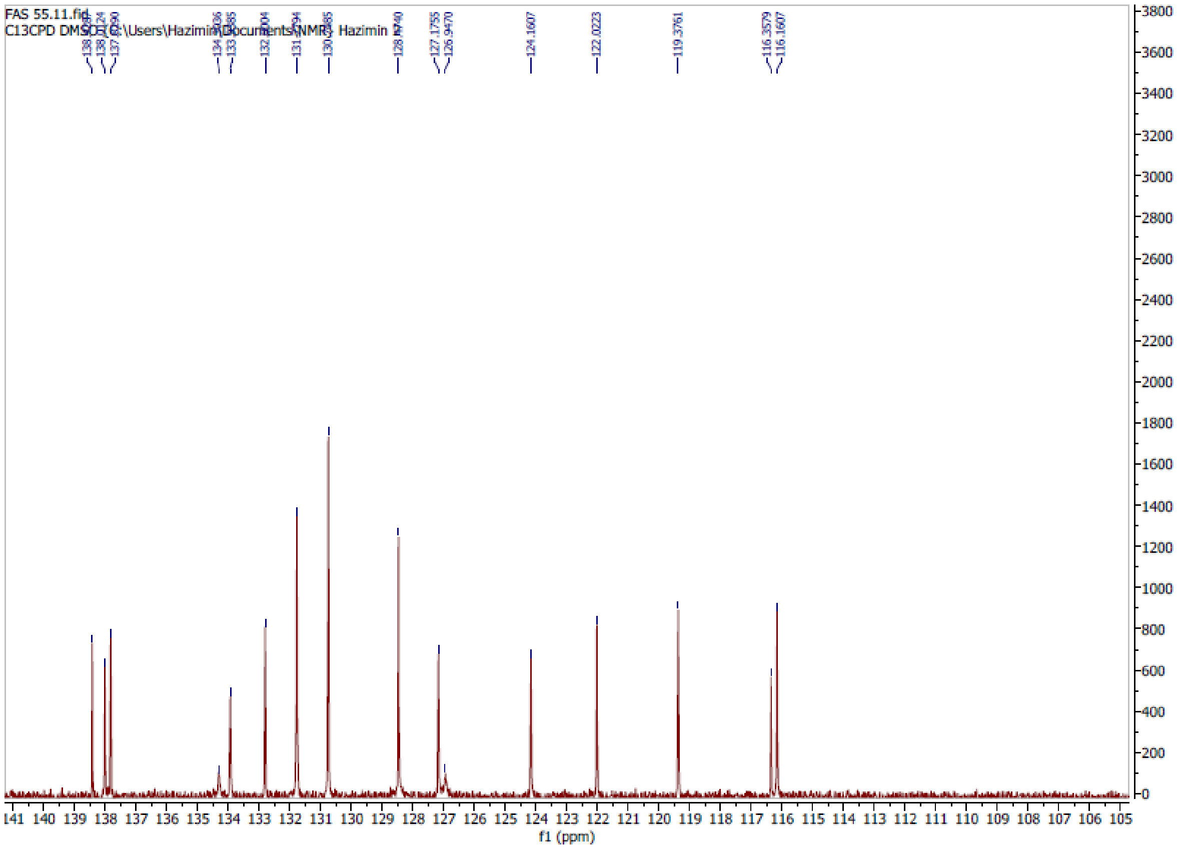

Expanded form of ^{13}C NMR Spectrum of compound **12e** (region 116-139 ppm).

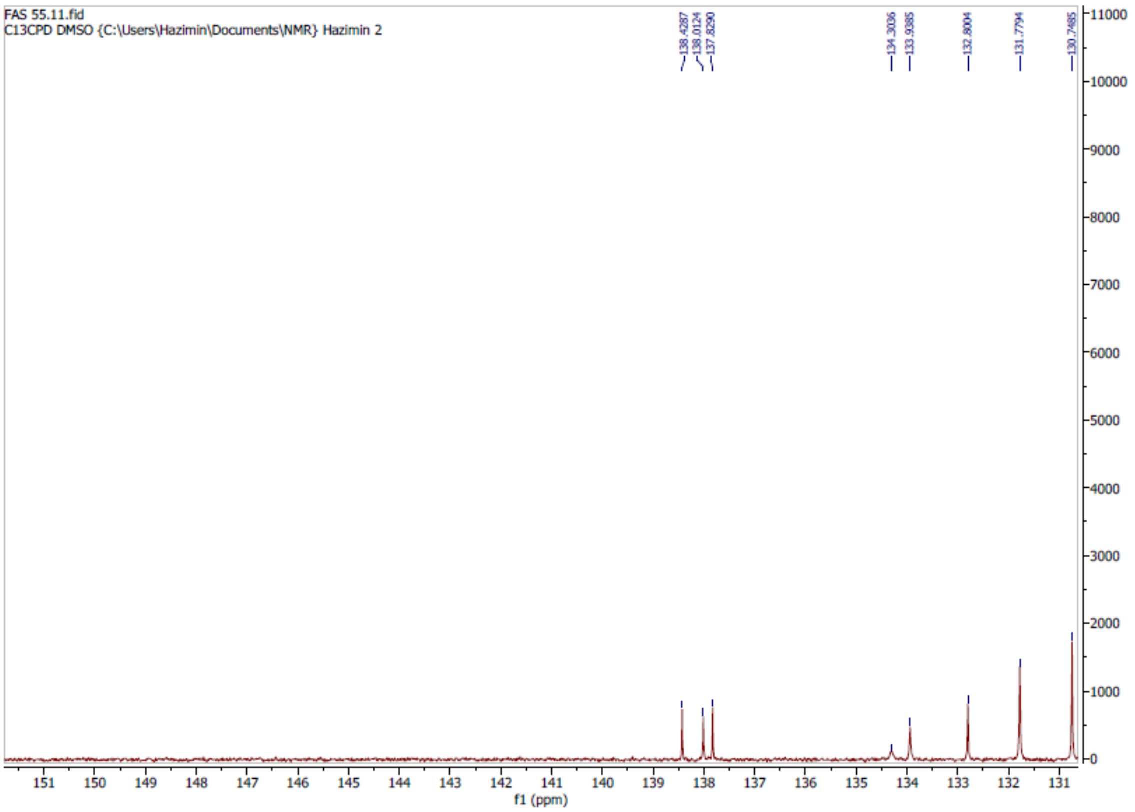

Expanded form of ^{13}C NMR Spectrum of compound **12e** (region 130-139 ppm).

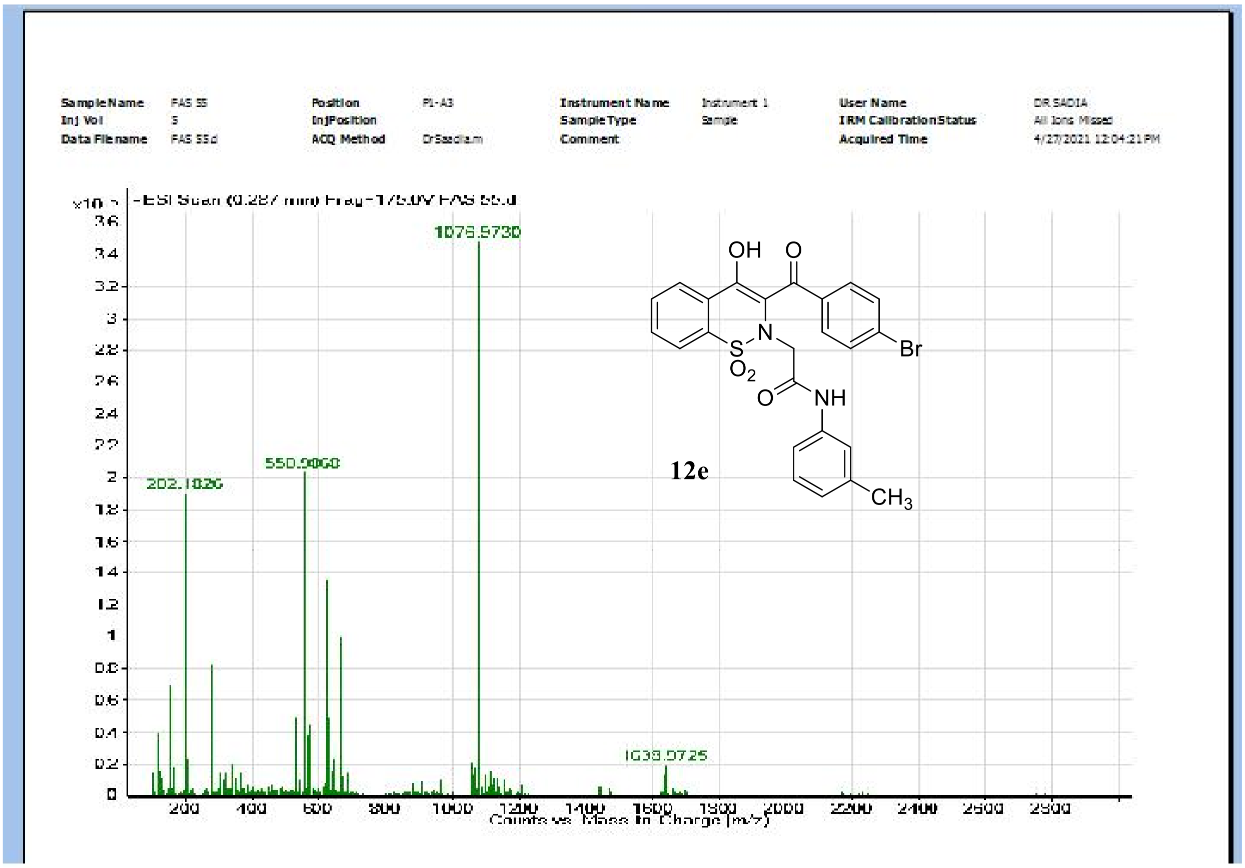

MS Spectrum of compound **12e.**

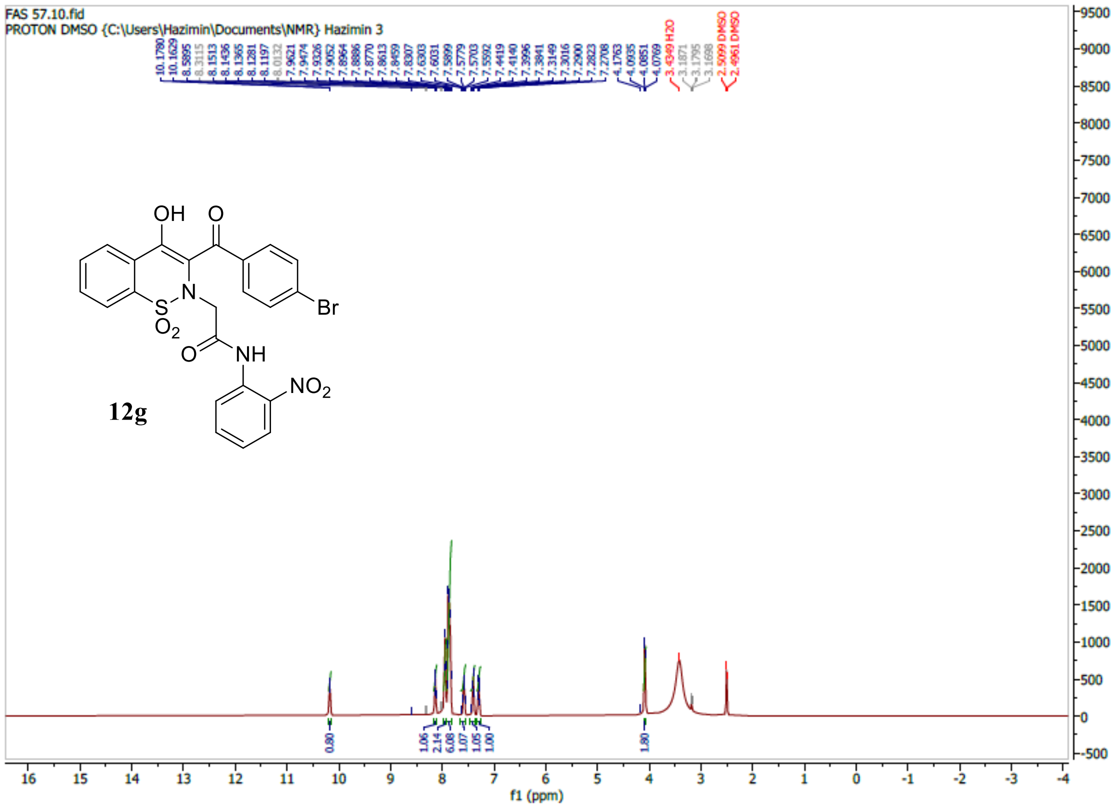

^{1}H NMR Spectrum of compound **12g**.

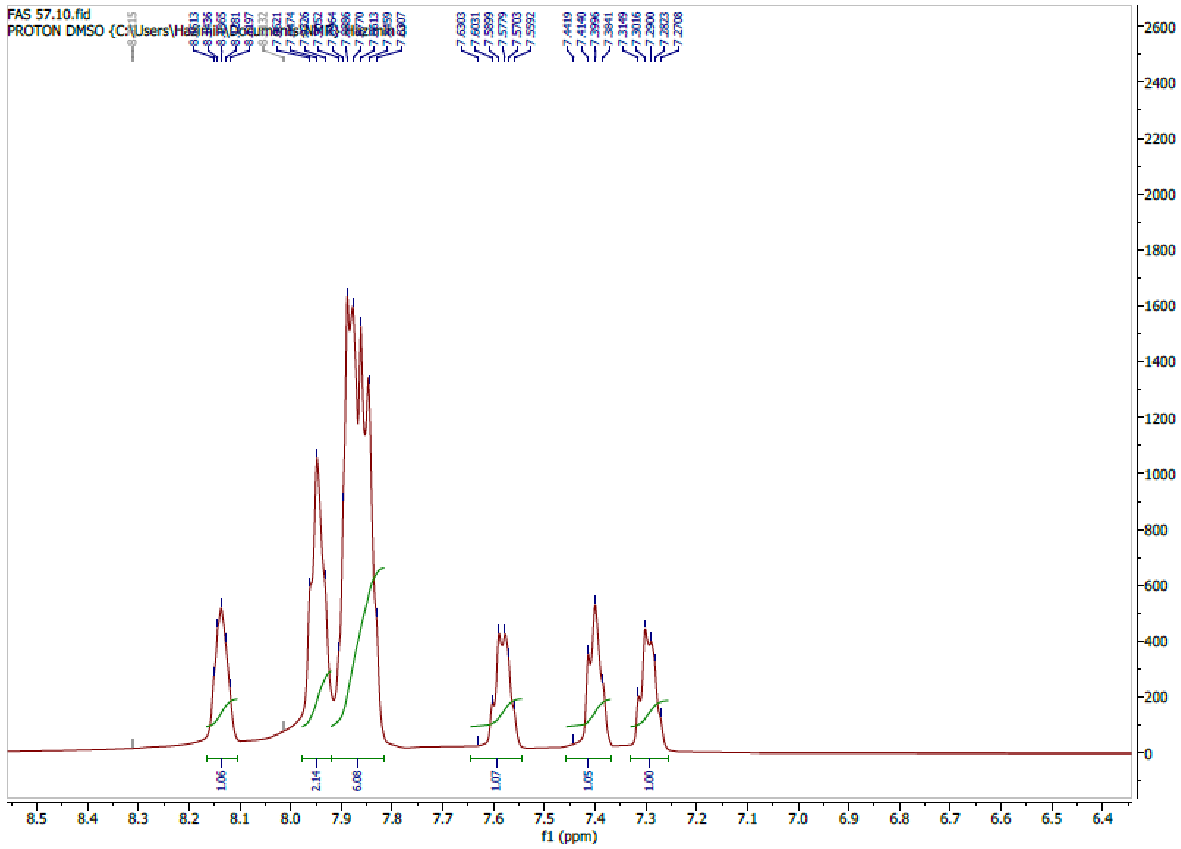

Expanded form of ^{1}H NMR Spectrum of compound **12g** (region 7.20-8.20 ppm).

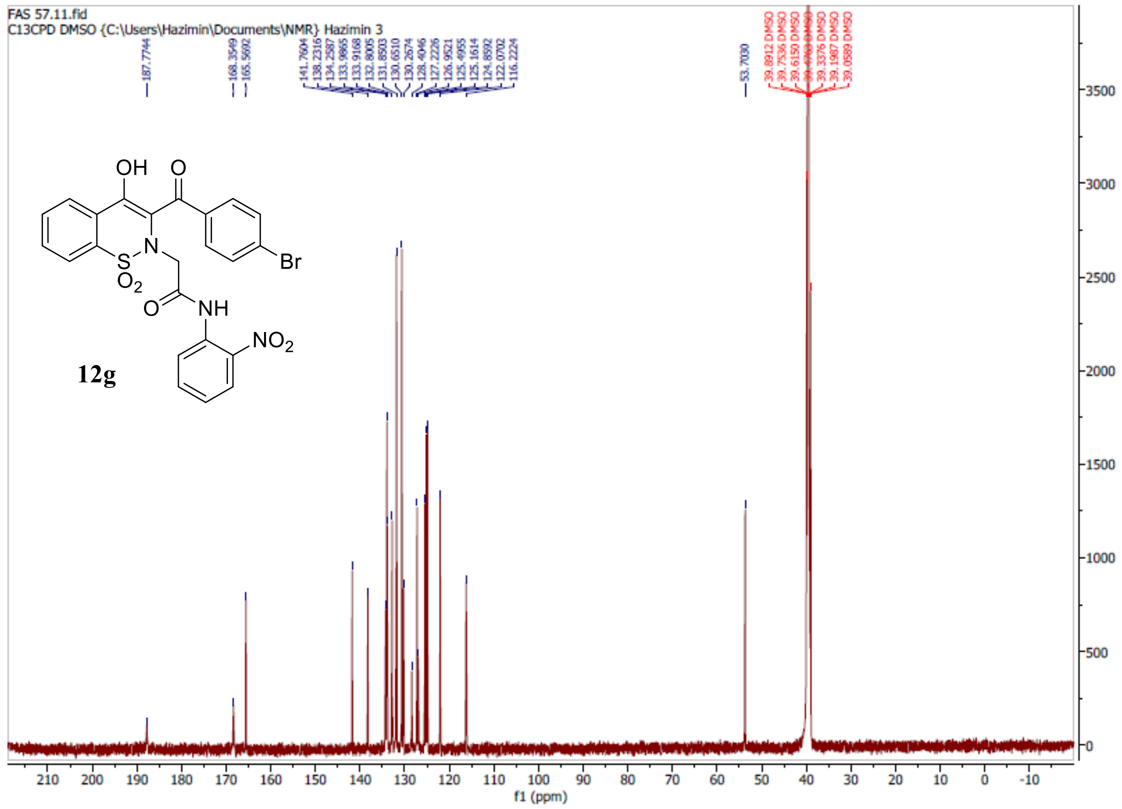

^{13}C NMR Spectrum of compound **12g.**

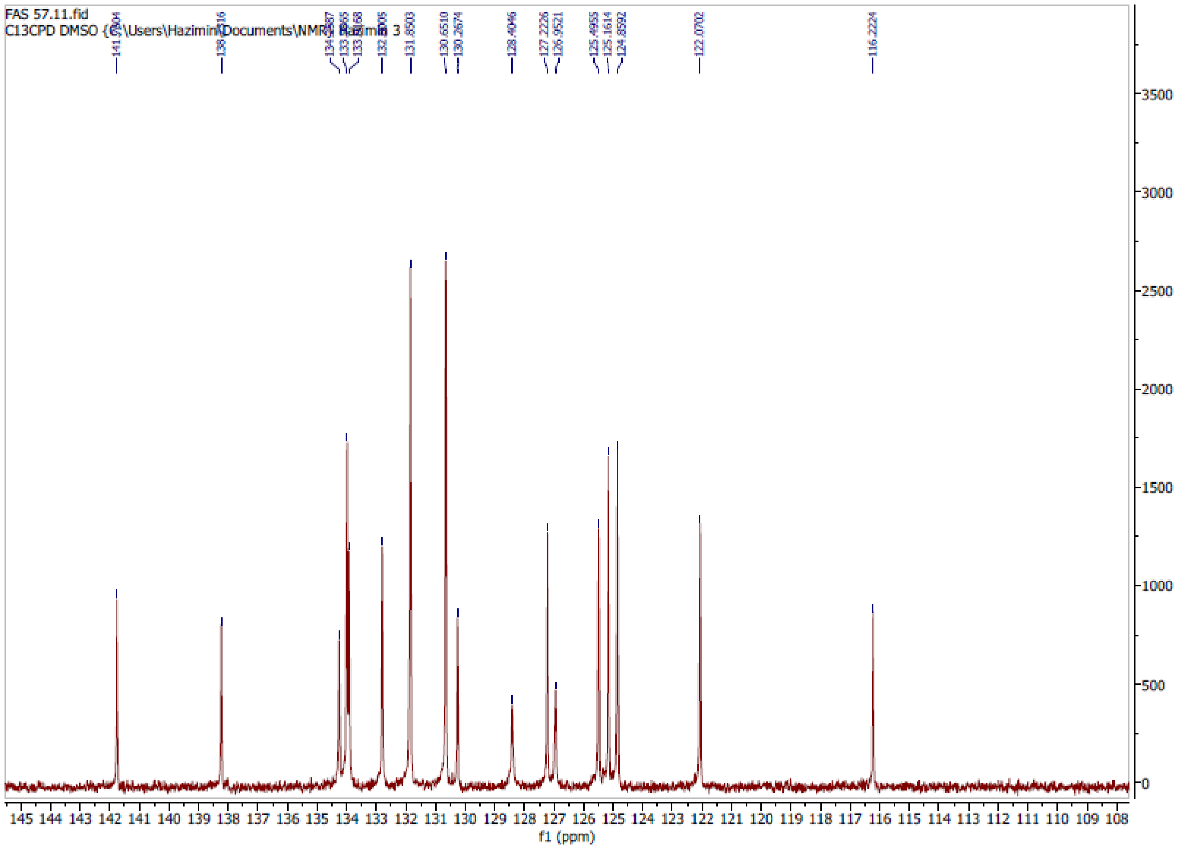

Expanded form of ^{13}C NMR Spectrum of compound **12g** (region 116-142 ppm).

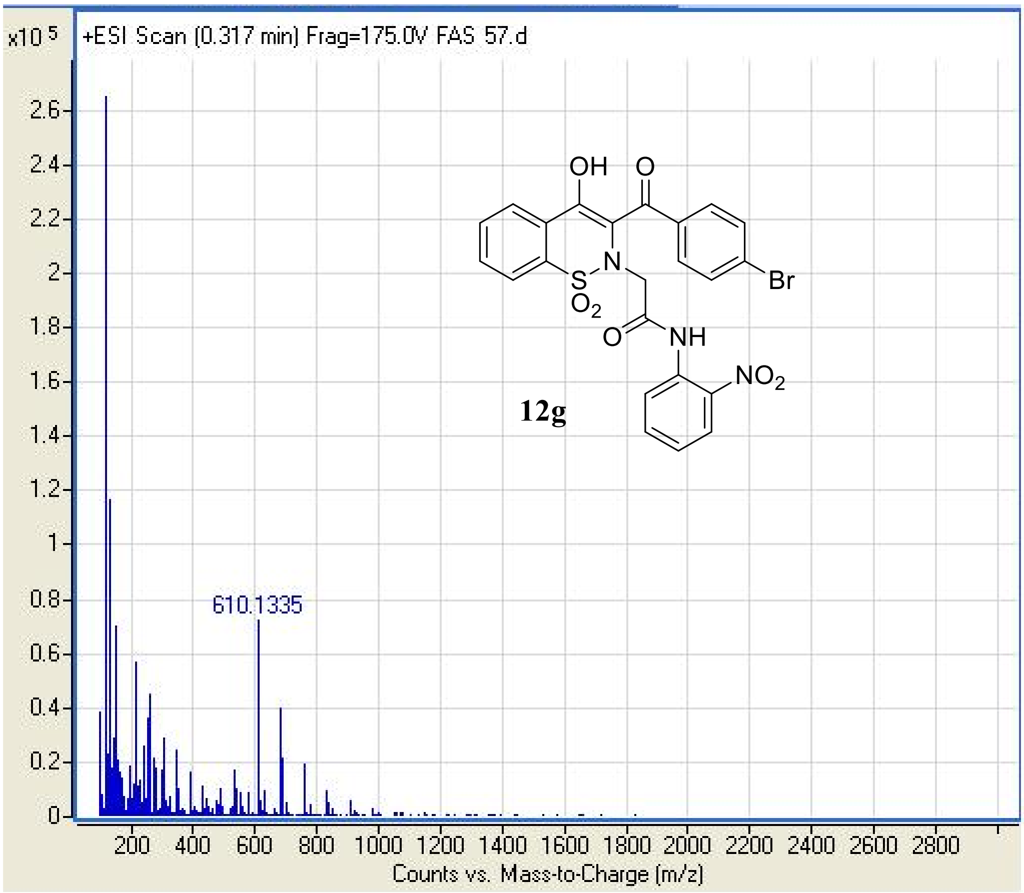

MS Spectrum of compound **12g.**

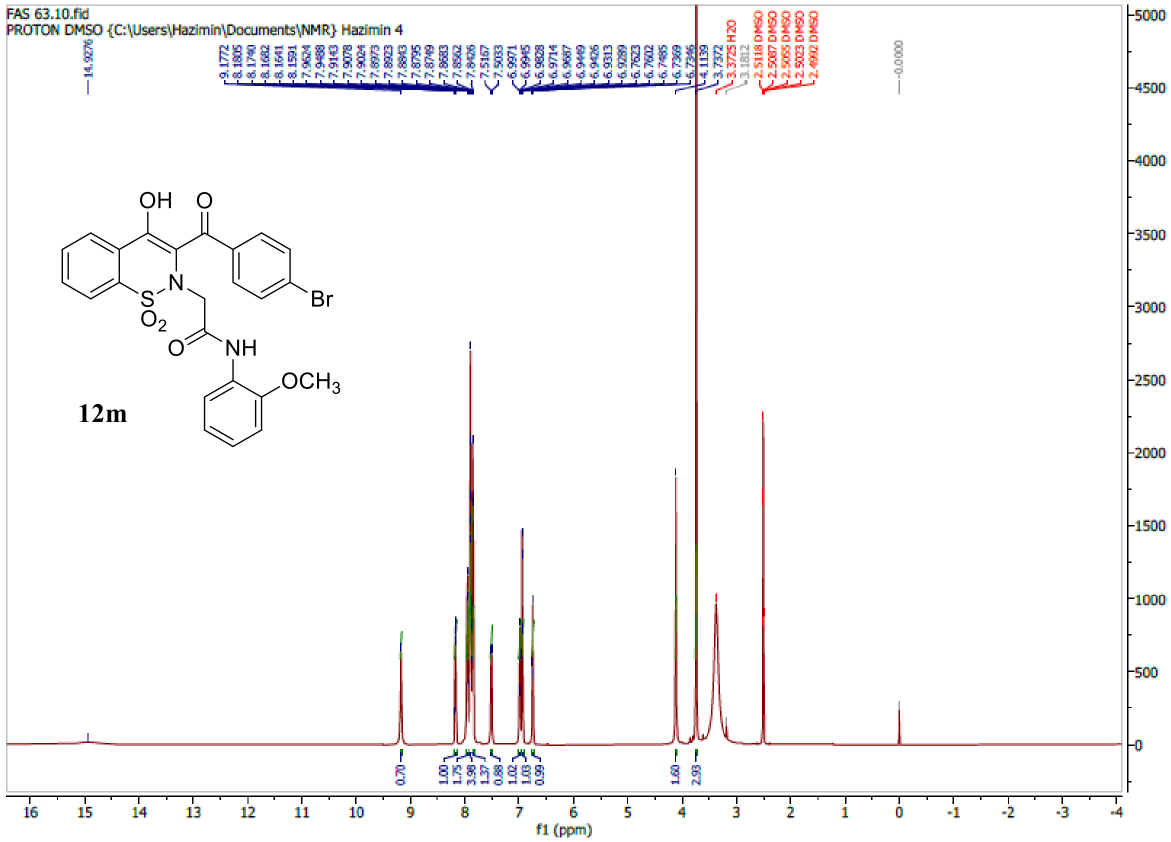

^{1}H NMR Spectrum of compound **12m**.

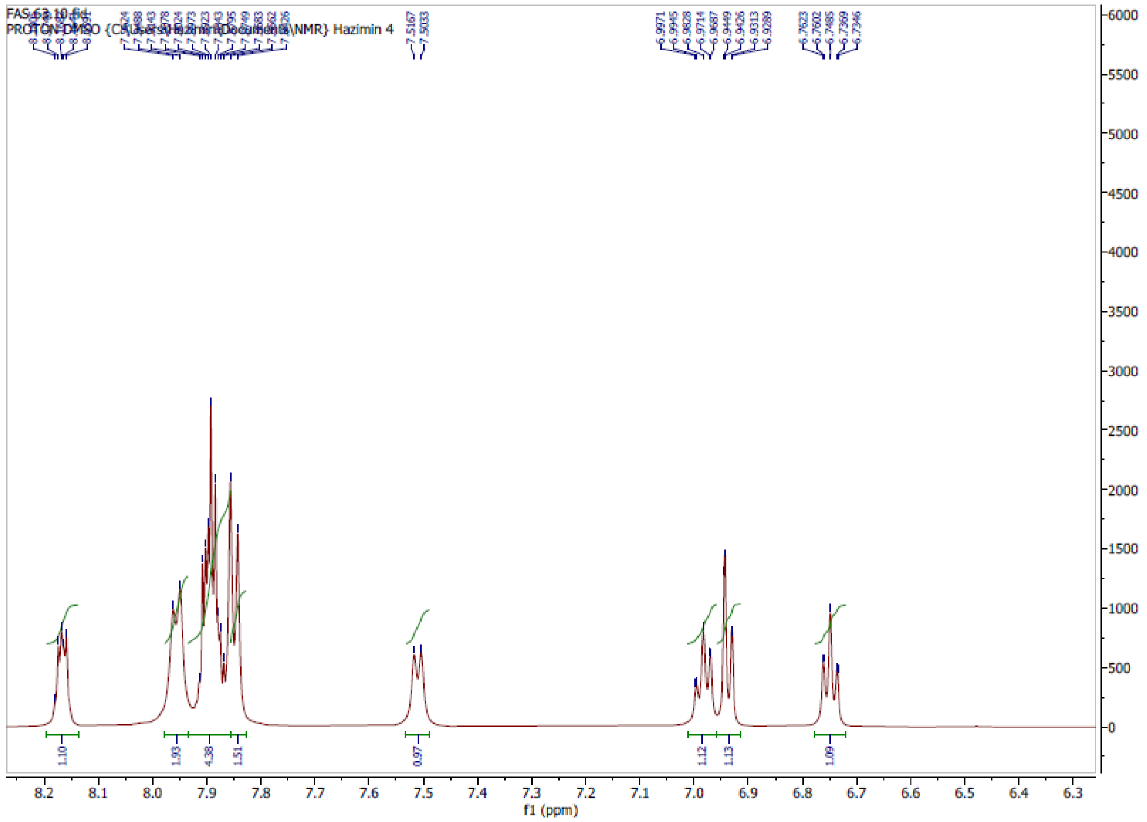

Expanded form of ^{1}H NMR Spectrum of compound **12m** (region 6.70-7.60 ppm).

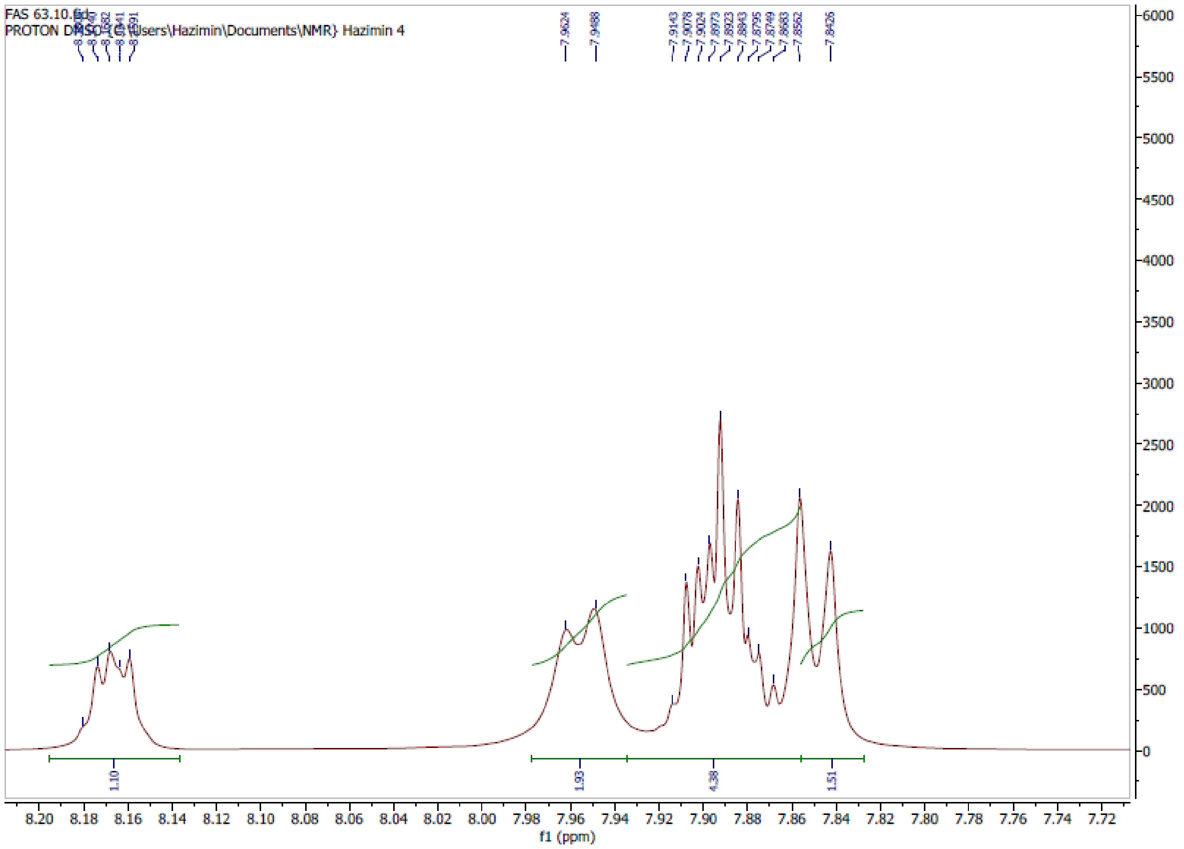

Expanded form of ^{1}H NMR Spectrum of compound **12m** (region 7.82-8.20 ppm).

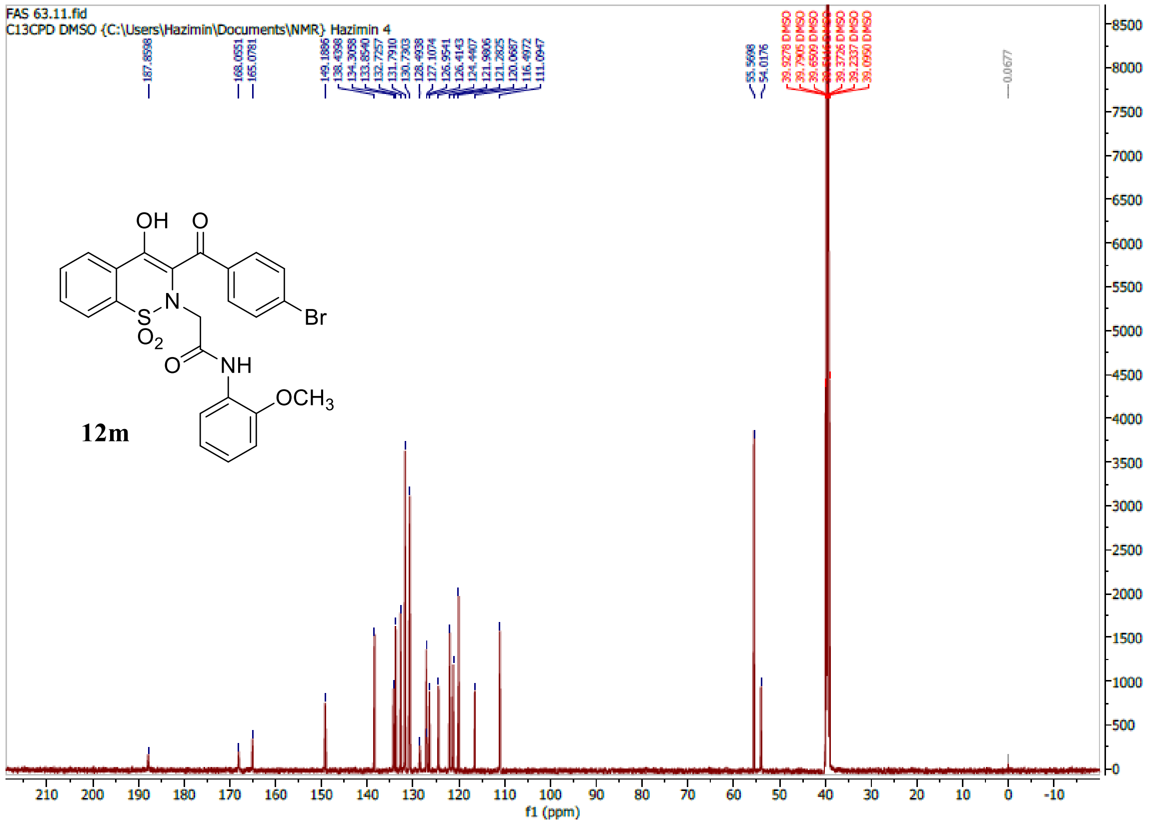

^{13}C NMR Spectrum of compound **12m.**

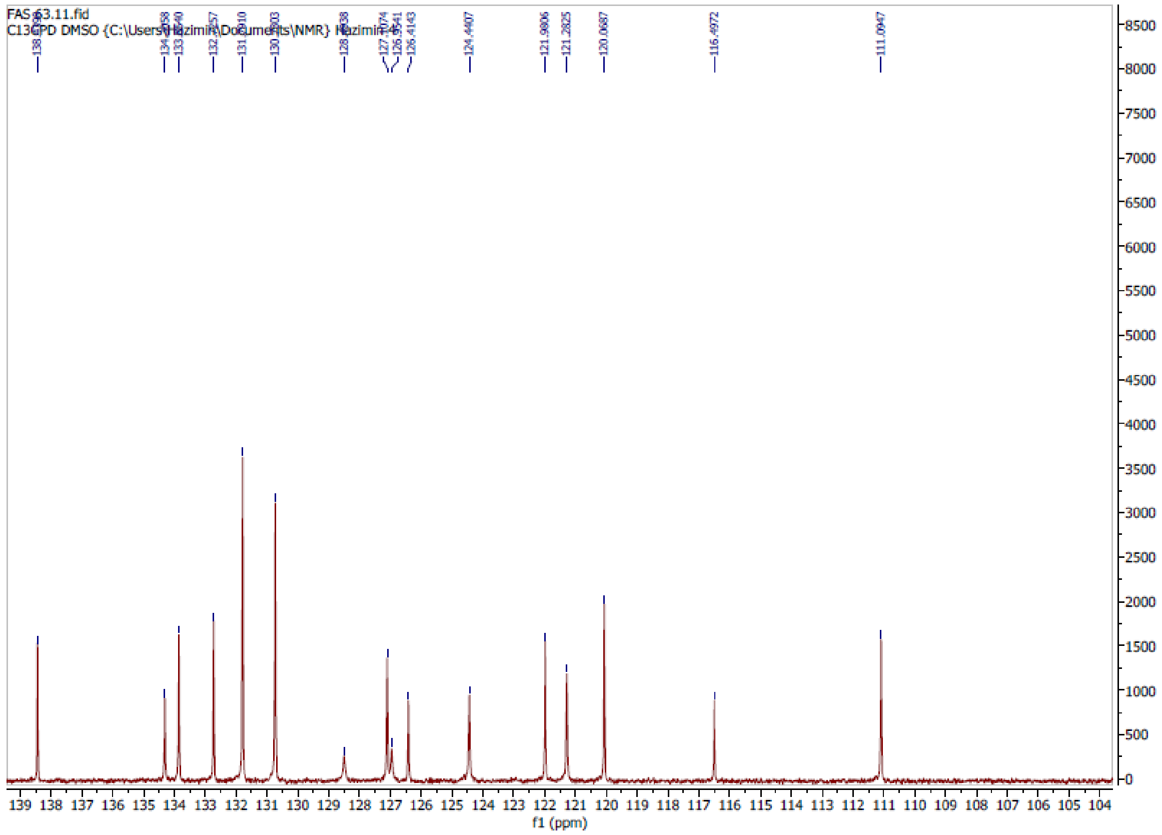

Expanded form of ^{13}C NMR Spectrum of compound **12m** (region 110-139 ppm).

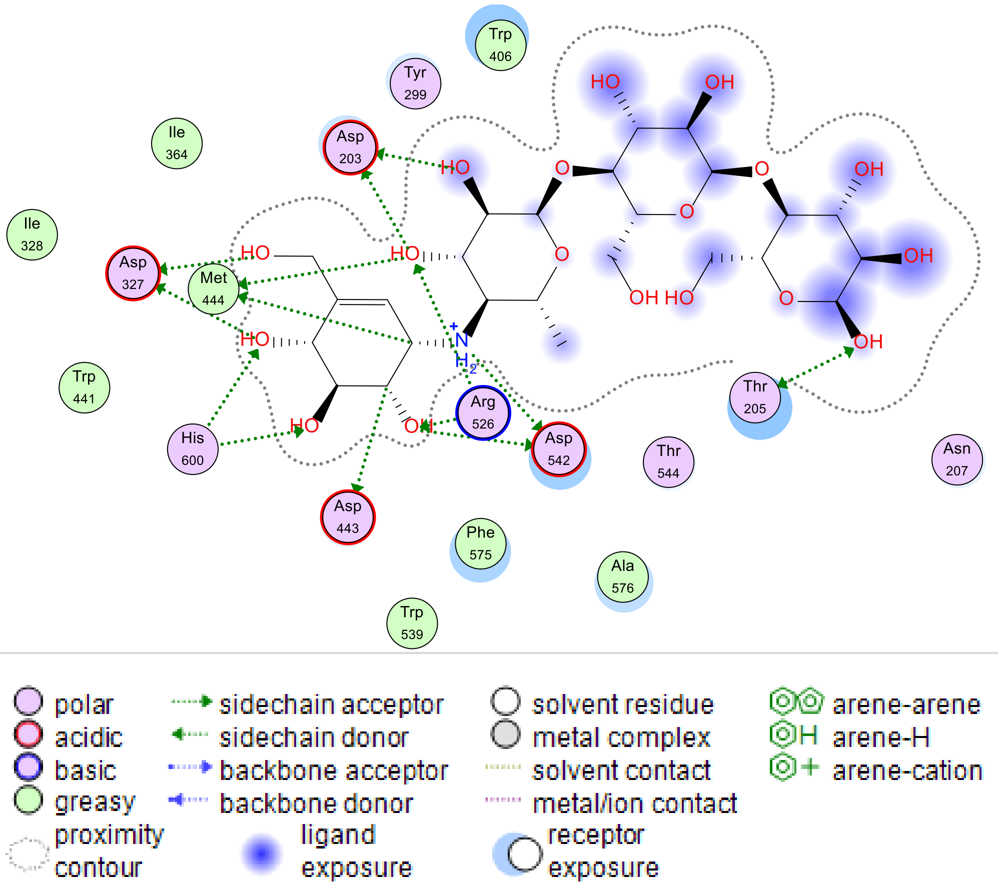

Figure S1. Selected (reference) residues. Computed acarbose interactions.

Table S1. 2D and 3D interaction modes of compounds **9a,b; 11(a,b,d-m)** and **12(b,c,f,h-m)**. In 2D interaction modes the blue color indicates ligand exposure and green color dotted line indicates the interactions of receptor enzyme with ligands. In 3D interaction modes the colored portions show pocket selected and red portion indicates the ligand exposure points.

Comp.	2D-interaction mods	3D-maps
**9a**	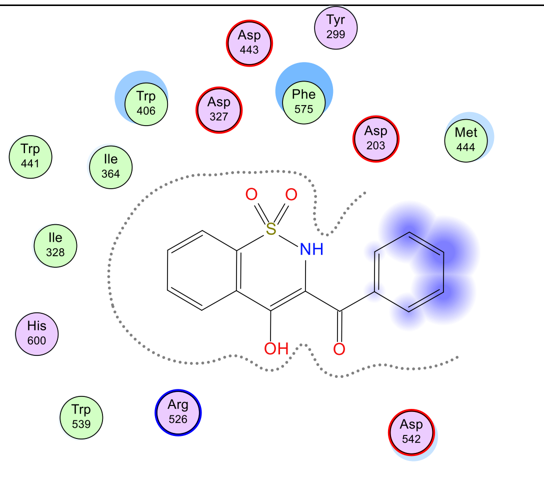	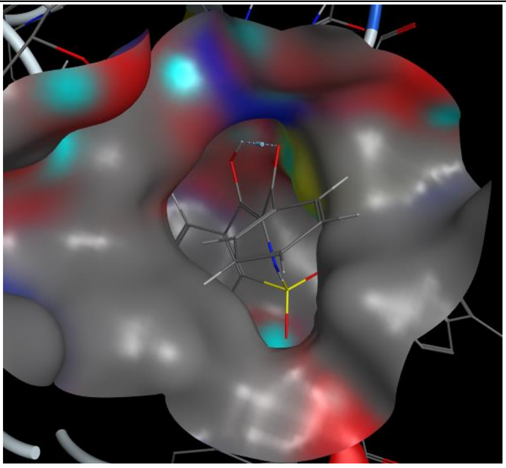

**9b**	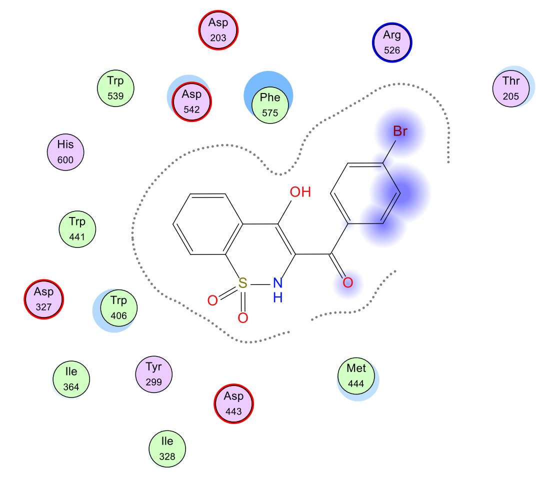	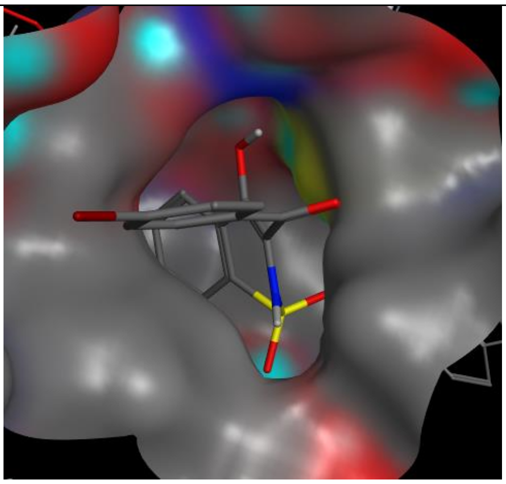
**11a**	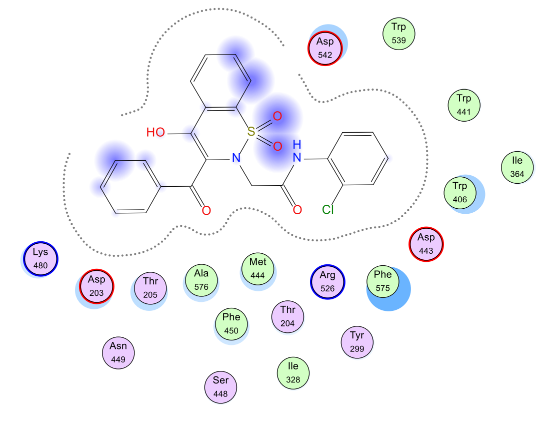	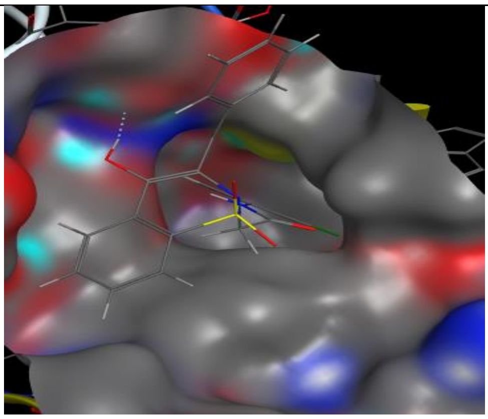
**11b**	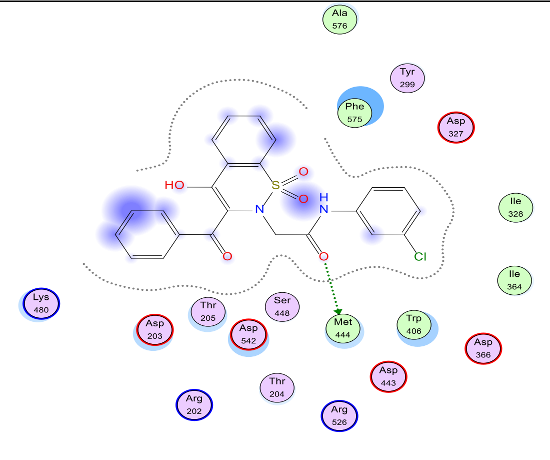	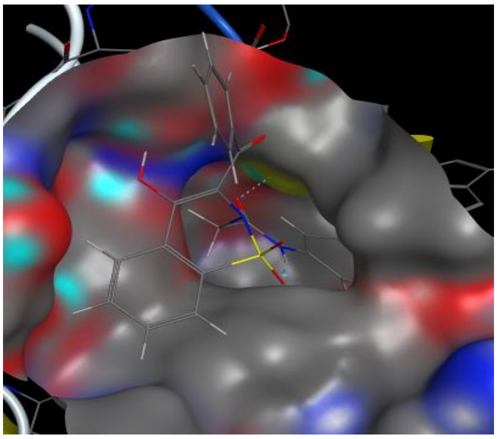
**11d**	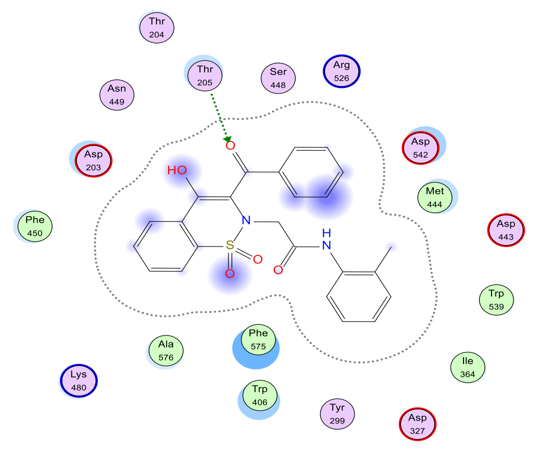	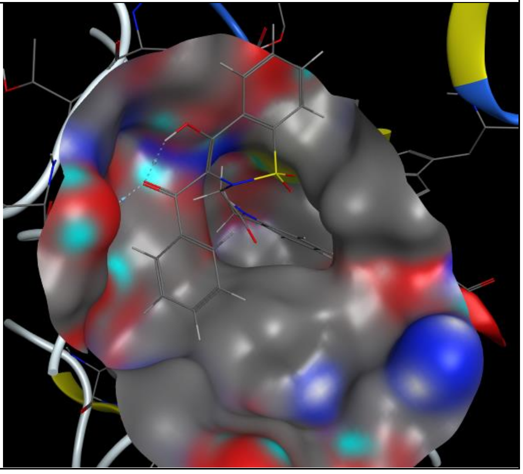

11e

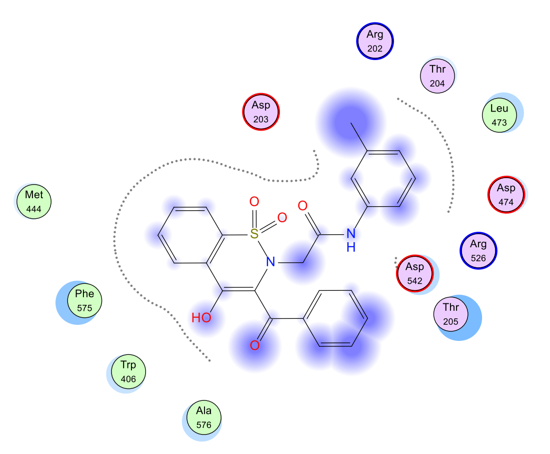

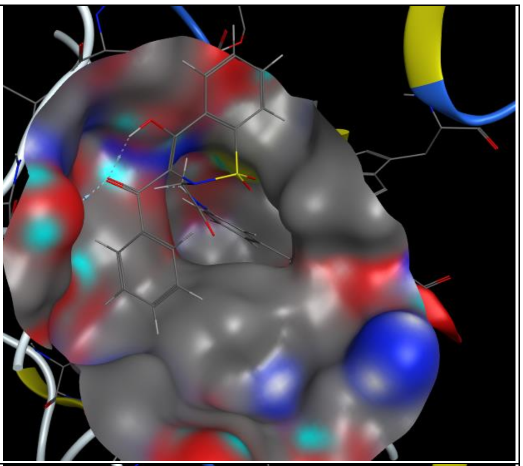

11f

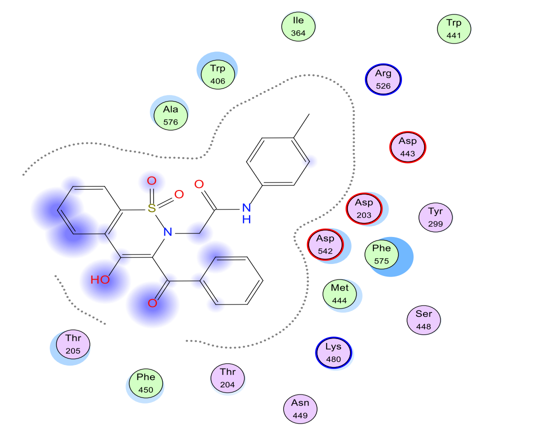

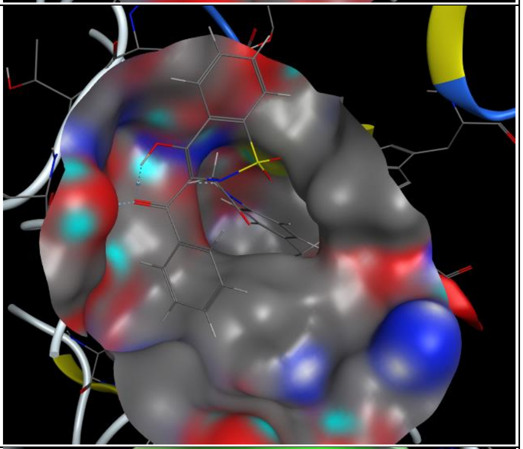

11g

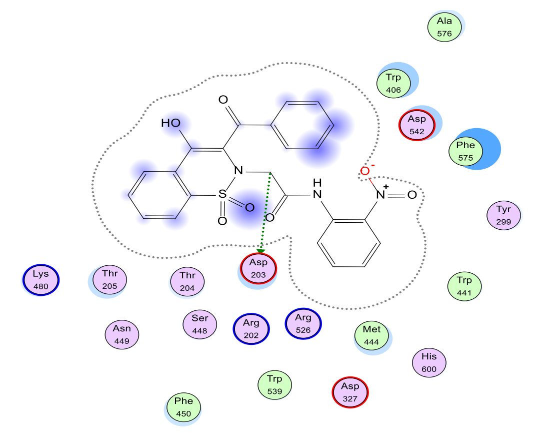

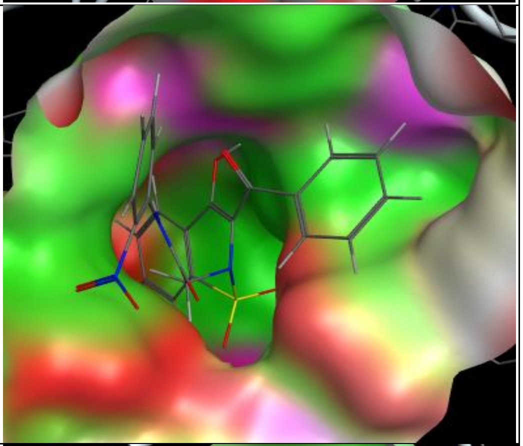

11h

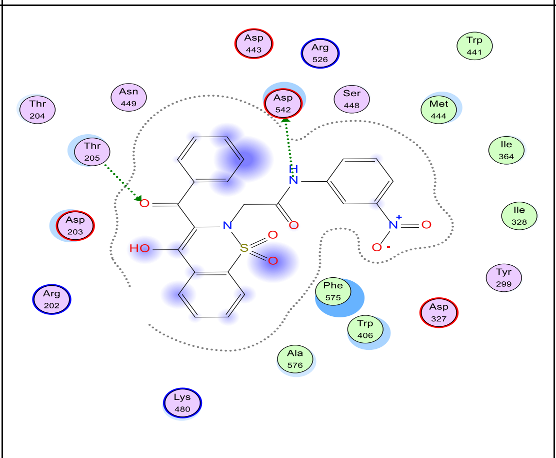

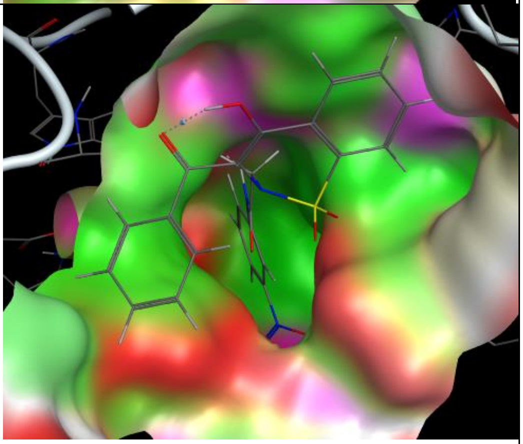

11i

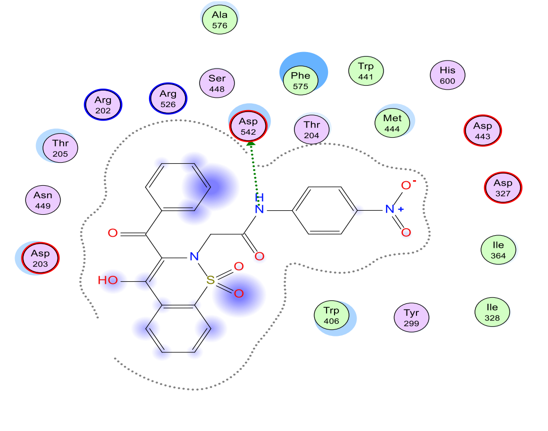

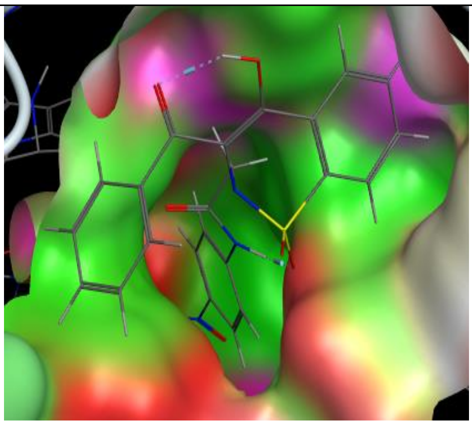

11j

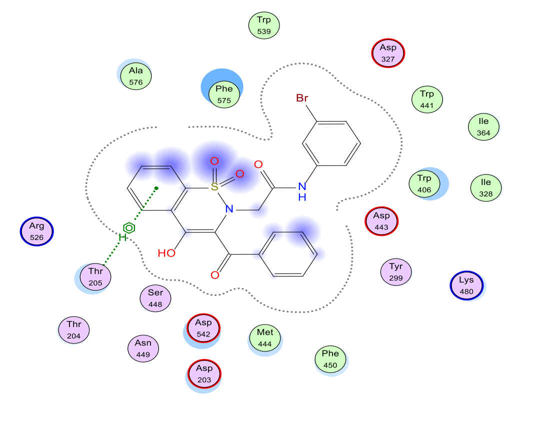

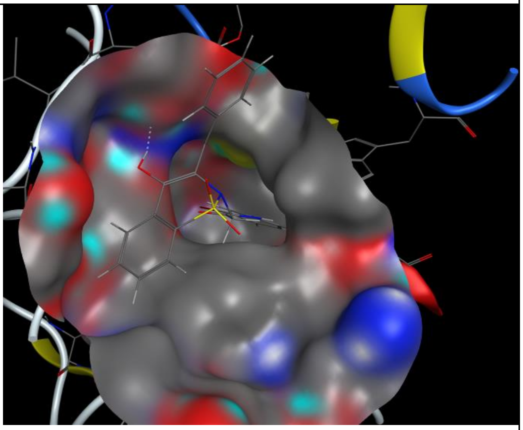

11k

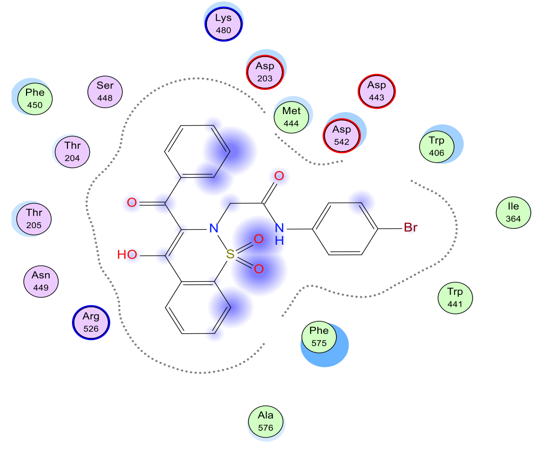

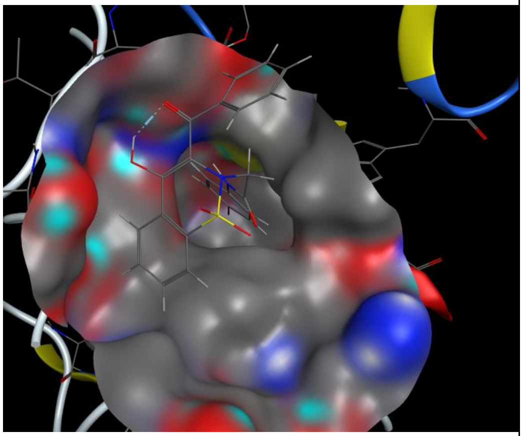

11l

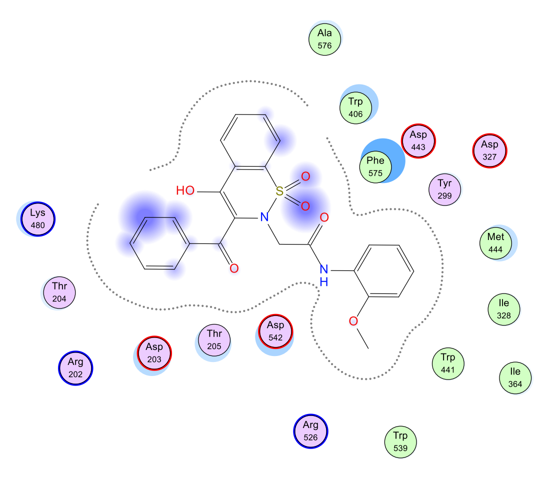

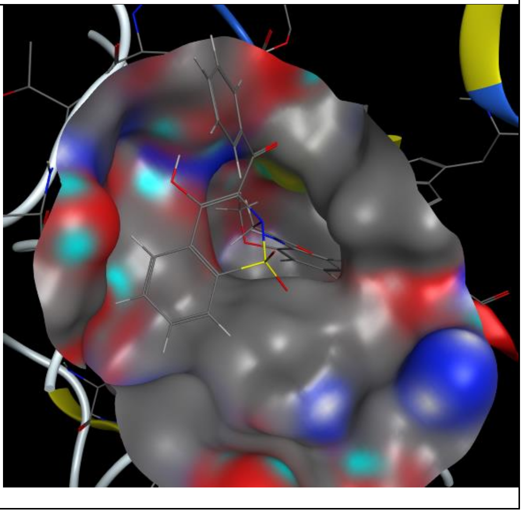

**11m**	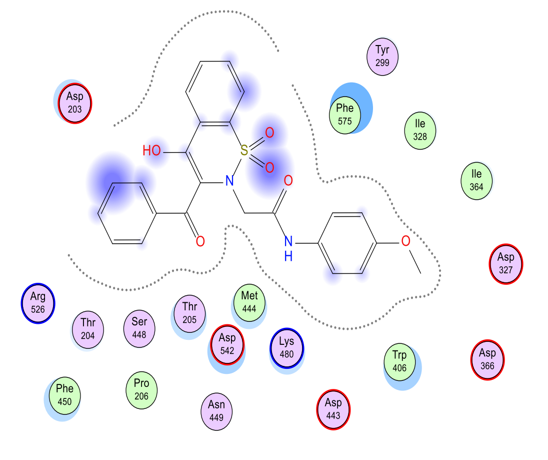	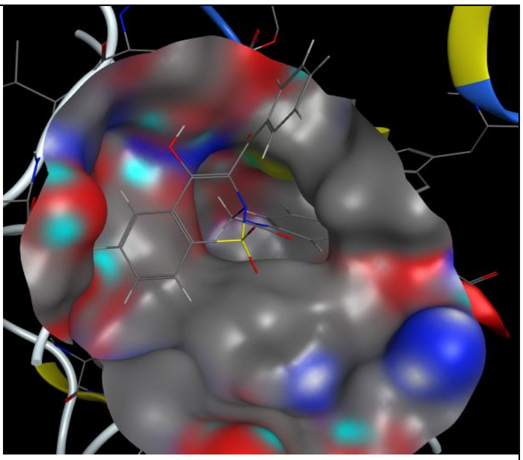
**12b**	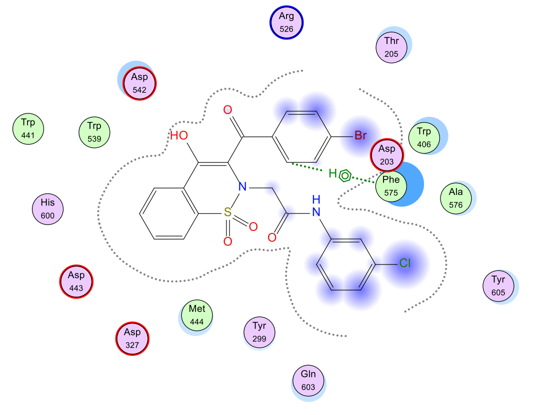	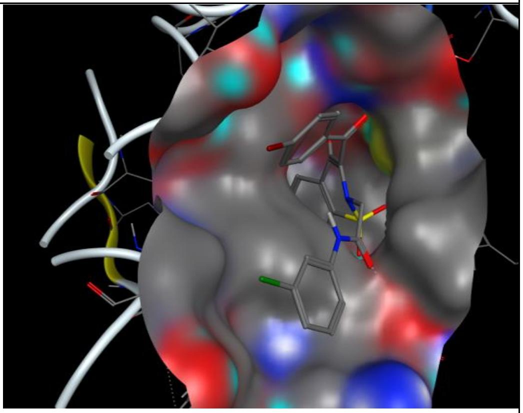
**12c**	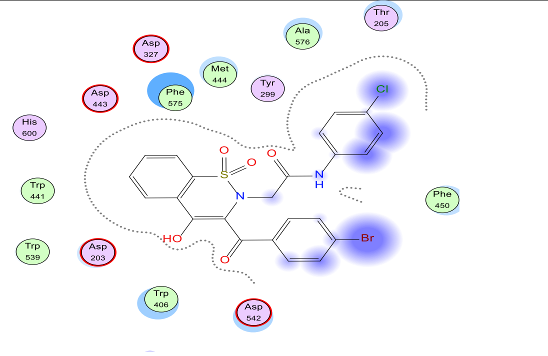	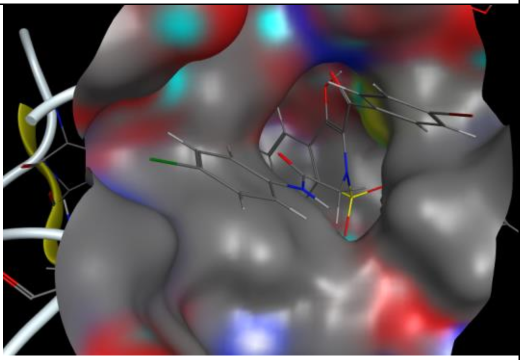
**12f**	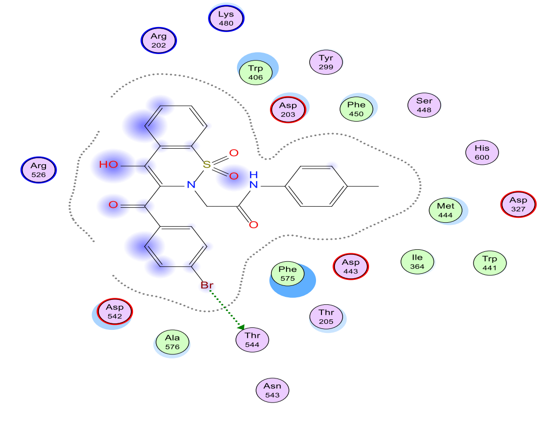	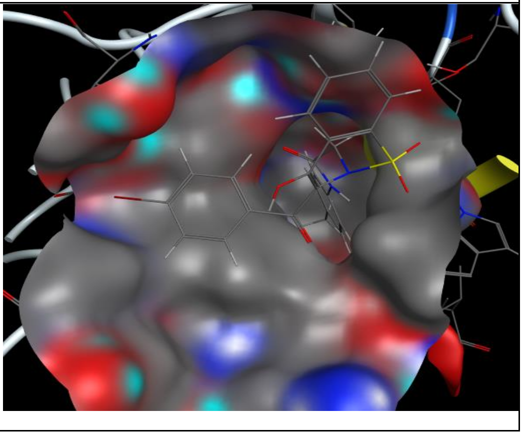

12h

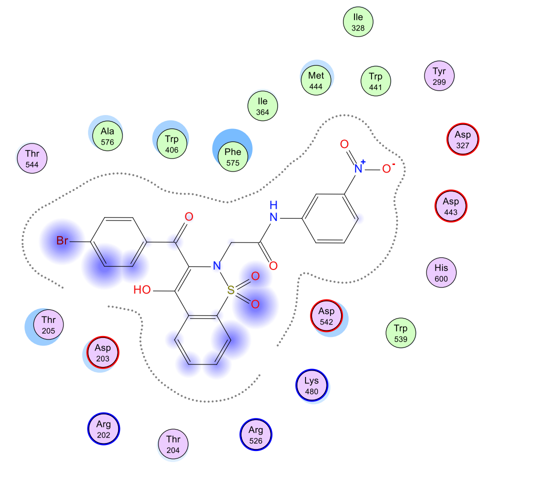

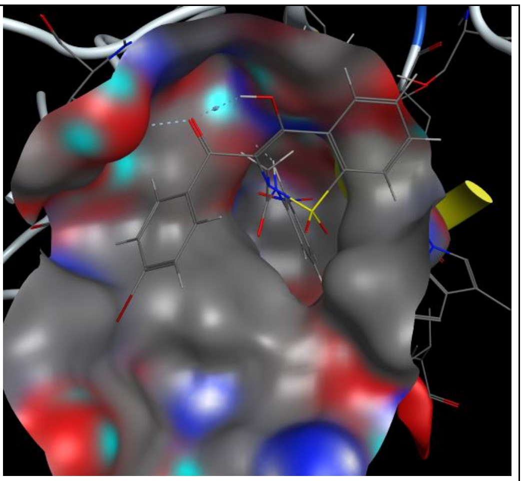

12i

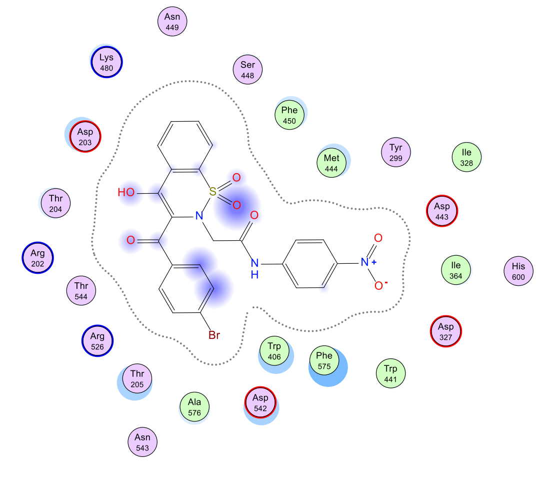

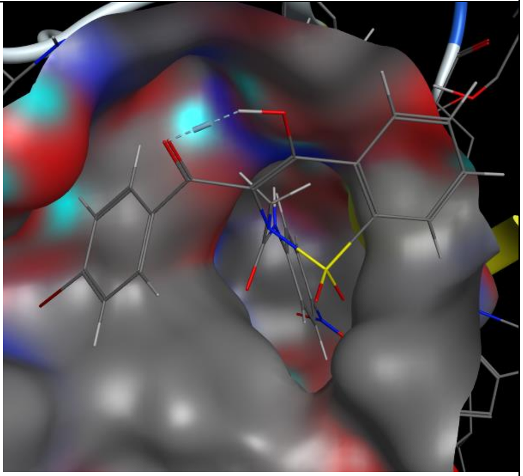

12j

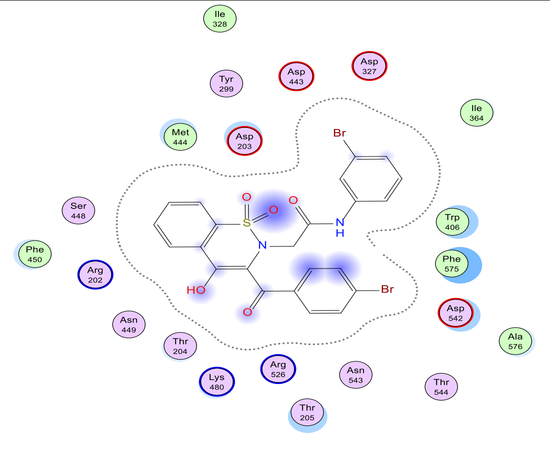

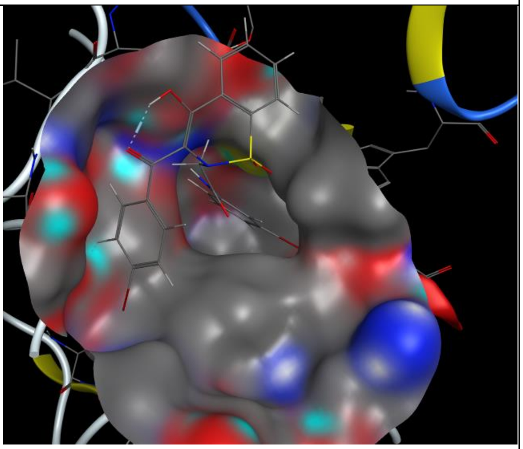

12k

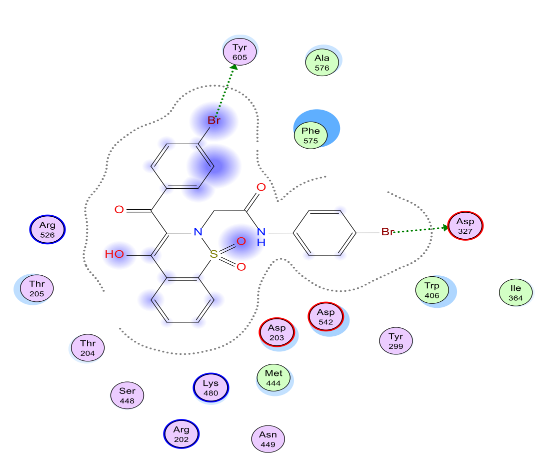

<table>
<tr><td></td><td></td><td>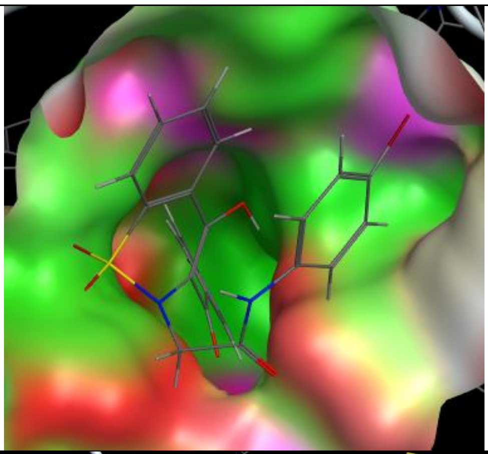</td></tr>
<tr><td>12l</td><td>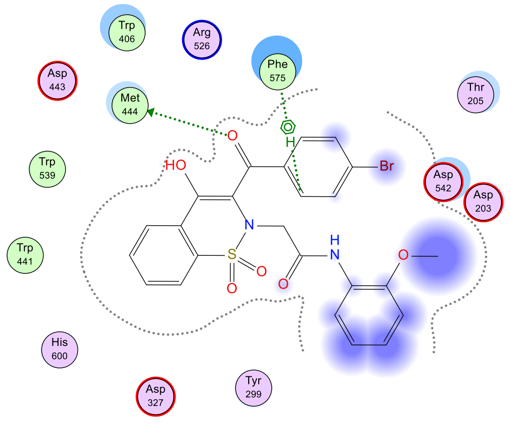
</td><td>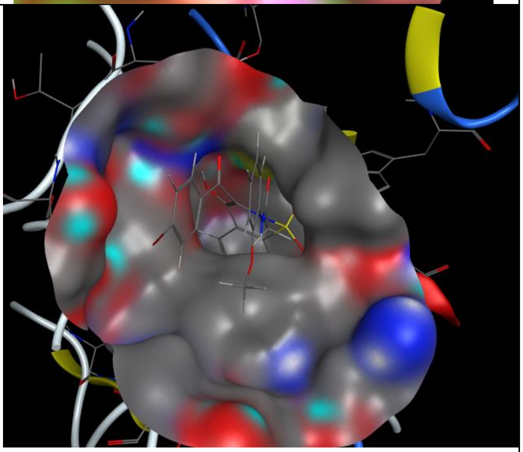</td></tr>
<tr><td>12m</td><td>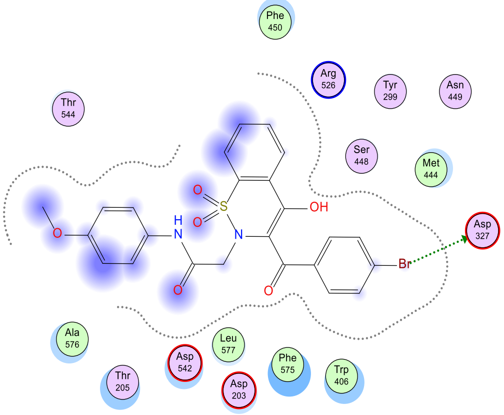
</td><td>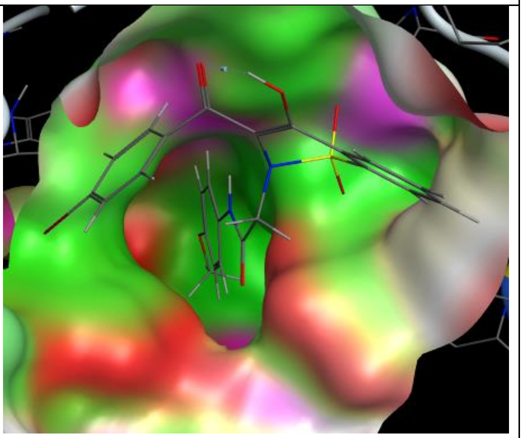</td></tr>
</table>
